# Molecular profiling of repeated self-sampled blood reveals dynamic immune phenotypes in young adults

**DOI:** 10.1038/s41467-026-78987-8

**Published:** 2026-11-14

**Authors:** Annika Bendes, Sophia Björkander, Maura M. Kere, Simon Kebede Merid, Ashish Kumar, Leo Dahl, Tess D. Pottinger, Zhebin Yu, Anna Gardell, Amelie Vogt, Changil Kim, Qiang Pan-Hammarström, Anna Bergström, Inger Kull, Anne-Sophie Merritt, Sandra Ekström, Alexandra Lövquist, Ben Murrell, Niclas Roxhed, Erik Melén, Jochen M. Schwenk

**Affiliations:** 1 Department of Protein Science, SciLifeLab, KTH Royal Institute of Technology, Solna, Sweden; 2 Department of Clinical Science and Education, Södersjukhuset, Karolinska Institute, Stockholm, Sweden; 3 Institute of Environmental Medicine, Karolinska Institute, Stockholm, Sweden; 4 Department of Microbiology, Tumor and Cell Biology, Karolinska Institute, Stockholm, Sweden; 5 Department of Medical Biochemistry and Biophysics, Division of Immunology, Karolinska Institute, Stockholm, Sweden; 6 Centre for Occupational and Environmental Medicine, Region Stockholm, Stockholm, Sweden; 7 Sachs’ Children and Youth Hospital, Södersjukhuset, Stockholm, Sweden; 8 Department of Intelligent Systems, KTH Royal Institute of Technology, Stockholm, Sweden; 9 MedTechLabs, BioClinicum, Karolinska University Hospital, Solna, Sweden

**Keywords:** Proteomic analysis, Infection, Biomarkers

## Abstract

Although population-based studies of circulating blood proteins have provided profound insight into human biology, transient changes to time-dependent exposures often remain elusive. Here, we perform a longitudinal dried blood spot (DBS) self-sampling study in 808 young adults (aged 24–27, 64% females) from the Swedish Barn [Children], Allergy, Milieu, Stockholm Epidemiology cohort. In total, we collected ~2400 samples from 2020 to 2022. Each sample was profiled for SARS-CoV-2 serological, anti-interferon autoantibodies (AAbs), and 365 inflammation-related proteins. We subsequently linked these samples to registry-confirmed infection and vaccination exposures, physiological traits, genetic variation, and blood counts. We show that data-driven seroclustering captures the response to both infection and vaccination exposures, while stable AAbs against interferon receptors IFNAR1 and IFNGR2 associate with the duration of self-reported COVID-19 symptoms. Genome-wide mapping reveals 664 protein quantitative trait loci (pQTL) across 65 proteins, with *cis*-pQTLs associated proteins showing greater longitudinal stability. Paired pre- and post-exposure samples indicate temporal changes in protein levels after infection (LAP3) and vaccination (TIMP3). This multi-molecular epidemiological profiling of self-sampled blood captures dynamic immune trajectories and shorter-term variability in a population of young adults.

## Abstract (web summary)

In this study, the authors used repeated at-home blood sampling to track changes in the immune-related protein during the recent pandemic. The study reveals shorter-lived shifts alongside stable proteins, providing insights missed by standard one-time blood tests

## Introduction

Recent advances in molecular phenotyping have provided new insights into human health at the population level^1–3^. However, clinical outcomes can often be altered by short-term exposures, disproportionately affecting those with preexisting health conditions. Hence, baseline characteristics and continuous insights are needed to interpret preexisting, temporal, and acquired molecular phenotypes.

Understanding the landscape of multi-molecular phenotypes in the general and younger populations will add relevant data to epidemiological investigations, aiding precision medicine approaches on how to treat patients optimally. As young adults become older, they are set to require care later in life. Thus, understanding and managing the relationships among existing, shorter, transient or acquired health conditions will support countermeasures against upcoming chronic diseases. To enable large-scale monitoring in a more patient-centric manner, self-sampling of dried blood spots (DBS) has become increasingly attractive as a valuable alternative to venous blood draws. By simplifying access to samples, new types of molecular data can be generated without in-clinic visits, contributing to democratizing health care. Benchmarking novel multiparameter DBS profiling assays against established routines is the subject of current efforts to ensure their accuracy and reproducibility^4–6^.

Among the most accessible and informative molecular phenotypes, circulating blood proteins offer attractive insights into genetics, environmental exposures, comorbidities, and treatment effects, reporting on a broad range of biological processes^7^. For example, immunoglobulins are well-established markers of immunological memory, and acquired anti-SARS-CoV-2 antibodies (Abs) can persist over years^8,9^. Infections have also been linked to the onset of autoantibodies (AAbs), while other changes in the circulating blood proteome^10^, such as cytokines^11^ and immune cell receptor activation^12^, are more short-lived. Compared to pathogen-specific Abs, inflammatory proteins may be activated by different exposures and at different timepoints during a person’s life. Therefore, integrating both immune and proteomic profiling is informative for capturing the host response to infection, providing insights into immune variability, risk stratification, and persisting health consequences of infections and vaccinations.

Here, we performed a comprehensive longitudinal DBS self-sampling study of 800 young adults in a population-based birth cohort to provide insights into immune resilience and susceptibility in young adults. Our primary objectives were (a) to demonstrate feasibility of DBS sample use for longitudinal multi-molecular profiling, (b) to study dynamic trajectories of anti-SARS-CoV-2 antibodies, autoantibodies and associated protein biomarkers in a real-life, population-based setting, and (c) to link immune and protein biomarkers with clinical outcomes, including long-term COVID-19 symptoms. By combining multi-analyte serology, autoimmune and proteomic data with genetic, clinical, registry and health history data, we revealed transient and persistent trajectories, highlighting molecular phenotypes before, during, and after SARS-CoV-2 exposure. Collectively, our results establish an actionable framework for remote molecular health monitoring, aiding epidemiological and mechanistic investigations towards adjustable therapeutic management, highlighting the importance of the timing and feasibility of remote sample collection.

## Results

**Fig. 1:**
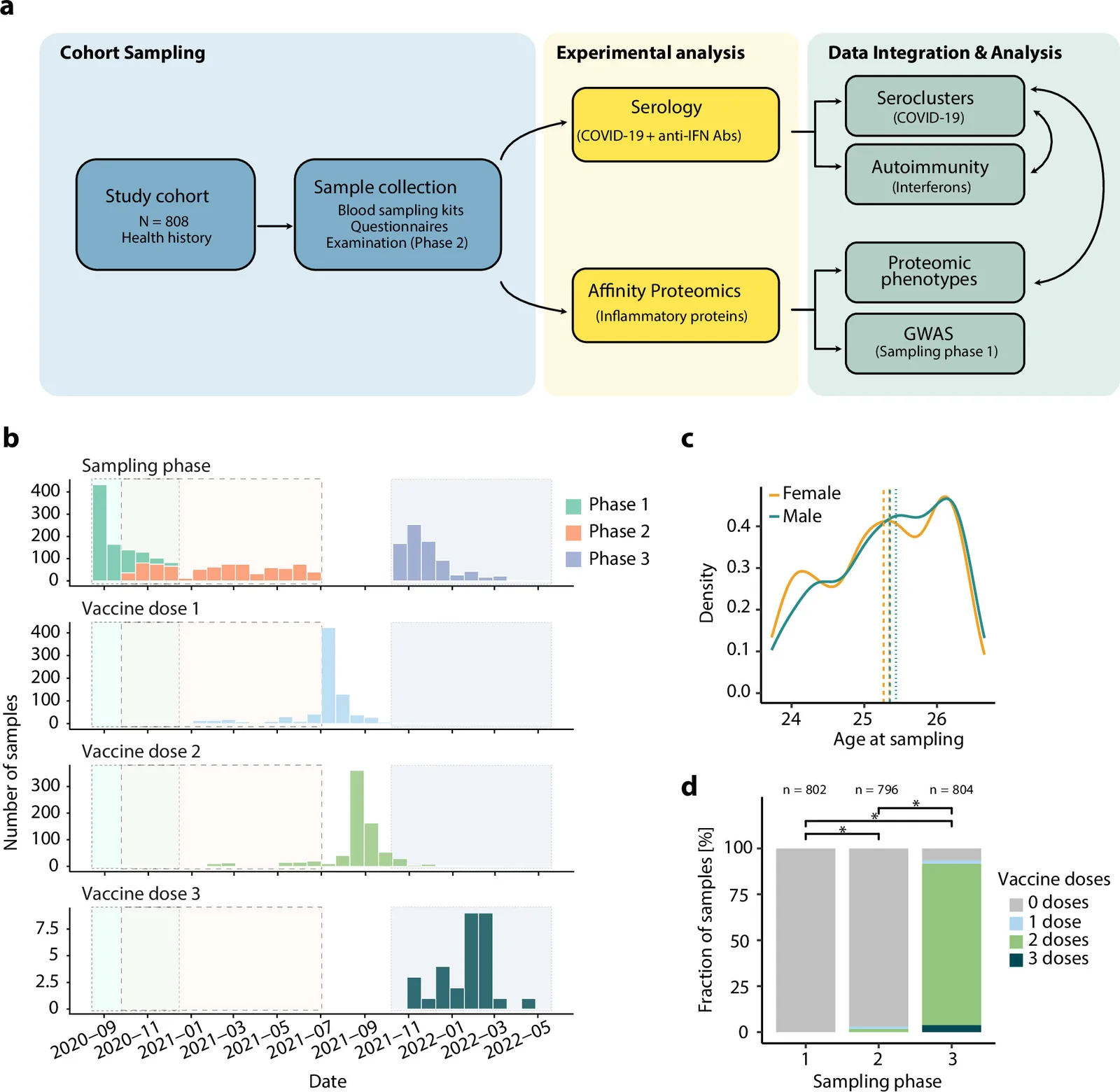
Study design for trans-pandemic analysis of dried blood spots. **a** Study design in which 808 participants were asked to give their blood sample at three different time points (sampling phases) and answer questionnaires. At sampling Phase 2, the participants underwent a clinical examination. The dried blood spot samples were subsequently subjected to serological analyses with antibodies against COVID-19 proteins and human interferons and proteomic analyses with 365 inflammatory proteins. **b** The bar plots show when the samples were taken between August 2020 and February 2022, in relation to the sampling phases (teal = 1; orange = 2; steel blue = 3) and number of vaccine doses (light blue = 1; olive green = 2; dark teal = 3). Phase 3 was predominantly sampled after two vaccine doses. **c** Age distribution of males and females at sampling phase 1 (vertical dashed and dotted lines indicates mean and median, respectively). **d** Distribution of vaccinations per sampling phase. Asterisks represent FDR-adjusted *p* < 0.05 according to two-sided Fisher’s exact test. For Phase 1~2, this was *p* = 4.7×10^−8^, while *p*-values from the test with Phase 3 fell below the limit of computational precision (*p* < 2.2×10^−16^). The colors indicate the number of vaccine doses (grey = 0; light blue = 1; olive green = 2; dark teal = 3).

We studied self-sampled DBS from young adults to investigate the impact of SARS-CoV-2 infection and vaccination on their molecular phenotypes (Fig. 1a). The goal was to evaluate the feasibility of deconvoluting how molecular health information is impacted by pandemic exposure and how this information shapes the dynamic multianalyte phenotypes of the participants.

### Study design and cohort population

From the BAMSE birth cohort, 808 participants (64% females) completed a longitudinal study consisting of three blood self-sampling phases from August 2020 to May 2022 (Fig. 1b)^13^. In addition, participants answered health-related questionnaires for Phases 1–3, and in Phase 2, participants also underwent a clinical examination. There were no differences in age between the sexes, but a greater proportion of females smoked cigarettes, and males had a higher BMI, while females had a greater body fat percentage (Fig. 1c, and Table 1). Of the 808 participants, 791 (97.9%) successfully provided one quantitative DBS sample per phase. In Phase 1, no participants were vaccinated; 3.1% had received at least one dose in Phase 2, and 93.4% had received it in Phase 3 (Fig. 1d). In Phase 1, 1.4% of the participants reported PCR-confirmed infections, 9.7% in Phase 2, and 18.9% in Phase 3 (Fig. S1, and Table 1), where the Omicron wave added the highest number of new cases.

**Table 1:** Overall demographics per sampling phase of the 808 subjects in the study population^a,b^

Phase 1	All	Females	Males	Nominal *p* value (sex)
Sex (%)	808	517 (64.0)	291 (36.0)	NA
Median Age (range) [years]	25.4 (23.7–26.7)	25.3 (23.7–26.5)	25.4 (23.7–26.7)	0.141
Smoking (%)	112 (13.9)	83 (16.1)	29 (10.0)	0.019
Median BMI (range) [kg/m^2^]	–	–	–	NA
BMI categorical	–	–	–	NA
BMI < 25.0 (%)	–	–	–	
BMI ≥ 25.0 (%)	–	–	–	
Body fat percentage	–	–	–	NA
SmiNet case (%)^c^	11 (1.4)	7 (1.4)	4 (1.4)	1.000
Vaccinated COVID-19 (%)^d^	0 (0.0)	0 (0.0)	0 (0.0)	NA
Number of vaccine doses	–	–	–	NA
One vaccine dose only (%)	–	–	–	
Two vaccine doses only (%)	–	–	–	
Three vaccine doses (%)	–	–	–	

Phase 2	All	Females	Males	Nominal *p* value (sex)
Median Age (range) [years]	25.9 (23.9–27.3)	25.8 (23.9–27.3)	25.9 (24.0–27.2)	0.178
Smoking (%)	88 (10.9)	68 (13.2)	20 (6.9)	0.007
Median BMI (range) [kg/m^2^]	22.6 (14.8–59.9)	22.1 (14.8–59.9)	23.5 (16.8–48.3)	2.72×10^−7^
BMI categorical	–	–	–	1.71×10^−4^
BMI < 25.0 (%)	603 (75.4)	406 (79.8)	197 (67.7)	
BMI ≥ 25.0 (%)	197 (24.6)	103 (20.2)	94 (32.3)	
Body fat percentage	23.4 (5.9–51.1)	26.1 (11.6–51.1)	17.5 (5.9–43.8)	4.79×10^−59^
SmiNet case (yes)^c^	78 (9.7)	53 (10.3)	25 (8.6)	0.535
Vaccinated COVID-19 (yes)^d^	25 (3.1)	17 (3.3)	8 (2.8)	0.833
Number of vaccine doses	–	–	–	0.613
One vaccine dose only (%)	12 (1.5)	7 (1.4)	5 (1.7)	
Two vaccine doses only (%)	13 (1.6)	10 (1.9)	3 (1.0)	
Three vaccine doses (%)	0 (0.0)	0 (0.0)	0 (0.0)	

Phase 3	All	Females	Males	Nominal *p* value (sex)
Sex (%)	808	517 (64.0)	291 (36.0)	ND
Median Age (range) [years]	26.6 (24.9–27.8)	26.5 (24.9–27.8)	26.6 (24.9–27.7)	0.165
Smoking (%)	81 (10.1)	60 (11.7)	21 (7.3)	0.051
Median BMI (range) [kg/m^2^]	–	–	–	NA
BMI categorical	–	–	–	NA
BMI < 25.0 (%)	–	–	–	
BMI ≥ 25.0 (%)	–	–	–	
Body fat percentage	–	–	–	NA
SmiNet case (yes)^c^	152 (18.9)	104 (20.2)	48 (16.5)	0.223
Vaccinated COVID-19 (yes)^d^	753 (93.4)	482 (93.6)	271 (93.1)	0.883
Number of vaccine doses	–	–	–	0.817
One vaccine dose only (%)	17 (2.1)	12 (2.3)	5 (1.7)	
Two vaccine doses only (%)	708 (87.8)	454 (88.2)	254 (87.3)	
Three vaccine doses (%)	28 (3.5)	16 (3.1)	12 (4.1)	

Continuous variables were analyzed by the two-sided Mann‒Whitney U test and are displayed as medians with ranges. Dichotomous variables were analyzed by two-sided Fisher´s exact test and are displayed as numbers and percentages.

^a^All subjects with at least one approved serology or Olink measurement from at least one phase.

^b^One female subject lacked smoking status in Phase 1, one male subject lacked smoking status in Phase 2, eight female subjects lacked BMI and body fat percentage in Phase 2, two females and three males lacked smoking status in Phase 3, and two female subjects lacked SmiNet case and COVID-19 vaccination information in Phase 3 due to a lack of data from filter paper sampling.

^c^SmiNet (a national register for notifiable transmittable diseases in Sweden) covering all positive PCR tests (Public Health Agency of Sweden. SmiNet. In Swedish. Available at: www.folkhalsomyndigheten.se/sminet). A SmiNet case was defined as a positive entry date in SmiNet preceding the date of DBS filter paper sampling in each respective phase.

^d^Information on vaccination against COVID-19 was obtained by linkage to the National Vaccination Register. COVID-19 vaccination was defined as receiving the vaccination before the date of DBS filter paper sampling in each respective phase. NA = not applicable; - = not available.

### Classification of antibody response by multianalyte serology

To assess the antibody response against SARS-CoV-2 over time, we performed bead-based multianalyte serology analysis to detect circulating human IgG antibodies in DBS samples, following our previously validated protocol^14^. Based on population-based cutoffs, 13.9% of the N-seropositive (N+) individuals were in Phase 1, 22.2% were in Phase 2, and 26.0% were in Phase 3 (averaged over several N proteins). The average S-seropositivity (S+) increased from 15.1% to 28.8% to 97.3% across the sampling phases, reflecting infection and vaccination status. Similarly, RBD seropositivity (RBD+) increased from 10.3%, 20.9% to 96.9% (Supplementary Data 1 and 2). These data are consistent with the findings of public health authorities on infection and vaccination rates^15^ and previous studies in a healthcare setting^16^.

### Seroclustering of antibody response by multianalyte serology

**Fig. 2:**
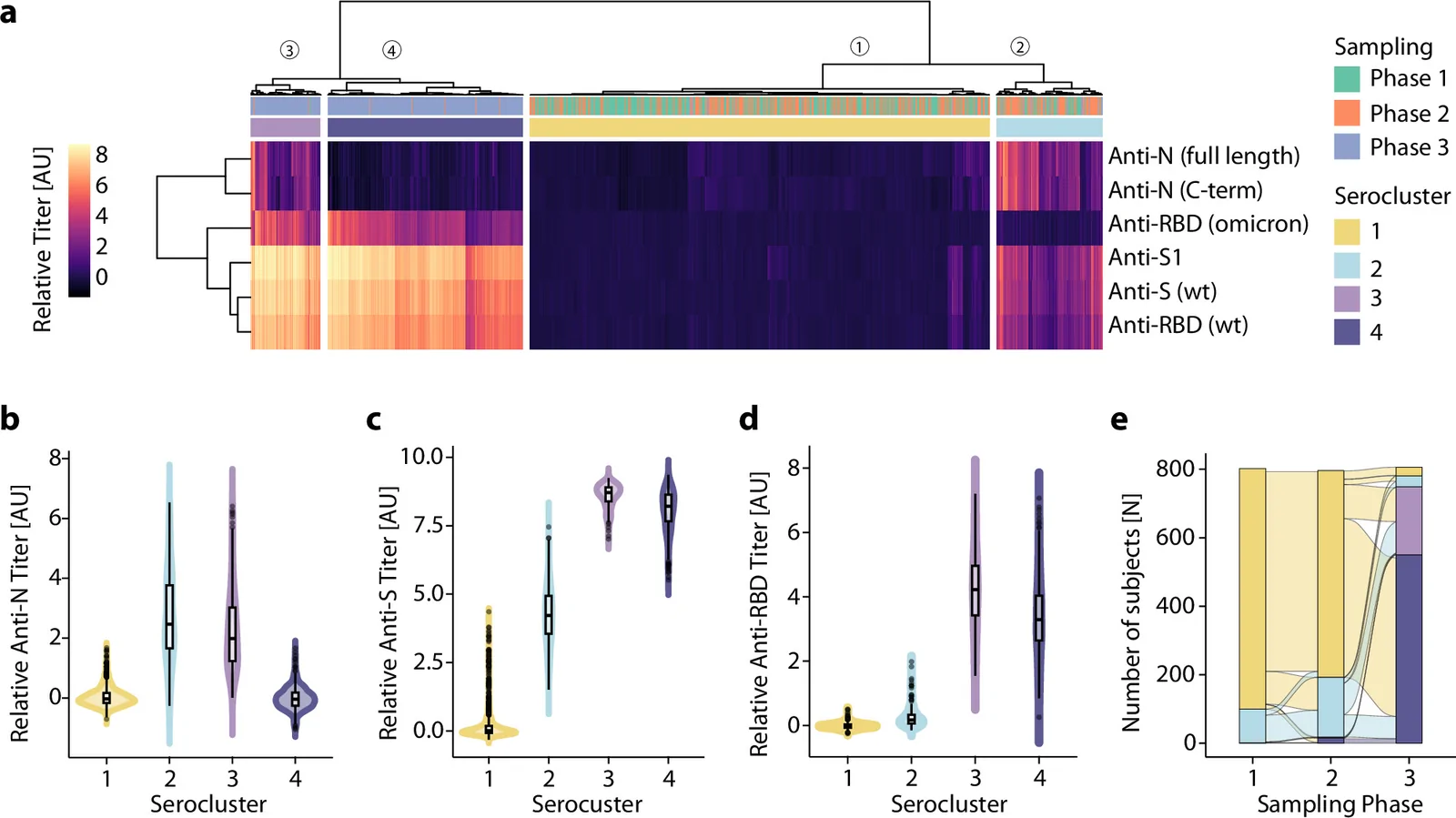
Serological analysis. **a** The heatmap shows the cluster analysis of multianalyte serology assays (2× N, 2× RBD, 2× S proteins) to define four distinct seroclusters. SC1 contained 1220 samples from 722 donors who were deemed seronegative at the time of sampling. SC2 contained 306 samples from 211 donors who were deemed infected. SC3 contained 203 samples from 199 donors who were deemed infected and vaccinated. SC4 contained 565 samples from 552 donors who were deemed vaccinated only. The colored bands indicate the sampling phases (teal = 1; orange = 2; steel blue = 3) Relative titers for (**b**) anti-N, (**c**) anti-S and (**d**) anti-RBD are shown per serocluster. The boxplots show the 25% and 75% quartiles, with the median in the center. Whiskers extend to at most 1.5 x IQR from the boxes, and any values outside this range are shown as points. The violin plots behind the boxes show the density curves. **e** Distribution of seroclusters per sampling phase. In all plots, seroclusters were color-coded as 1 in gold, 2 in light blue, 3 in lavender, and 4 in indigo.

To capture the heterogeneity of the immune response, considering intermediate or partial responders, we used data-driven clustering to stratify the samples into four distinct seroclusters based on serology (Fig. 2a), confirming previous insights^17^. As shown in Fig. 2a–d, Serocluster 1 consisted predominantly of seronegative samples (N–, S–, and RBD–), while Serocluster 2 had an average of 96.9% S+ but slightly lower positivity rates for N+ (89.4%) and RBD+ (74.2%). RBD+ rates were lower for the Omicron variants BA.1 and BA2 (21.2%) than for the earlier RBD versions (89.4%). Serocluster 3 contained ≥90% of the seropositive samples, with an average of 84.6% N+, of which were deemed S+ or RBD+. All the samples in Serocluster 3 were seropositive for both Omicron variants. Finally, Serocluster 4 was primarily S+ (100%) and RBD+ (99.9%), with only 3.2% being N+.

Serocluster 1 samples were collected mostly during Phase 1 and Phase 2 (Fig. 2e), prior to the start of vaccinations in the general population in Sweden. Within these phases, there were no differences in age, sex or smoking status between the seroclusters (Table 2). Among the 99 S+ individuals in Serocluster 2 from Phase 1, 84.9% reported symptoms of suspected or confirmed COVID-19 since February 2020, whereas 38.5% of those in Serocluster 1 did. Furthermore, there were significantly greater proportions of subjects reporting a positive antibody or PCR test (43.3%) and having a confirmed infection, defined as a SmiNet case (11.1%), in Serocluster 2 than in Serocluster 1. A similar pattern was observed in Phase 2, where compared with individuals in the other clusters, those in Serocluster 2 had the highest proportions of individuals with self-reported PCR or antibody tests (42.9%) and SmiNet-confirmed infection (38.9%) (Table 2).

**Table 2:** Serocluster demographics per sampling phase

Phase 1 (*n* = 802)					
Serocluster	1	2	3	4	Nominal *p* value
*n*	702	99	1	0	ND
Female (%)	455 (64.8)	57 (57.6)	–	–	0.180
Age (years)	25.4 (23.7–26.7)	25.3 (23.7–26.5)	–	–	0.494
Smoking (%)^a^	91 (13.0)	20 (20.2)	–	–	0.062
Symptoms (% since February 2020)	269 (38.5)	84 (84.9)	–	–	1.03×10^−18^
Symptoms (% since August 2020)	–	–	–	–	NA
Symptoms ≥ 2 months^a^ (%)	10/268 (3.7)	2/84 (2.4)	–	–	0.738
Symptoms ≥ 3 months^a^ (%)	–	–	–	–	NA
Self-reported positive antibody-test (%)	3 (0.4)	35 (35.4)	–	–	2.41×10^−31^
Self-reported positive PCR-test (%)	1 (0.1)	10 (10.1)	–	–	5.43×10^−9^
Self-reported positive PCR- or antibody-test (%)	4 (0.6)	43 (43.3)	–	–	7.61×10^−39^
SmiNet-case (%)^b^	0 (0.0)	11 (11.1)	–	–	6.19×10^−11^
Days since SmiNet-case	ND	67 (35–144)	–	–	NA
New SmiNet-case^c^ (%)	ND	ND	–	–	NA
Vaccinated COVID-19^d^ (%)	0 (0.0)	0 (0.0)	–	–	NA
Number of vaccine doses			–	–	NA
Not vaccinated (%)	–	–	–	–	
1 vaccine dose only (%)	–	–	–	–	
2 vaccine doses only (%)	–	–	–	–	
3 vaccine doses (%)	–	–	–	–	
Days since vaccine dose 1	–	–	–	–	NA
Days since vaccine dose 2	–	–	–	–	NA
Days since vaccine dose 3	–	–	–	–	NA
Hospital SARS-CoV-2 serology test positive^e^ (%)	ND	ND	–	–	NA

Phase 2 (*n* = 796)					
n	603	175	3	15	ND
Female (%)	387 (64.2)	110 (62.9)	–	13 (86.7)	0.191
Age (years)	25.9 (24.0–27.2)	25.7 (24.2–27.3)	–	26.4 (25.4–27.6)	0.440
Smoking (%)^a^	64 (10.6)	24 (13.7)	–	0 (0.0)	0.233
Symptoms (% since February 2020)	ND	ND	–	ND	NA
Symptoms (% since August 2020)	247 (41.0)	99 (56.6)	–	89 9 (53.3)	8.95×10^−4^
Symptoms ≥ 2 months^a^ (%)	11/247 (4.5)	4/99 (4.0)	–	1/8 (12.5)	0.49
Symptoms ≥ 3 months^a^ (%)	ND	ND	–	ND	NA
Self-reported positive antibody-test (%)	3 (0.5)	25 (14.3)	–	1 (6.7)	6.68×10^−14^
Self-reported positive PCR-test (%)	6 (1.0)	57 (32.6)	–	0 (0.0)	1.28×10^−33^
Self-reported positive PCR- or antibody-test (%)	9 (1.5)	75 (42.9)	–	1 (6.7)	9.48×10^−45^
SmiNet-case (%)^b^	7 (1.2)	68 (38.9)	–	0 (0.0)	2.66×10^−41^
Days since SmiNet-case	157 (70–272)	74 (10–252)	–	–	0.015
New SmiNet-case^c^ (%)	5 (0.8)	60 (34.3)	–	0 (0.0)	5.73×10^−37^
Vaccinated COVID-19^d^ (%)	3 (0.5)	8 (4.6)	–	12 (80.0)	7.39×10^−21^
Number of vaccine doses					6.08×10^−23^
Not vaccinated (%)	600 (99.5)	167 (95.4)	–	3 (20.0)	
1 vaccine dose only (%)	3 (0.5)	7 (4.00)	–	2 (13.3)	
2 vaccine doses only (%)	0 (0.0)	1 (0.6)	–	10 (66.7)	
3 vaccine doses (%)	0 (0.0)	0 (0.0)	–	0 (0.0)	
Days since vaccine dose 1	8 (4–9)	21 (11–106)	–	45 (11–111)	0.011
Days since vaccine dose 2	–	77	–	13 (6–34)	0.113
Days since vaccine dose 3	–	–	–	–	NA
Hospital SARS-CoV-2 serology test positive^e^ (%)	33 (5.5)	167 (95.4)	–	14 (93.3)	3.76×10^−130^

Phase 3 (*n* = 806)					
*n*	25	32	199	550	-
Female (%)	14 (56.0)	20 (62.5)	130 (65.3)	351 (63.8)	0.820
Age (years)	27 (25.0–27.6)	26.7 (24.9–27.7)	26.5 (24.9–27.8)	26.6 (24.9–27.8)	0.198
Smoking (%)^a^	2 (8.0)	8 (25.0)	19 (9.7)	51 (9.3)	0.063
Symptoms (% since February 2020)	ND	ND	ND	ND	NA
Symptoms (% since August 2020)	ND	ND	ND	ND	NA
Symptoms ≥2 months^a^ (%)	1/10 (10.0)	3/20 (15.0)	26/163 (16.0)	27/207 (13.0)	0.873
Symptoms ≥3 months^a^ (%)	1/10 (10.0)	2/20 (10.0)	21/163 (12.9)	25/207 (12.1)	0.987
Self-reported positive antibody-test (%)	3 (12.0)	19 (59.4)	106 (53.8)	103 (18.8)	7.50×10^−22^
Self-reported positive PCR-test (%)	0 (0.0)	6 (18.8)	97 (49.2)	41 (7.5)	2.06×10^−34^
Self-reported positive PCR- or antibody-test (%)	3 (12.0)	21 (65.6)	156 (78.4)	108 (19.6)	2.03×10^−52^
SmiNet-case (%)^b^	0 (0.0)	9 (28.1)	112 (56.3)	31 (5.7)	4.87×10^−50^
Days since SmiNet-case	NA	257 (11–415)	242 (1–536)	373 (6-621)	1.13×10^−6^
New SmiNet-case^c^ (%)	0 (0.0)	3 (9.4)	66 (33.2)	5 (0.9)	3.44×10^−35^
Vaccinated COVID-19^d^ (%)	1 (4.0)	14 (43.8)	195 (98.0)	541 (98.7)	3.13×10^−49^
Number of vaccine doses					1.70×10^−100^
Not vaccinated (%)	24 (96.0)	18 (56.3)	4 (2.0)	7 (1.3)	
1 vaccine dose only (%)	0 (0.0)	1 (3.1)	8 (4.0)	8 (1.5)	
2 vaccine doses only (%)	1 (4.0)	13 (40.6)	176 (88.4)	516 (94.2)	
3 vaccine doses (%)	0 (0.0)	0 (0.0)	11 (5.5)	17 (3.1)	
Days since vaccine dose 1	123	247 (43–290)	133 (24–401)	121 (12–352)	4.89×10^−5^
Days since vaccine dose 2	83	179 (1–237)	89 (7–251)	78 (4–305)	3.94×10^−6^
Days since vaccine dose 3	-	-	21 (3–62)	21 (1–67)	0.91
Hospital SARS-CoV-2 serology test positive^e^ (%)	ND	ND	ND	ND	NA

Continuous variables were analyzed by the two-sided Mann‒Whitney U test or Kruskal‒Wallis test and are displayed as medians with ranges. Dichotomous variables were analyzed by two-sided Fisher´s exact test and are displayed as numbers and percentages.

^a^Self-reported in Phase 1-3 questionnaires.

^b^SmiNet (a national register for notifiable transmittable diseases in Sweden, covering all positive PCR tests (Public Health Agency of Sweden. SmiNet. In Swedish. Available at: www.folkhalsomyndigheten.se/sminet). A SmiNet case was defined as a positive entry date in SmiNet preceding the date of DBS filter paper sampling in each respective phase.

^c^SmiNet case in the current phase, but not in the preceding phase.

^d^Information on vaccination against COVID-19 was obtained by linkage to the National Vaccination Register. COVID-19 vaccination was defined as receiving the vaccination before the date of DBS filter paper sampling in each respective phase.

^e^Determined from Karolinska University Hospital Laboratory standard SARS-CoV-2 IgG serology test performed at the clinical examination in Phase 2. NA = not applicable, ND = not determined, - = not available.

In Phases 2 and 3, Serocluster 4 had the highest proportion of vaccinated individuals (Phase 2 80%, Phase 3 98.7%). In Phase 3, this serocluster also had the shortest median time between the second vaccine dose and sample collection (78 days). Serocluster 4 contained most of the samples from Phase 3 (Fig. 2e), which is consistent with the vaccination rollout (Fig. 1d). In Phase 3, Serocluster 3 likely constituted a vaccinated group that had been previously infected, based on the high prevalence of self-reported PCR and antibody tests. Similarly, Serocluster 2 in Phase 3 might represent vaccinated and previously infected subjects, for whom vaccinations were given farthest back in time, and there were also fewer new SmiNet cases in this cluster (Table 2). Of all the subjects, only 25 (~3%) remained S– or N– during all the sampling periods, as represented by Serocluster 1 in Phase 3. The majority of these 25 subjects also belonged to Serocluster 1 in Phase 2. The effects of time and possible waning of antibody levels are further described in the supplementary material. In summary, this confirmed the expected seroprevalence rates over time, adding a refined classification from self-sampling for time-resolved immune states.

### Identification of autoantibodies (AAbs) against interferons

**Fig. 3:**
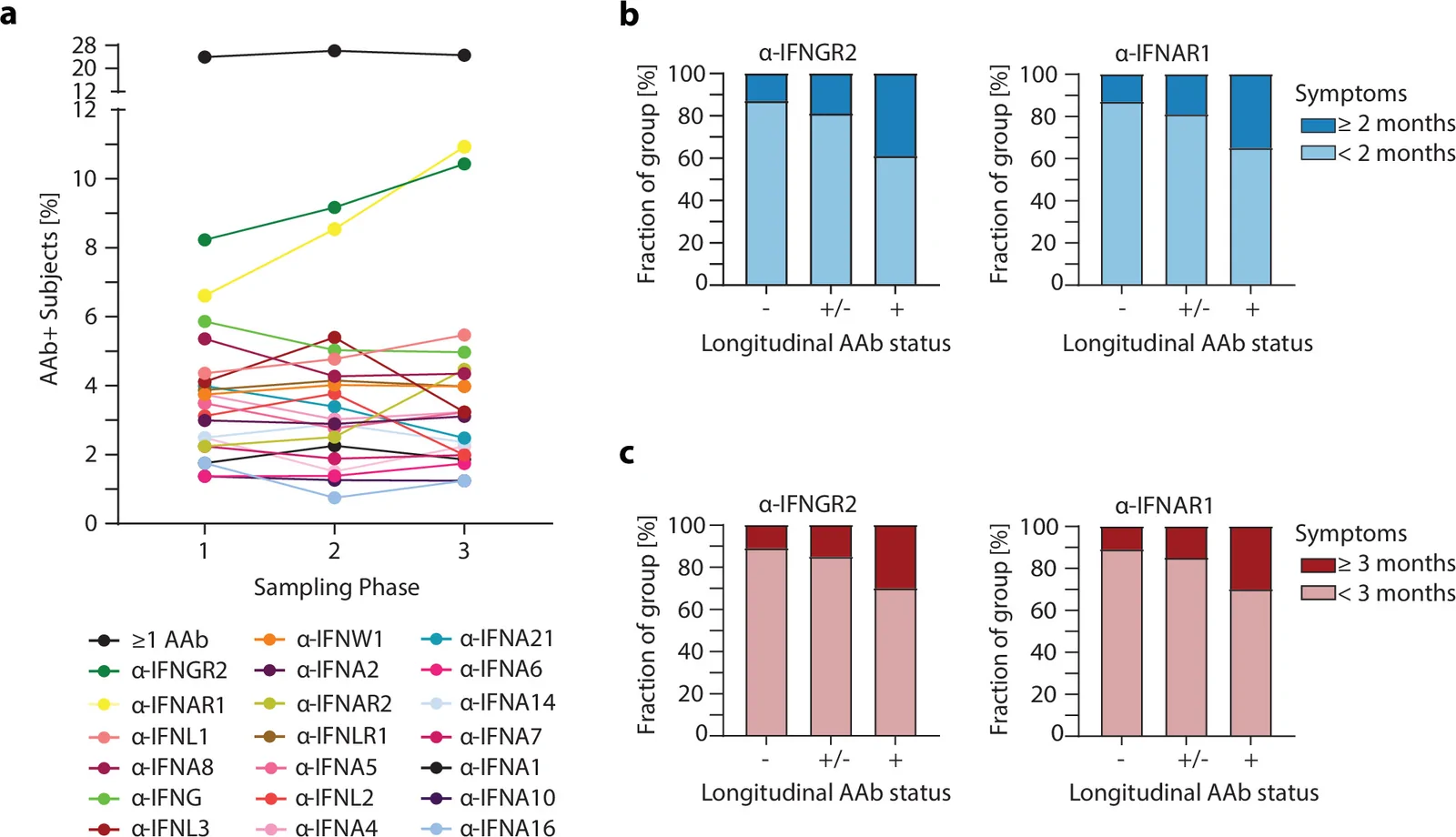
Autoantibody analysis. **a** The proportion of subjects seropositive for ≥1 AAb or each measured AAb within each Phase 1 to 3. **b** The proportions of donors with COVID-19-related symptoms ≥2 months for anti-IFNGR2 (FDR-adjusted *p* = 0.024) and anti-IFNAR1 (*p* = 0.1) and **c** ≥3 months for anti-IFNGR2 (*p* = 0.08) and anti-IFNAR1 (*p* = 0.08) within the groups were always negative (-), always positive (+) and changed from Phase 1 to 3 (+/-) for anti-IFNGR2 and anti-IFNAR1 antibodies, respectively. The data were analyzed by a two-sided Fisher’s exact test and the stacks of the bar plots were colored in light for shorter and dark for longer persisting symptoms.

In addition to measuring pathogen-specific antibodies, we supplemented the bead array with 21 human proteins from the interferon (IFN) family, including 17 ligands and 4 ectodomains from IFN receptors, as previously described^17^. This allowed us to detect autoantibodies (AAbs) in the same measurement and gain further insights into preexisting or newly developed immune states. The proportions of AAb+ subjects within each phase are displayed in Table 3 and Fig. 3a. In this narrow-aged study, individual anti-IFN AAbs were detected at low frequencies, with median prevalence rates of 3.5%, 3.0%, and 3.2% in Phases 1–3, respectively. The highest AAb+ frequency was found against the ectodomains of interferon gamma receptor 2 (IFNGR2; 8.2%, 9.2% and 10.4% in Phases 1–3) and interferon alpha/beta receptor 1 (IFNAR1; 6.6%, 8.5%, and 10.9%), respectively. For most IFNs, the percentage of AAb+ subjects remained stable across phases, except for IFNAR1 and IFNAR2, which increased (Table 3, and Fig. 3a), however these increases were not significant after adjustment for multiple comparisons. There was a strong intraindividual AAb-correlation between the two receptors, IFNAs and IFNLs (Fig. S6). We then divided the subjects based on their longitudinal AAb pattern: seropositive across all phases (AAb+), seronegative across all phases (always AAb*–*), and all remaining and converting patterns across phases (AAb+/*–*); see Table 3. For eight AAbs (IFNGR2, IFNL3, IFNA2, IFNA8, IFNW1, IFNG, IFNAR1 and IFNL1), we observed ≥10 AAb+ subjects. and investigated the following clinical features in relation to AAb-status: sex, BMI (non-overweight vs overweight/obese phase 2), COVID-19 long term symptoms (≥2 months or ≥3 months reported in phase 3), confirmed infection (SmiNet case phase 3), serocluster belonging (phase 3), vaccination status (phase 3), and self-reported current or previous depression (phase 1) Overall, we found that AAbs to IFNL3, IFNA8 and IFNG associated with sex, and that AAbs to IFNAR1 was associated with a higher prevalence or current or previous depression. Further, AAbs to IFNA8 were associated with being overweight/obese and with serocluster belonging, while AAbs to IFNW1 associated with vaccination status. (Supplementary Data 3). Of note, we observed a higher percentage of subjects reporting COVID-19-related symptoms lasting for ≥2 months or ≥3 months within the groups with persistent AAb+ to the ectodomains of IFNGR2 and IFNAR1 (Fig. 3b, c, and Supplementary Data 3), of which the association between AAbs to IFNGR2 and symptoms lasting for ≥2 months was FDR-significant. We further investigated the differences in proportions of long-term symptoms in the AAb groups using a multinominal logistic regression model adjusting for sex, BMI, and serology cluster (Model 1), adding current/previous depression (Model 2), and carriage of a p.F8S low-frequency variant of *IFNAR2* (rs2229207) linked to severe COVID-19^18^ (Model 3). This analysis revealed that being AAb+ for IFNGR2 and IFNAR1 was consistently associated with higher proportions of long-term symptoms (≥2 months and ≥3 months) (Supplementary Data 4).

**Table 3:** Anti-interferon AAb positivity rates per and across sampling phases

	AAb+ Phase 1	AAb+ Phase 2	AAb+ Phase 3	Nominal p value^a^	AAb+ All phases	AAb*–* All phases	AAb + /*–* (converters) All phases
IFNA1	14 (1.8)	18 (2.3)	15 (1.9)	0.74	2 (0.3)	752 (95.2)	36 (4.6)
IFNA2	24 (3.0)	23 (2.9)	25 (3.1)	0.97	10 (1.2)	744 (94.2)	36 (4.6)
IFNA4	30 (3.7)	24 (3.0)	26 (3.2)	0.71	5 (0.6)	736 (93.2)	49 (6.2)
IFNA5	28 (3.5)	22 (2.8)	26 (3.2)	0.70	7 (0.9)	738 (93.4)	45 (5.7)
IFNA6	11 (1.4)	11 (1.4)	14 (1.7)	0.79	4 (0.5)	764 (96.7)	22 (2.8)
IFNA7	18 (2.2)	15 (1.9)	16 (2.0)	0.87	3 (0.4)	753 (95.3)	34 (4.3)
IFNA8	43 (5.4)	34 (4.3)	35 (4.4)	0.51	17 (2.2)	728 (92.2)	45 (5.7)
IFNA10	11 (1.4)	10 (1.3)	10 (1.2)	0.97	1 (0.1)	765 (96.8)	24 (3.0)
IFNA14	20 (2.5)	23 (2.9)	19 (2.4)	0.79	4 (0.5)	744 (94.2)	42 (5.3)
IFNA16	14 (1.8)	6 (0.8)	10 (1.2)	0.20	1 (0.1)	764 (96.7)	25 (3.2)
IFNA17	20 (2.5)	12 (1.5)	18 (2.2)	0.36	3 (0.4)	752 (95.2)	35 (4.4)
IFNA21	32 (4.0)	27 (3.4)	20 (2.5)	0.23	5 (0.6)	733 (92.8)	52 (6.6)
IFNAR1	53 (6.6)	68 (8.5)	88 (10.9)	0.009^b^	46 (5.8)	691 (87.5)	53 (6.7)
IFNAR2	18 (2.2)	20 (2.5)	36 (4.5)	0.025^b^	8 (1.0)	738 (93.4)	44 (5.6)
IFNG	47 (5.9)	40 (5.0)	40 (5.0)	0.67	17 (2.2)	716 (90.6)	57 (7.2)
IFNGR2	66 (8.2)	73 (9.2)	84 (10.4)	0.31	52 (6.6)	684 (86.6)	54 (6.8)
IFNL1	35 (4.4)	38 (4.8)	44 (5.5)	0.58	25 (3.2)	732 (92.7)	33 (4.2)
IFNL2	25 (3.1)	30 (3.8)	16 (2.0)	0.10	6 (0.8)	744 (94.2)	40 (5.1)
IFNL3	33 (4.1)	43 (5.4)	26 (3.2)	0.10	17 (2.2)	733 (92.8)	40 (5.1)
IFNLR1	31 (3.9)	33 (4.2)	32 (4.0)	0.96	8 (1.0)	721 (91.3)	61 (7.7)
IFNW1	30 (3.7)	32 (4.0)	32 (4.0)	0.95	12 (1.5)	732 (92.7)	46 (5.8)

Stated values are counts (% of all).

^a^Fisher´s exact test (two-sided).

^b^FDR *p*-value for IFNAR1 = 0.19, FDR *p*-value for IFNAR2 = 0.20. Phase 1: *n* = 802, Phase 2: *n* = 796, Phase 3: *n* = 805. All phases: *n* = 790.

Longitudinal AAb patterns revealed a low frequency of preexisting and persistent AAbs in a young adult population and highlighted a possible link between anti-IFNGR2 and anti-IFNAR1 AAbs and extended COVID-19-related symptoms.

### Global analysis of the circulating DBS proteins

To expand the molecular phenotype in the same DBS samples, we measured 365 inflammation-related circulating proteins using proximity extension assays (PEA), following protocols and normalization procedures established during previous DBS-based population studies^17,19^. First, we performed principal component analysis (PCA) on all proteins (*n* = 365) in all the samples that passed QC control (*n* = 2394) to evaluate the higher-dimensional data. We observed samples grouping according to time point (Fig. S7a), indicating the overall impact of SARS-CoV-2 exposure on the circulating DBS proteome. Investigating principal component (PC) 1–3 with clinical variables revealed that PC1 was correlated with the sampling phase (Rho = 0.76), sampling date (Rho = 0.75), number of vaccine doses (Rho =  0.75), seroclusters (Rho = 0.71), and levels of anti-S Abs (Rho = 0.65) (Fig. S7c, and Supplementary Data 5). Furthermore, PC1 correlated with the mean protein level in the sample (Rho = 0.95), confirming the impact of preanalytical and clinical factors. Of the 365 proteins, 89 (24.4%) had a PC1 correlation above rho = ± 0.5 (Fig. S7b, d, and Supplementary Data 6). On the other hand, the available clinical variables or design features did not correlate with PC2 or PC3. Among the proteins, IL10 was negatively correlated with PC2 (Rho = −0.73). For PC3, there were 10 proteins with a correlation above Rho < −0.6 and a positive correlation with IL10 (rho = 0.54) (Fig. S7c).

### Associations between circulating DBS proteins and clinical traits

**Fig. 4:**
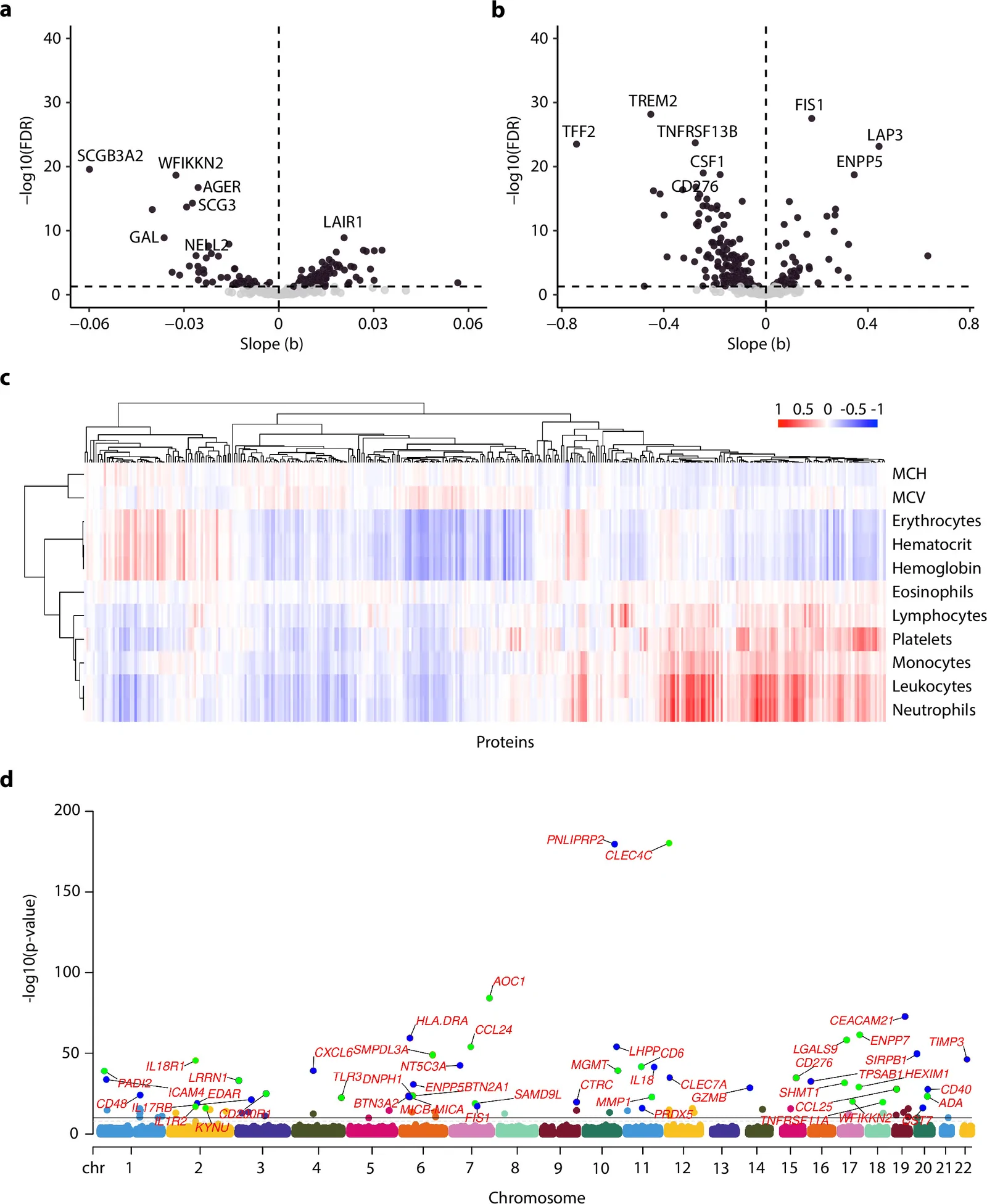
Proteomic and proteogenomic analysis. **a** Volcano plots show the results from linear associations between protein levels and BMI. The beta slopes (b) is plotted against the -log10(FDR) values. The black dots indicate FDR-significant proteins. Some top significant proteins are indicated in the plot. **b** Volcano plots show the results from linear associations between protein levels and sex. **c** The heatmap shows Spearman correlations between CBC and differential parameters and 365 proteins in Phase 2 of the COVID-19 follow-up (*n* = 782). The cell color indicates the degree and direction of Spearman’s rho correlation from −1 in red to +1 in blue. Dendrograms reflect hierarchical clustering using average linkages on distance matrices. **d** The Manhattan plot shows the pQTLs from a genome-wide association analysis (GWAS) of the studied inflammation-related proteins, highlighting the top 50 proteins with the most significant pQTLs. The stated *p*-values were Bonferroni-corrected and obtained by linear regression (PLINK). The chromosome positions were colored arbitrarily.

Based on our initial assessment, we investigated the proteomic associations of demographic factors with age, sex, BMI, and smoking in Phase 2, when a clinical examination follow-up was conducted (Supplementary Data 7). We found that 133 proteins (36%) were significantly associated with BMI (Fig. 4a). Secretoglobin family 3A member 2 (SCGB3A2, FDR = 2.66 × 10^−20^, beta = −0.060), the WAP, follistatin/kazal, immunoglobulin, Kunitz and netrin domain containing 2 (WFIKKN2, FDR = 2.2 × 10^−19^, beta = −0.033), and advanced glycosylation end-product specific receptor (AGER, FDR = 1.8 × 10^−17^, beta = −0.026) were the top-ranked proteins. Compared to previous population-wide studies, the present cohort was more homogenous and narrower in age, ranging from 23.9 to 27.2 years; hence, only five proteins were found to be significantly associated with age. These genes included nuclear distribution C, dynein complex regulator (NUDC, FDR = 0.006, beta =  0.052), Src kinase associated phosphoprotein 2 (SKAP2, FDR = 0.011, beta =  0.087) and phosphoinositide-3-kinase adaptor protein 1 (PIK3AP1, FDR = 0.02, beta = 0.092), which were the top ranked genes. With respect to sex, we found a total of 198 (51%) significantly associated proteins, with peroxiredoxin-5, mitochondrial (PRDX5, FDR = 1.9 × 10^−41^, beta = 0.21), triggering receptor expressed on myeloid cells 2 (TREM2, FDR = 6.9 × 10^−29^, beta = −0.45) and fission, mitochondrial 1 (FIS1, FDR = 3.1 × 10^−28^, beta =  0.18) being the three most significantly associated proteins (Fig. 4b, and Supplementary Data 7, where a positive beta indicates higher protein levels in male participants). We did not find any proteins associated with smoking in our cohort compared to those in the UK Biobank (UKB) cohort, which is likely due to the relatively lower number of smokers (~12% in all three phases) and shorter smoking periods. The results, summarized in Figs .S8 and S9 and Supplementary Data 7, were compared to the beta and *p*-values corrected for multiple testing from a DBS survey^17^ and the large-scale UKB plasma study^1^. For BMI and sex, many protein-trait associations were consistent between the BAMSE and the UKB data (Fig. S8), even though the BAMSE cohort consisted of a narrower age range and a younger population, as well as DBS instead of plasma samples. Associations between protein levels and sex were also concordant between BAMSE and the previous DBS study (Fig. S9)^17^.

### Investigation of circulating DBS proteins for preanalytical factors

To evaluate the impact of time between sampling and analysis for stored DBS samples, we studied the protein levels of 25 persistently seronegative donors (all phases). We identified 99 of 365 (27%) proteins that increased or decreased (Bonferroni *p* < 0.01) with prolonged storage (Fig. S10a, b, and Supplementary Data 8). We then assessed the enrichment of storage-sensitive proteins across protein classes (predicted secreted, intracellular, and/or membrane proteins) from the Human Protein Atlas (Protein Atlas version 25.0) using Fisher’s exact test and found that intracellular proteins were enriched among storage-sensitive proteins (Fig. S10c), suggesting a greater susceptibility to degradation than other classes. This analysis revealed the profound impact of the exposure and sensitivity of blood proteins on preanalytical design aspects.

### Relationship between complete blood cell counts and circulating DBS proteins

To explore how blood cells in DBS samples contribute to the data, we studied the distribution of complete blood cells (CBCs) (Table 4, and Fig. S11) and correlations with DBS protein levels (Supplementary Data 9). Table 4 displays the CBC counts for all 799 subjects with CBC-data from Phase 2, further stratified by seroclusters. Out of 782 subjects with complete CBC and Olink protein data from Phase 2, the majority had CBC counts within the reference ranges provided by the clinical laboratory (Fig. S11)^20,21^. Hierarchical clustering of CBC counts and Olink data from Phase 2 (*n* = 782, Fig. 4c) revealed leukocytes, neutrophils, and platelets as positively correlated (rho > 0.5) with several proteins, including PLAUR, NCF2, OSM, LAT, and CXCL3. Comparatively, erythrocytes, hematocrit, and hemoglobin showed fewer and predominantly negative protein correlations. Leukocytes and neutrophils accounted for most of the FDR-related significant associations (Fig. S12), whereas eosinophils, mean corpuscular hemoglobin (MCH), and mean corpuscular volume (MCV) had relatively few FDR-related significant correlations beyond rho = ±0.5. Figure S13 highlights the strongest significant positive and negative correlations for each CBC parameter.

**Table 4:** Blood cell count and HbA1c measurements per serocluster in Phase 2

Serocluster	All	1	2	4	Nominal *p* value
*n*	799	595	174	15	–
Basophils (range) [´ 10^9^/l]	0.0 (0.0–0.1)	0.0 (0.0–0.1)	0.0 (0.0–0.1)	0.0 (0.0–0.0)	0.99
Eosinophils (range) [´ 10^9^/l]	0.1 (0.0–1.0)	0.1 (0.0–1.0)	0.1 (0.0–0.8)	0.1 (0.0–0.7)	0.18
Leucocytes (range) [´ 10^9^/l]	5.8 (3.0–11.4)	5.8 (3.0–11.4)	5.6 (3.1–9.8)	6.6 (3.7–9.1)	0.20
Lymphocytes (range) [´ 10^9^/l]	1.8 (0.7–3.8)	1.8 (0.7–3.8)	1.9 (0.7–3.6)	1.9 (1.5–2.5)	0.46
Monocytes (range) [´ 10^9^/l]	0.5 (0.2–1.5)	0.5 (0.2–1.0)	0.5 (0.2–1.5)	0.4 (0.3–0.6)	0.61
Neutrophils (range) [´ 10^9^/l]	3.2 (0.9–8.8)	3.2 (0.9–8.8)	2.9 (1.3–7.0)	3.7 (1.7–7.0)	0.05
Erythrocytes (range) [´ 10^12^/l]	4.6 (3.5–6.2)	4.6 (3.6–6.1)	4.6 (3.5–5.8)	4.6 (3.8–6.2)	0.85
EVF (range) [% volume]	0.4 (0.3–0.5)	0.4 (0.3–0.5)	0.4 (0.3–0.5)	0.4 (0.4–0.5)	0.41
Hemoglobin (range) [g/l]	138 (77–179)	138 (77–179)	139 (103–164)	134 (120–174)	0.25
Thrombocytes/platelets (range) [´ 10^9^/l]	253 (88–514)	253 (88–514)	253 (150–453)	287 (163–442)	0.15
MCV (range) [fl]	90 (68–105)	90 (68–105)	90 (81–102)	90 (83–97)	0.09
MCH (range) [pg]	30 (19–35)	30 (19–35)	30 (25–34)	30 (27–32)	0.12
HbA1c (range) [mmol/mol]	31 (20–48)	31 (24–39)	31 (25–41)	32 (27–48)	0.80

The data were analyzed by the Kruskal‒Wallis test and are displayed as medians with ranges. EVF: erythrocyte volume fraction/hematocrit (percent of blood volume). MCV: erythrocyte mean corpuscular volume. MCH: mean corpuscular hemoglobin. Serocluster 3 is not shown due to low subject count (*n* = 3).

Next, we employed linear regression models to investigate associations between DBS proteins and CBC parameters, revealing the largest estimates for hematocrit and the proteins TFF2, FST, and ANGPTL4. The highest number of protein associations was obtained for leukocytes and neutrophils (215 and 212 proteins, respectively) (Supplementary Data 10, and Fig. S14). Similar associations were observed in the adjusted models (for sex alone or sex, smoking status, and BMI; Supplementary Data 11 and 12). Again, the largest estimates were detected between hematocrit and the proteins TFF2, MICB/MICA, and FST, and the highest number of protein associations were detected for leukocytes and neutrophils.

To check the consistency of CBC-protein associations, we evaluated intercorrelations for proteins previously measured in plasma^22^ and now in DBS samples (Fig. S15, and Supplementary Data 13). With 51 overlapping proteins, strong intercorrelations were observed, including between MMP1, IL12B, IL10RB, and CCL4. Finally, we evaluated the CBC~plasma and CBC~DBS protein correlations (Supplementary Fig. 16). We found directionally concordant associations for white blood cell (WBC) parameters (leukocytes and neutrophils), while red blood cell (RBC) parameters (erythrocytes, hematocrit, and hemoglobin) displayed inverse correlations between DBS and plasma samples.

In summary, we determined the associations between dried (DBS) and liquid (plasma) proteins and CBC and found these associations to be most notable for leukocytes and neutrophils. As expected, the presence of RBCs in DBS has a different impact than that in plasma samples^1,23^.

### Baseline genoproteomic architecture of circulating DBS proteins

To map the genetic determinants of the circulating inflammatory proteome while avoiding post-infection or vaccination confounding, we utilized Phase 1 DBS proteomic profiles as a stable baseline reference state (*N* = 650, Wave 1 *n* = 95, Wave 2 *n* = 555). We performed independent genome-wide association testing across 365 inflammation-related proteins in both waves, followed by fixed-effects meta-analysis. To resolve dense local linkage disequilibrium (LD) and isolate distinct causal signals, summary statistics were formatted for conditional and joint multi-SNP analysis (GCTA-COJO) using a 10 Mb window and the Wave 2 cohort as the reference LD panel. Following a multi-protein Bonferroni-adjusted post-selection filter (*P* < 1.3 × 10^–10^), the pipeline successfully isolated a refined set of 664 conditionally independent lead pQTLs acting across 65 distinct proteins.

We resolved a clear partitioning of regulatory mechanisms: 384 (58%) of these associations were local (cis-pQTLs, defined as variants within ±1 Mb of the gene encoding the associated protein), while 280 (42%) acted distantly (trans-pQTLs) (Fig. 4d). Notably, all 384 identified cis-pQTLs were successfully cross-validated against previously reported architectures in the UKB-PPP^1^, SCALLOP^24^, or OpenGWAS^25^ resources. This perfect replication tier aligns with previous large-scale plasma studies and benchmarks the high fidelity of our remote DBS-derived proteomic data for robust genetic mapping. Of the remaining distant signals, 135 trans-pQTLs did not overlap with existing public repositories and represent novel proteogenomic associations.

### Facets of longitudinal changes in circulating DBS proteins

With insights from the impact of exposure, design, clinical traits, blood cell counts, and association with genetics, we evaluated changes across the three consecutive sampling phases. To assess the facets of longitudinal protein variability, intraclass correlation coefficients (ICCs; Supplementary Data 15) and interphase correlations (Supplementary Data 16) were calculated (Fig. S17a, b). Among all 365 proteins, 18 (4.9%) were stable (ICC ≥ 0.75) between Phases 1 ~ 2, 2 ~ 3, and 1 ~ 3, and 14 (3.8%) were stable. Notably, PNLIPRP2, MMP1, CLEC4C, TPSAB1, ICAM4, and CCL24 were among the most stable proteins across all comparisons (ICC > 0.83) (Fig. S17c–h). Reassuringly, all of these had cis-pQTLs (MMP1, CLEC4C, TPSAB1) or trans-pQTLs (PNLIPRP2, ICAM4) at genome-wide significance. A protein with a stable profile between all phases without pQTLs was a Galanin peptide (GAL). Two proteins that had low ICC were CXCL12 and PSPN (Fig. S17i, j).

In addition to protein-level investigations, we studied how the profile of the 800 donors changed over time. When paired-subject correlations were assessed between phases, the mean Spearman correlation was greater for matched pairs (mean rho = 0.25) than for random pairs (mean rho = 0.02) (Fig. S17k). We observed the strongest matched pair correlations between the lower exposure sampling times of Phases 1 ~ 2 and Phases 1 ~ 3, relating to the vaccine rollout and the Omicron wave. However, this approach also applied when limiting persistently seronegative individuals from Serocluster 1 (Fig. S17l), suggesting the possible impact of preanalytical factors.

### Capturing the sampling time-dependent dynamic of circulating DBS proteins

Finally, we investigated the association between pandemic exposure and DBS protein levels over time. To minimize the impact of imbalanced sample sizes, we adjusted the number of donors between groups using random resampling for larger groups. When comparing protein level changes between the I– and I+ subjects, we found leucine aminopeptidase 3 (LAP3) to be consistently elevated in DBS samples close to an infection (median FDR = 4.9 × 10^−5^, median beta = 0.48, iteration variance = 1.7 × 10^−3^, iteration standard deviation = 0.04), see Fig. S18a and Supplementary Data 17. According to cross-cluster comparisons within each phase, LAP3 levels remained significantly elevated in the infected group. After applying bootstrapping for Phase 2 group comparisons, LAP3 remained elevated in all iterations (median FDR = 4.9 × 10^−6^, median beta = 0.33, variance = 7 × 10^−4^, standard deviation = 0.03), see Fig. S18b and Supplementary Data 18. The previously reported COVID-associated protein galectin-9 (LGALS9) was significantly elevated and was selected in 74% of the iterations (median FDR = 0.006, median beta = 0.09, variance = 2 × 10^−4^, standard deviation = 0.01), see Fig. S18b, Supplementary Data 18. No significant differences were observed between the previously infected subjects and the seronegative group (Fig. S19a). When comparing previously infected subjects to more recently infected subjects, LAP3 and LGALS9 were significantly elevated in the newly infected group, while TRAF2 was lower (Fig. S19b, e–g, and Supplementary Data 19).

To further investigate the timing effect of infection and vaccination in relation to DBS self-sampling, we compared longitudinal changes in selected subjects in Phases 2 and 3. We compared 92 seronegative individuals from Phase 2 who were vaccinated and infected in Phase 3 (I–V–converting to I+V+) to 92 infected individuals from Phase 2 who received the vaccine before sampling in Phase 3 (I+V– converting to I+V+). In Phase 2, LAP3 was significantly elevated in the infected group compared with the seronegative group, and in Phase 3, LAP3 was elevated in the more recently infected group (Fig. S19c,h, and Supplementary Data 20). There was also a significant difference in longitudinal LAP3 level changes between these groups, indicating a potential role of LAP3 in the response to infection. There were no significant differences in LAP3 changes when I–V+ individuals were compared with I+V+ individuals showing waning anti-N antibodies (Fig. S19d).

**Fig. 5:**
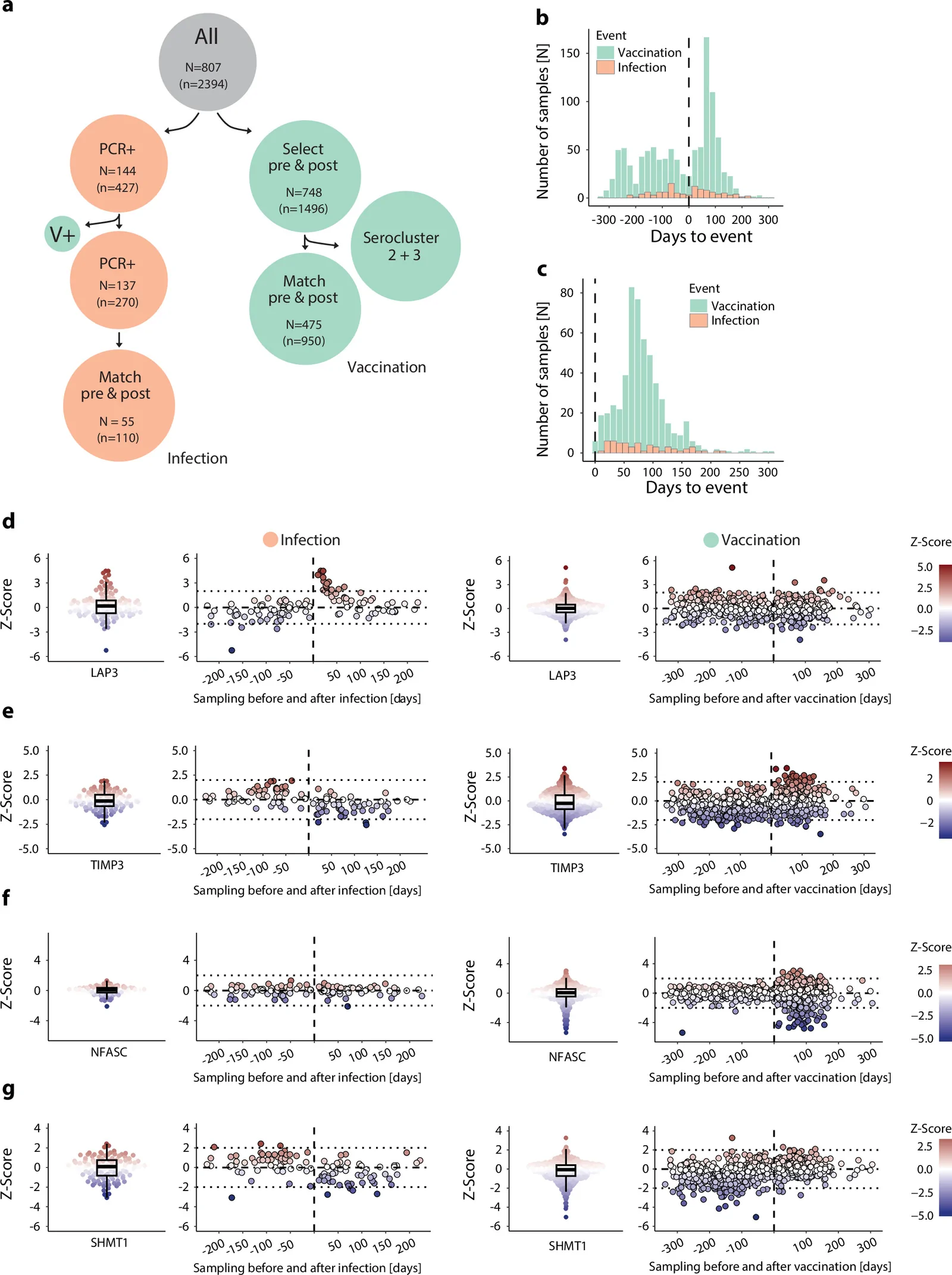
Proteins related to infection and vaccination. **a** Overview of the selection of infected and vaccinated samples. **b** Histogram of time since the event with the closest vaccination dose in green and PCR-confirmed infection in orange for samples pre- and postinfection. **c** Histogram showing only postinfection or postvaccination samples. **d**–**g** Protein *z*-scores plotted against days since PCR-confirmed infection (left panel; *n* = 55) and closest vaccination dose (right panel; *n* = 475). Horizontal dashed lines indicate a *z*-score of 0, the dotted lines mark *z*-scores of ±2, and the *z*-score colors scale from dark purple to dark red. LAP3 had significantly more samples >2 post-infection (FDR = 0.0026, adjusted OR = 0.021). TIMP3 and NFASC had significantly more outliers (above or below ±2 post-vaccination (FDR = 3,59 × 10^−6^, adjusted OR = 0.017 and FDR = 2,028 × 10^−13^, adjusted OR = 0,038, respectively). SHMT1 had significantly more samples with *z*-score <2 pre-vaccination compared to afterwards (FDR = 1.34 × 10^−7^, odds ratio = 12.89). The boxplots show the 25% and 75% quartiles, with the median in the center. Whiskers extend to at most 1.5× IQR from the boxes. For the above, colors indicate infection in teal and vaccination in orange.

Next, we transformed the protein levels into *z*-scores to better compare the number of samples that changed along the time axis of matched pre- and post-infection (*n* = 55) or vaccination samples (*n* = 475); see Fig. 5a–c (Supplementary Data 21). As shown in Fig. 5, several proteins exhibited a significant shift in the number of samples beyond the *z*-score threshold (|*z*| > 2). After infection, significantly more samples had LAP3 *z* > 2 (FDR = 0.0026, adjusted OR = 0.021; Fig. 5d) approximately 1 month after PCR diagnosis (mean 29.5 days, median 27.5 days). Sex-stratified analyzes revealed that LAP3 remained significantly elevated in females (*n* = 39/55; FDR = 0.021; adjusted OR = 0.025). In males (*n* = 16/55) this was not FDR significant. Dissecting the data into a 30-day interval around infection, LAP3 levels were significantly altered between pre-infection baseline and acute post-infection phase among PCR-confirmed patients (paired Wilcoxon: FDR = 0.035, Rosenthal’s *r* = 0.88), see Fig. S18c, d. Following this initial increase, LAP3 levels decreased significantly after the 30-day interval (unpaired Wilcoxon: FDR = 5.97 × 10^−5^, Rosenthal’s *r* = −0.60). Only three subjects had samples collected within 10 days of PCR-confirmed infection, limiting the resolution of further studies of even shorter temporal changes. Only one male provided a sample during this time window, limiting the possibility to conduct sex-stratified analyzes. Notably, LAP3 elevation was not observed when comparing protein levels before and after vaccination (Fig. S18e, and Supplementary Data 21).

Following vaccination, 20 proteins had significantly more samples above the *z*-score threshold (Supplementary Data 21). Among these proteins, TIMP metallopeptidase inhibitor 3 (TIMP3; Fig. 5e), phosphohistidine inorganic pyrophosphate phosphatase (LHPP), and SRSF protein kinase 2 (SRPK2) were the most significant. In contrast, there were significantly more samples for the two proteins above the *z*-score threshold before vaccination. For values below the lower *z*-score threshold, four proteins had significantly more samples after vaccination, with neurofascin (NFASC, Fig. 5f) being the most significant (Supplementary Data 21). Conversely, 54 proteins had significantly more samples below the *z*-score threshold before vaccination. Serine hydroxymethyltransferase 1 (SHMT1; Fig. 5g) was most significantly differentially expressed, with 41 out of 475 samples before vaccination compared to 3 samples after vaccination.

The identification of transient and exposure-dependent multi-molecular alterations in self-sampled DBS adds a new dimension to epidemiological studies, enabling to follow dynamic molecular phenotypes across populations through at-home blood collection.

## Discussion

Comprehensive molecular analyzes of self-sampled DBS elucidated the molecular trajectories of the immune response against natural infection and vaccination in a longitudinal population-based study. This demonstrated the suitability of at-home self-sampling as an accurate alternative to conventional clinical blood sampling for repeated and insightful multimolecular monitoring.

Consistent with previous reports^17^, multiple analyte DBS serology revealed the expected distinction between infections (N+, S+ and RBD+) and vaccinations (N–, S+ and RBD). The IgG data were further backed by PCR-confirmed infection dates and vaccination records from Swedish registries (SmiNet), and repeated sampling improved the resolution, stratification and estimations of antibody decay. By using data-driven seroclustering, we improved the sample stratification according to the actual humoral antibody responses at the time of sampling rather than relying solely on self-reported data.

In addition to anti-SARS-CoV-2 Abs, we detected auto-Abs (AAbs) against several IFN proteins in the same bead-based assay. Despite inconsistencies across different reports of anti-IFN autoimmunity^26^, the longitudinal design enabled us to distinguish between the presence of occasional and consistent anti-IFN AAbs. These findings revealed clinical phenotypes associated with preexisting and persistent AAbs rather than new-onset AAbs, supporting the necessity of considering longer trajectories of autoimmune profiles in health assessment exercises. While most studies investigate AAbs against secreted cytokines, our investigations revealed AAbs against the ectodomains of the IFNG receptors (IFNGR2), and the ectodomain of IFNAR1, which interacts ligands such as with IFNA2, IFNB1, or IFNW1. Like a previous study of DBS self-sampling citizens (general population, aged 18–69, 60% female)^17^, AAbs binding to IFNGR2 and IFNAR1 correlated with each other and were detected in >2% of all samples. Despite this confirmation, the observed anti-IFN receptor AAbs warrant further analyzes (e.g., neutralizing assays). Together with LAP3, however, they are consistent with the established role of interferon signaling as a key mediator of the immune response.

DBS samples offer a minimally invasive sample compatible with high-resolution multimolecular analyzes^5^. However, the impact of cells, such as RBCs or WBCs, on the soluble and secreted components of the plasma proteome^27^ remains unclear because upon drying, the cells leak additional proteins into the DBS. While this approach offers opportunities to identify previously inaccessible proteins, leakage also poses a risk of adding possible confounders to the analyzes. For example, our data suggest that secreted and membrane proteins, possibly due to glycosylation, appear to be more stable during storage than intracellular proteins. Using clinical cell count data, we extracted the missing links between cell count and protein abundance, increasing the impact of how, for example, hematocrit or platelet counts shape protein profiles. This enables a more informed mechanistic interpretation of protein abundance and origin and supports an interchangeable conversion of clinically established and advanced omics measurements.

Many of the protein associations with BMI and sex were consistent in direction and magnitude between the BAMSE DBS data and the UKB plasma data^1^, supporting the utility and validity of the presented data. The lack of common age-associated proteins with UKB plasma data or our previous general population DBS study^17^ is explained by the narrow age range of our participants (±4 years vs. 50+ years). This mismatch also drove inconsistencies in other age-related traits, such as the duration of smoking in years.

Our genome-wide proteogenomic mapping demonstrates that dried blood spot (DBS)-derived proteomic measurements can capture biologically relevant genetic regulation of protein abundance. In line with previous reports on plasma^1,24,28^, we found pQTLs for many targets, including MMP1, PNLIPRP2, and TPSAB1. This finding supports the notion that we observed the intended targets in our DBS assays, as recently suggested in a study on newborns^23^. Mapping the genetic regulation of the inflammatory proteome has traditionally been challenging due to dynamic baseline variations and low physiological abundance. Hence, these proteins are understudied and require larger sample sizes or meta-analyzes^29^. Here, our diverse cis-pQTL signatures included key immune and tissue-specific mediators, such as the lymphoid cell surface receptor CD200R1, the circulating interleukins IL12B and IL18, the dendritic MHC class II DR alpha (HLA-DRA), the placental growth factor PGF, and the macrophage immune receptor TREM2 – demonstrating the systemic breadth captured by the DBS proteome.

Furthermore, cross-referencing our data with eQTLGen whole-blood consortium databse provided supportive functional annotation for 54% of the 135 novel trans-pQTLs. Among these, Tier 1 (validated blood regulator) was strongly associated with the cis-expression of their nearest regulatory gene (e.g., CCDC77, ABHD5, AGK), suggesting upstream transcription-mediated regulatory mechanisms. In contrast, Tier 2 (Direct Target eQTL) hits (e.g., MGMT, TNFRSF11A, and LGALS9) revealed direct eQTL effects on the distal protein-encoding target genes themselves, implying possible long-range chromatin interactions, although functional validation will be required. Because proteogenomic biobanks (such as UKB-PPP and SCALLOP) rely exclusively on cell-free plasma or serum, the relative scarcity of DBS- and whole-blood-based pQTL studies may partly explain why several trans-pQTLs identified here have not been reported previously. DBS may provide complementary information to plasma-based studies by capturing both circulating and cell-associated protein components.

Our study highlighted the temporal increase in LAP3 levels in DBS proteomes from recently infected subjects. As an aminopeptidase involved in immune regulation, LAP3 has previously been reported to increase following SARS-CoV-2 infection in DBS^17^ and plasma^10^, as well as in other viral infections, such as generalized lymphadenitis and adenovirus infection^30^. Cellular studies confirmed that LAP3 increased 3 days after infection with SARS-CoV-2-infected cells and was classified as an interferon-induced protein^31^. This, together with the AAbs against IFN receptors, provides a functional coupling of the presented data with IFN signaling. These findings suggest that LAP3 is not exclusively specific to SARS-CoV-2 infection but has a broader role in the human response to various viral or bacterial pathogens, as shown in a recent pan-disease study^7^. This transient decrease in cross-sectional levels post-infection is unique to this study, as it occurred in all donor samples collected within 3–6 weeks post confirmed infection. Compared to other immune markers with a more rapid decline, such as CCL2 or CXCL9^32^, the gradual decrease in LAP3 DBS levels made it possible for us to capture these in a study with an exposure-unrelated (random) sampling event in a generally healthy young population. It is therefore reasonable to assume that, without an enrichment of severe cases, other immune markers with previous links to SARS-CoV-2 infections were missed due to a steeper post-infection decline. To complement the knowledge on latencies reported for IgG or IgM, studies with tighter sampling events are needed.

Our study included mainly inflammation-related proteins; hence, other important pathways or pathologies, such as coagulation or metabolic phenotypes, may have been missed. Including additional proteins in targeted or discovery-driven analyzes in future studies could help researchers identify other, likely more persistent, proteomic changes following infection. In clinical reports enriched for severe phenotypes, consistent elevation of immune proteins over a longer period has been linked to adverse outcomes^33^. However, in cross-sectional population surveys, the heterogeneity of disease states, impact of age-accumulated comorbidities and spread of sampling timepoints influence our ability to draw simpler conclusions. Other studies have used a model system and near-daily DBS sampling to show that the levels of many inflammation-related proteins return to baseline levels within 2 weeks post infection^32^. Here, nonhospitalized young adults (aged <30) from the BAMSE cohort were included; they were neither instructed to change their behavior during the study nor told to change their self-sampling plans due to an infection or current symptoms.

The characteristics of our study population also influenced and limited some of the outcomes. While the inclusion of informed participants representing a generally healthy, younger segment of the population is an interesting aspect, it also poses challenges for detecting large variance in protein levels in a rather homogeneous group. The cohort largely followed national recommendations at the time, and most individuals received vaccination as soon as it became available for their age group in Sweden. This resulted in overlapping seroconversion periods and limited the formation of sufficiently large, clearly separated comparison groups. The coinciding vaccination campaign, the start of the third (and final) sampling phase, and the emergence of the fast-spreading Omicron variant presented challenges for disentangling the longitudinal differences from storage effects across the three collection phases. Therefore, fully distinguishing these interconnected events and assigning specific biological effects to each event are challenging.

Longitudinal population-based DBS home sampling captured robust insights into the dynamic multi-molecular events occurring during three pandemic years. Together with cohort data on genetic variation, clinical diagnostics and health history, monitoring health at multiple molecular levels in self-sampled DBS specimens highlighted the added value of time-resolved phenotyping. This will aid epidemiological studies and those aiming to stratify individuals at risk, where preexisting and persistent but also shorter-term alterations could influence long-term health outcomes.

## Methods

### Study population and sampling

The study population included 808 participants from the Swedish Barn [Children], Allergy, Milieu, Stockholm Epidemiology (BAMSE) birth cohort. This was originally a population-based prospective cohort consisting of 4089 children born between 1994 and 1996 in Stockholm, Sweden. The participants were followed up with repeated clinical examinations and questionnaires. During the pandemic, the participants included in the present study were invited to participate in a COVID-19 follow-up divided into four phases^34^. The current study included data from the first three phases: Phase 1, from August to November 2020; Phase 2, from October 2020 to June 2021; and Phase 3, from October 2021 to May 2022. We used web-based questionnaires and self-collected quantitative dried blood spot (DBS, Capitainer AB) samples from finger pricking at all three phases. Furthermore, a clinical examination was conducted at the Stockholm South General Hospital during Phase 2^35,36^. The DBS samples were self-sampled at home in Phase 1 and 3 or self-sampled at the clinical examination in Phase 2, assisted by a nurse. The home-collected samples were mailed back using regular mail, and all DBS samples were stored at room temperature until analysis. Informed written consent was obtained from all subjects, and ethical approval was granted by the Regional Ethical Review Board in Stockholm (DNR 2016/1380-31/2) and the Swedish Ethical Review Authority (DNRs 2020-02922, 2024-06052-02)^34^.

### Questionnaire, clinical and registry variables

The data on current smoking status were self-reported in Phase 1 to 3 questionnaires. The participants self-reported symptoms of suspected or confirmed COVID-19 in each phase, with the Phase 1 questionnaire covering the start of the pandemic through the date of Questionnaire 1, with Phase 2 covering August 2020 until the date of Questionnaire 2, and with the Phase 3 questionnaire covering the entire pandemic until the date of Questionnaire 3. Among the subjects who reported symptoms, follow-up questions regarding symptom duration were used to define long-term symptoms that extended over 2 months and 3 months. From the questionnaires, we also extracted self-reported data on positive results on COVID-19 PCR or antibody tests. At the clinical examination of Phase 2, body mass index (BMI) and body fat percentage were measured using a bioelectrical impedance machine (Tanita MC 780 body composition monitor). Additional SARS-CoV-2 IgG serology in plasma collected during Phase 2 clinical examination was obtained from the Karolinska University Hospital Laboratory standard SARS-CoV-2 IgG serology test. Information about confirmed infection was obtained from SmiNet, which is a national register for notifiable transmissible diseases in Sweden that covers all registered positive PCR tests (Public Health Agency of Sweden, SmiNet, in Swedish. Available at: https://www.folkhalsomyndighetense/sminet). A SmiNet case was defined as a positive entry date in SmiNet preceding the date of DBS filter paper sampling within each respective phase. Information on vaccination against COVID-19 was obtained by linkage to the obligatory National Vaccination Register. COVID-19 vaccination was defined as receiving a vaccination dose before the date of DBS filter paper sampling in each respective phase.

### DBS sample preparation

After visual inspection of each sample card to determine if at least one of the paper discs on the sampling device was correctly filled with blood, we randomly transferred one disc per subject and timepoint into flat-bottom 96-well plates (VWR, art no. 734-2327) using a semiautomated puncher (710-1100, PH96, Preanalytical Handler 96 for Capitainer®, Intema). All samples (Phases 1, 2 and 3) from the same participant were transferred to the same plate, and each plate was filled with 84 discs. We then added 100 µl of buffer (1× phosphate-buffered saline (PBS, Medicago) with 0.05% Tween 20 and protease inhibitor cocktail (#04693116001; Roche) to each well, and the discs were incubated under gentle rotational shaking (170 rpm) for 60 min at room temperature. Following centrifugation, all the proteins were transferred to a PCR plate (#732-4828, VWR) and were subsequently centrifuged for 3 min at 2100 × *g* (Allegra X-12R, Beckman Coulter, Inc.). From this plate, we collected 70 µl of supernatant into a new PCR plate, and the eluates were stored at –80 °C prior to analysis.

### Detection of anti-SARS-CoV-2 antibodies in DBS

The SARS-CoV-2-related recombinant proteins and antibodies used as controls, either obtained from commercial providers or produced as described previously^37,38^, were covalently coupled to color-coded magnetic microspheres (MagPlex, Luminex Corp.) using NHS/EDC chemistry as described elsewhere^14,17,39^. The antigens and antibodies used are listed in Supplementary Data 22. To summarize, 4 µg of protein or 1.7 µg of antibody was diluted in MES buffer [100 nM 2-(N-morpholino)ethanesulfonic acid (pH 5.0)] to a final volume of 100 µl. The carboxylated surfaces of the beads were then activated by incubating the beads with 50 µl of activation solution (N-hydroxysuccinimide (Pierce) and 1-ethyl-3-(3-dimethylaminopropyl)-carbodiimide (ProteoChem)) for 20 min. After washing with MES buffer, we added the diluted proteins to the beads and incubated them for 2 h at 23 °C. The beads were then washed with 0.05% PBS-T and stored in blocking buffer for ELISA (BRE, 11112589001, Roche) overnight before we pooled beads of different bead identities to form a suspension bead array (SBA).

On the day of the assay, the blood eluates were thawed at 4 °C and diluted 1:2.5 in assay buffer containing 1× PBS with 0.05% Tween 20, 3% BSA (B2000-500, Lot# 04E6171, Saveen Werner) and 5% milk powder (70166-500G, Lot# 102586039, Sigma‒Aldrich). We then transferred 35 µl of the diluted eluates to 384-well plates containing 5 µl of antigen SBA and incubated the plates in the dark for 60 min at 23 °C with shaking at 650 rpm. Subsequently, the plates were washed three times with 60 µl of 0.05% PBS-T using an automated washing system (BioTek EL406), and 50 µl of anti-human IgG-R-PE (H10104, Lot#2306068, Invitrogen) diluted to 0.4 µg/ml in 0.05% PBS-T was added to the beads. The beads were then incubated for 30 min at 23 °C with shaking at 650 rpm in the dark. After three final washes with 0.05% PBS-T, 60 µl of 0.05% PBS-T was added to the beads, and the fluorescence associated with each bead was measured in a FlexMap 3D instrument (Luminex Corp.). The data are reported as the median fluorescence intensity (MFI) per antigen and sample, where each data point has a bead count of at least 32 per ID.

### Autoreactive antibody assays in DBS

In addition to the COVID-19 antigens, 21 human interferons (IFNs) were included in the bead mixture of the SBA. IFNs were produced as described elsewhere^40^. The protein coupling, analysis and readout were performed as described for the detection of the SARS-CoV-2 antibodies described above.

### Affinity proteomics assays in DBS

To measure relative protein levels in the samples, the samples were analyzed using the Olink Explore 384 Inflammation Panel (Lot # B23405, Olink) at the SciLifeLab Affinity Proteomics Unit in Uppsala, Sweden. In brief, 1 µl of eluate was incubated with antibodies labeled with unique DNA oligonucleotides. If two matched antibodies simultaneously bind to a target protein, their DNA tags can hybridize, and polymerase-dependent extension is used to create a double-stranded DNA ‘barcode’ unique for the specific antigen and to amplify the signal. The resulting amplicon was then quantified via next-generation sequencing (NGS) on an Illumina instrument, and the reported Ct values were converted to normalized protein expression (NPX) values using Olink NPX Explore software (version 3.0.5) and intensity normalization. The NPX values are presented as arbitrary units (AUs) on the log2 scale. In total, 365 inflammation-associated proteins were detected in 2398 of the samples.

### Complete blood cell counts

Complete blood counts (CBCs) were assessed at the BAMSE COVID-19 follow-up in Phase 2 and at the BAMSE 24-year follow-up using whole blood collected via venipuncture. The 24-year follow-up took place between November 2016 and May 2019, approximately 1–4 years before Phase 1 of the COVID-19 follow-up (starting in August 2020). Blood analysis was conducted by routine flow cytometry at Karolinska University Laboratory in Stockholm, Sweden, with reference values provided by the same laboratory^20,21^. Figure S11 presents the distributions of CBC parameters at Phase 2 based on data from 782 subjects with complete CBC and Olink protein data from Phase 2 of the BAMSE COVID-19 follow-up. Given the pronounced skewness of the basophil count toward zero, this parameter was excluded from downstream analyzes.

### Affinity proteomics assays in plasma

At the BAMSE 24-year follow-up, the relative levels of 92 inflammation-related plasma proteins were assessed using the Olink Target 96 Inflammation Panel (Olink, Uppsala, Sweden). Plasma was obtained from venous blood collected in EDTA tubes. The Olink Target 96 method is similar to Olink Explore 384, except for the use of quantitative real-time polymerase chain reaction instead of next-generation sequencing. Samples that deviated more than 0.3 NPX from the median value of an internal control were excluded.

### Statistical analyses

All the data analyzes and visualizations were performed using the R (version 4.4.1) programming language^41^ or STATA SE 19.5 unless otherwise stated. Tidyverse (version R 2.0.0)^42^ was used for data processing and analysis, and plots were created using the ggplot2 package (version 3.5.1)^43^ together with patchwork (version 1.3.0)^44^, RColorBrewer (version 1.1-3)^45^, ggh4x (version 0.3.1)^46^, ggbeeswarm (version 0.7.2)^47^, ggrepel (version 0.9.5)^48^, ggsignif (version 0.6.4)^49^, GGally (version 2.4.0)^50^, ComplexHeatmap (version 2.18.0)^51^, viridis (version 0.6.5)^52^, Ghibli (version 0.3.4)^53^, and ggalluvial (version 0.12.5)^54^. The psych package (version 2.4.6.26)^55^ was used for calculating Cohen’s kappa and intraclass correlations (ICCs). Multiple testing was accounted for using Benjamini–Hochberg FDR, unless otherwise stated in the methods. FDR p-values are displayed throughout the results text, and nominal *p*-values are further displayed in the Tables. The statistical analyzes employed in this study are summarized in Supplementary Data 23.

### Demographic data analysis

The demographic data are displayed in Tables 1 and 2. Fisher’s exact test was used to analyze dichotomous variables, which are displayed as the number of subjects and percentage, and the Mann‒Whitney U test or Kruskal‒Wallis test was used to analyze continuous variables, which are displayed as medians with ranges.

### Serological data analysis

SARS-CoV-2 serological assay data were normalized using a mixed model implemented in Julia (version 1.7.2)^56^ programming language to remove correlations with negative control beads for all analytes, except for spike- and RBD-based versions, which were normalized against an anti-human IgM bead to account for rising antibody levels postvaccination. The data from all sampling phases were normalized together for all analytes except for the interferons, which were normalized per sampling phase. The model, applied to log2-transformed data as previously described^14,17^, incorporated both linear and uniform distribution components to account for distributions where most samples exhibited low baseline levels, while a subset displayed higher positive values. The resulting relative log2 values were scaled and centered on the same scale for each protein.

Seropositivity for each anti-SARS-CoV-2 antibody was determined using a population-based threshold of 6 standard deviations (SD) of the estimated negative proportion above the Gaussian population peak as previously described^14^. The negative population consisted of unvaccinated individuals without PCR-confirmed infection. This threshold, established using data from sampling Phases 1 and 2, was applied to Phase 3. Phase 3 was excluded from threshold determination due to the high proportion of vaccinated individuals in this phase. To determine the number of subjects who remained seronegative across all three sampling phases, the serostatus was first determined at the sample level. For each sample, the antigen-specific seropositivity was summarized separately for N, S, and the RBD by applying a majority rule across all corresponding antigens within each group. A sample was then classified as seropositive if at least two of the three antigen groups (N, S, and RBD) were seropositive. Participants were considered seronegative across all phases if all samples collected from that subject were classified as seronegative.

Cohen’s kappa coefficient was calculated using the psych R package (version 2.4.6.26)^55^ to quantify the level of agreement between the seropositivity estimated in this study and the seropositivity estimated using ELISAs targeting the spike protein in plasma samples from Phase 2.

Hierarchical clustering using the maximum distance and Ward. The D2 agglomeration method was applied to six binders (full-length N, NC, S1, S-WT, RBD-WT, and RBD-Omicron) to stratify the samples into four serocluster groups based on serological profiles. The optimal cluster number was determined by evaluating the elbow point of the gap statistic curve and dendrogram interpretability. Cluster stability was validated via bootstrap resampling using the mean Jaccard index. These seroclusters and the seropositivity for each antibody were subsequently compared to the available vaccination and infection status data.

For the determination of seropositivity against interferons, signals were transformed using robust *z*-scores per phase, and a cutoff of 3.5 was applied to assign seropositivity per phase. We divided the subjects based on their longitudinal AAb pattern: AAb+ (seropositive across all three phases), AAb*–* (seronegative across all three phases) and AAb+/*–* (converters) (all remaining patterns across three phases (Table 3). For eight AAbs (IFNGR2, IFNL3, IFNA2, IFNA8, IFNW1, IFNG, IFNAR1 and IFNL1), we observed ≥10 AAb+ subjects and these AAbs were included in downstream analyzes (Table 3). Differences in proportions of sex, BMI, long-term COVID-19 symptoms, current or previous depression, confirmed SARS-CoV-2 infection and SARS-CoV-2 vaccination and SARS-CoV-2 serology cluster belonging to the AAb+, AAb– and AAb+/– (converters) groups were analyzed by Fisher´s exact test. We further investigated the differences in proportions of long-term symptoms in the AAb groups using a multinomial logistic regression model adjusted for sex, BMI and serology cluster (Model 1) and adding self-reported current/previous depression (Model 2) and carriage of a p.F8S low-frequency variant of *IFNAR2* (rs2229207) linked to severe COVID-19 (Model 3), with the AAb– group as the reference. In our study population, the frequency of carrying the gene variant rs2229207 was 17.12%.

To evaluate the relationship between AAbs across different study phases, pairwise correlation analyzes were performed. For each phase, Pearson correlation coefficients were calculated using the binary robust *z*-scores. The resulting correlation matrices were visualized using hierarchical clustering heatmaps generated via the heatmap.2 function from the gplots package.

### Proteomic data analysis

Intensity-normalized protein measurements and plate control-normalized protein measurements were obtained from Olink. In general, we used intensity-normalized data for downstream analysis since this normalization method yields more stable NPX values for proteins not highly expressed in the Olink-specific plate controls.

To minimize sample-to-sample variation due to sample handling and nonspecific protein correlation, the data were normalized using the ProtPQN method with the ProtPQN R package (version 1.0.1)^57^. Only samples from the BAMSE cohort were included in the ProtPQN normalization.

Linear models were generated using the stats base R package (4.4.1)^41^. For protein associations between age, sex, BMI, and smoking status, we fitted linear models for all samples from Phase 2 for each of the proteins against each of the other variables. *P* values were adjusted using FDR correction and were, together with the slopes, compared to the slopes and Bonferroni-adjusted *p* values from the UK Biobank (UKB) data^1^ and slopes and FDR-corrected *p*-values from previous DBS data^17^ for the overlapping proteins and variables. For the BAMSE data, we used a FDR value < 0.05 as the significance threshold, whereas for the UKB dataset, we used Bonferroni *p* < 1.7e-5.

To assess protein stability over time, we calculated ICCs for each protein using the ICC() function of the R psych^55^ package. Of the various ICC forms available, we specifically chose the ICC(3,1) model. We opted for ICC(3,1) because it represents a two-way mixed-effects model for absolute agreement of single measures. Proteins were deemed stable if their calculated ICC(3,1) was ≥0.75, based on suggested guidelines for the interpretation of ICC values^58^.

Linear mixed-effect models (LMMs) were generated using the *lmerTest* R package (version 3.1-3)^59^. LMMs were applied to evaluate associations between protein levels and storage time among uninfected and unvaccinated individuals across all three sampling phases, adjusting for sex and including a random intercept for each subject. This analysis was performed on 25 subjects who consistently remained in Serocluster 1 across all three sampling phases, minimizing the potential influence of infection or vaccination on protein levels. Bonferroni correction was used to account for multiple hypothesis tests, and a significance threshold of 0.01 was used. We then assessed the enrichment of storage-sensitive proteins across protein classes (predicted secreted, intracellular, and/or membrane proteins) from the Human Protein Atlas using Fisher’s exact test.

To compare protein levels between subgroups of the data to investigate the effect of infection and vaccination, the data were first adjusted for storage time by taking the residuals from an LMM between protein levels and storage time with a random slope per subject. Using the data adjusted for storage time, we first compared naïve seronegatives (Serocluster 1) who were infected prior to Phase 2 (Serocluster 2; *n* = 96) to a randomly sampled group of naïve individuals who remained naïve (*n* = 96), bootstrapped over 100 iterations and to infected individuals who remained infected (Serocluster 2, *n* = 77). We also compared the randomly sampled groups of naïve individuals who remained naïve to the groups of subjects who remained infected. Differences in protein levels between the groups within each sampling phase, as well as the median changes over time between the groups, were assessed using linear models adjusted for sex via the lm function of the *stats* R package. When comparing two groups of unequal sample sizes, the same number of subjects as in the smaller group were bootstrapped from the larger group with replacement for 100 iterations, and the statistical comparison was performed in each iteration. The results from the bootstrapped analyzes were summarized using the median effect size and corresponding estimates across all iterations.

Protein levels were evaluated using Wilcoxon tests implemented via the wilcox.test function from the *stats* R package. In the primary analysis of unvaccinated, PCR-confirmed infected individuals, baseline pre-infection samples (*n* = 55 subjects/samples) were compared against post-infection samples divided into two post-infection intervals (0–30 days, *n* = 12; >30 days, *n* = 43) to capture potential nonlinear temporal changes. Paired Wilcoxon signed-rank tests were applied to compare the pre-infection baseline against the two post-infection groups, while an unpaired Wilcoxon rank-sum test was used to compare the two post-infection intervals against each other due to non-overlapping subject comparison. In a secondary analysis, individuals who were vaccinated prior to PCR-confirmed infection were also included, and unpaired Wilcoxon rank-sum test was used to compare pre-infection samples (*n* = 203 samples from 133 unique subjects) against post-infection samples divided into the two time intervals (0–30 days, *n* = 23; >30 days, *n* = 201 samples from 134 subjects). As a control analysis, individuals who were vaccinated but had no evidence of infection were included; therefore, we excluded samples from Serocluster 2 and 3 since these clusters contained infected individuals with antibodies against nucleocapsid proteins. For each subject, the most recent sample obtained before vaccination and the most recent sample obtained after vaccination were compared using paired Wilcoxon signed-rank tests. Comparisons between the two post-vaccination time intervals were performed using an unpaired Wilcoxon rank-sum test because these consisted of different subjects. For all testing configurations, standardized non-parametric effect sizes were calculated as Rosenthal’s *r* ($r=\,\frac{Z}{\surd N}$) using the wilcox_effsize function from the *rstatix* package, where *Z* represents the standardized test statistic and *N* represents the total number of observations. To preserve biological directionality, a positive or negative sign was assigned to each effect size matching the direction of the median expression change between compared groups.

To plot protein levels in relation to the time since infection, all protein levels were first transformed into *z*-scores. Then, all samples from subjects with a positive PCR test were extracted. Samples collected after vaccination were then excluded, and the remaining samples were matched to have one pre- and one post-infection sample per subject. In total, 110 samples from 55 subjects were included. The date of PCR-confirmed infection was used as the infection date, acknowledging that this does not perfectly correspond to the true date of infection due to the delay between infection and testing. To plot protein levels in relation to the time since vaccination, matched pre- and post-vaccination samples closest in time to the vaccination date were selected. Samples from subjects belonging to infected seroclusters 2 or 3 were excluded. In total, 950 samples from 475 subjects were included.

Fisher’s exact test was used to compare the proportion of samples with extreme protein levels (*z*-score > |2|) between the pre- and post-sampling timepoints within the infected and vaccinated groups described above. Tests were performed in R using the fisher.test function from the *stats* package. To account for zero-cell count in the contingency tables, a Haldane–Anscombe correction was applied by adding 0.5 to every cell prior to calculating adjusted odds ratios.

### Genome-wide association of proteomics data

Because germline genetic variation is fixed over time, Phase 1 proteomic measurements were used as the reference proteomic state for baseline pQTL discovery analyzes. This approach captures stable genetic regulation of the proteome and avoids potential temporal confounding or exposure confounding (SARS-CoV-2 infection, vaccination or other pandemic-related aspects). To characterize the genetic architecture of protein levels detected in DBS, we performed a protein quantitative trait locus (pQTL) association analyzes using Phase 1 Olink measurements. The genotyping and imputation protocols for the BAMSE samples have been previously described^60^. Briefly, genotyping of the full cohort (n≅2892) was performed in two distinct waves separated by approximately 10 years utilizing different Illumina platforms (Illumina Human 610-quad BeadChip and Illumina Infinium Global Screening Array-24 v1.0 (GSA) chips). Standard quality control measures were applied independently to the assays in both waves for genotype and sample quality control, and the imputation quality control (QC) measures were anchored to the HRC reference panel.

Of the subjects with Olink proteomic data, we successfully matched 678 samples to the imputed data. After QC for available covariates, which resulted in the exclusion of 28 samples, the final analysis set included 650 samples. The ProtPQN intensity-normalized NPX values for each Olink protein from sampling Phase 1 were inverse rank normalized and adjusted for sex, age, and the top 20 genetic principal components (PCs). To increase the total cohort size, a Genome-Wide Association Study (GWAS) was first performed independently for each of the 365 Olink-measured inflammation-related DBS proteins in Wave1 (*n* = 95 samples) and Wave2 (*n* = 555 samples) using Plink 2.0 on chromosomes 1–22 on all biallelic SNPs filtered at a MAF > 0.01 and imputation quality > 0.3 in each wave. The summary statistics from both waves were subsequently combined via a fixed-effects inverse variance-weighted (IVW method) meta-analysis implemented in the METAL software^61^ to generate the final pQTL estimates. To isolate conditionally independent association signals within each protein locus, conditional and joint association analysis was executed via GCTA-COJO (--cojo-slct). The stepwise selection model utilized an internal genome-wide significance threshold of *P* < 5 × 10^−8^ across a generous 10 Mb genomic window (--cojo-win 10000), with the clean imputed Wave2 cohort serving as the in-sample LD reference panel. To rigorously account for the multi-phenotype testing burden across all measured proteins, we applied a post-COJO study-wide Bonferroni significance filter, retaining only independent lead pQTL signals that maintained a joint/conditional *P* < 1 × 10^−10^. Consistent with large-scale proteogenomic consortium standards (e.g., UKB-PPP, SCALLOP), cis- and trans-pQTL designations were defined strictly by the genomic coordinates of the variants relative to the protein-encoding gene locus (±1 Mb)^62^, independent of the cellular or extracellular destination of the mature protein product. We subsequently checked for overlap with previously reported pQTLs among European populations in the UKB-PPP consortium data^1^, SCALLOP^29^, OpenGWAS^63^, and GPPAD newborns^23^. The significant results from the eQTLGen 2019-12-11 release were used to annotate the novel trans-pQTLs.

### Analyses of blood cell counts and circulating DBS proteins

We first applied Spearman correlation to study associations between DBS Olink proteins and CBC parameters in 782 participants with complete protein and CBC data from Phase 2 of the COVID-19 follow-up. Hierarchical clustering was performed on both CBC parameters and DBS proteins using the “average” method on a distance matrix derived from the Spearman correlation coefficients.

Second, we examined the correlations between 51 proteins measured in both DBS (COVID-19 follow-up) and plasma samples (24-year follow-up). The analysis included 690 subjects from the 24-year follow-up with complete CBC and protein data at both time points; 782 subjects were included from Phase 2. Finally, we examined the relationship between CBC parameters and plasma proteins at the 24-year follow-up by employing Spearman correlation and compared these results to the corresponding correlations for the same 51 proteins measured in DBS samples. Shared protein-level associations across CBC parameters were evaluated via hierarchical clustering (average linkage) using correlation-based distance metrics. All analyzes were performed in R version 4.4.1 (2024-06-14)^41^, and visualizations were created using the R packages ggplot2^43^, grid^41^, gridExtra^64^, pheatmap^25^, rcartocolor^65^, and UpsetR^66^.

### Reporting summary

Further information on research design is available in the Nature Portfolio Reporting Summary linked to this article.

## Supplementary information

Supplementary Information

Description of Additional Supplementary Files

Supplementary Data 1-23

Reporting Summary

Transparent Peer Review file

## Source data

Source Data

## Data Availability

Individual-level data underlying this study cannot be deposited openly due to consent and legal restrictions, but can be requested from the corresponding author for non-commercial research or validation purposes. Requests must specify the requesting PI and institution, contact details, intended scientific use, and include commitments to report resulting publications, not redistribute the data outside the requesting organization, and use the data non-commercially. Requests will be answered within four weeks, except where new ethical approval is required, and access will be granted for an agreed timeframe. Source data are provided with this paper.

## Code availability

All code necessary for the data analyses and visualization is available at https://github.com/Schwenk-Lab/^67^. The code for the pQTL analysis is available at https://github.com/kumar-ashish/BAMSE-pQTL-analysis/^68^.
