# Supplementary material for: Molecular profiling of repeated self-sampled blood reveals dynamic immune phenotypes in young adults
Source: Nat Commun. 2026 Nov 14;17:11680. doi: 10.1038/s41467-026-78987-8 (PMC13769765; doi:10.1038/s41467-026-78987-8)
Supplement: Supplementary file 1 [file 41467_2026_78987_MOESM2_ESM.pdf]

Description of Additional Supplementary Files

Supplementary Data 1

Description: Seropositive and seronegative percentage per analyte for multi-analyte serology based on population-density cutoffs per sampling phase.

Supplementary Data 2

Description: Average percentage seropositivity per COVID antigen category and IFN AAbs per sampling phase.

Supplementary Data 3

Description: Prevalences of clinical traits in relation to presence or absence of autoantibodies to interferons across sampling phases.

Supplementary Data 4

Description: Multinomal logistic regression with IFNAR1 andor IFNGR2 AAb-status as predictor and long covid (symptoms 2 months or 3 months) as outcome.

Supplementary Data 5

Description: Spearman correlation between PC components and traits.

Supplementary Data 6

Description: Spearman correlation between PC components and proteins.

Supplementary Data 7

Description: Associations of protein levels with traits using linear models.

Supplementary Data 8

Description: Associations of protein levels with preanalytical factors (storage time) using mixed effect models adjusted for sex and including a random intercept for each subject.

Supplementary Data 9

Description: Spearman correlation between proteins and complete blood counts (CBC).

Supplementary Data 10

Description: Unadjusted linear regression: proteins ~ blood cells.

Supplementary Data 11

Description: Adjusted linear regression: proteins ~ blood cells + sex.

Supplementary Data 12

Description: Adjusted linear regression: proteins ~ blood cells + sex + smoking + BMI.

Supplementary Data 13

Description: Inter-correlation between proteins measured in plasma (24 year visit) and DBS (this study).

Supplementary Data 14

Description: GWAS hits.

Supplementary Data 15

Description: Intra-class correlations for proteins between sampling phases.

Supplementary Data 16

Description: Longitudinal Spearman correlation for proteins between sampling phases.

Supplementary Data 17

Description: Difference in change of protein levels for seronegative subjects who remain negative vs seronegative subjects who get infected. Where seronegative group who remained seronegative have been randomly bootstrapped over 100 iterations.

Supplementary Data 18

Description: Difference of protein levels for seronegative subjects who remain negative vs seronegative subjects who get infected within each sampling phase. Where seronegative group who remained seronegative have been randomly bootstrapped over 100 iterations.

Supplementary Data 19

Description: Difference of protein levels for previously infected subjects (infected at baseline Phase 1) and subjects who became infected (recently infected in Phase 2).

Supplementary Data 20

Description: Difference of protein levels for seronegative subjects who become vaccinated and infected vs subjects who were previously infected and got vaccinated within each sampling phase.

Supplementary Data 21

Description: Fisher's exact test for compare the number of samples above z-score = 2 or below z-scores = -2 before and after infection or vaccination.

Supplementary Data 22

Description: Proteins included in the suspension bead array (SBA) assay.

Supplementary Data 23

Description: Overview of statistical methods.
