# Supplementary material for: Molecular profiling of repeated self-sampled blood reveals dynamic immune phenotypes in young adults
Source: Nat Commun. 2026 Nov 14;17:11680. doi: 10.1038/s41467-026-78987-8 (PMC13769765; doi:10.1038/s41467-026-78987-8)
Supplement: Supplementary file 2 [file 41467_2026_78987_MOESM4_ESM.pdf]

nature portfolio

Corresponding author(s): JOCHEN SCHWENK, ERIK MELEN

Last updated by author(s): 15 JULY 2026

Reporting Summary

Nature Portfolio wishes to improve the reproducibility of the work that we publish. This form provides structure for consistency and transparency in reporting. For further information on Nature Portfolio policies, see our Editorial Policies and the Editorial Policy Checklist.

Statistics

For all statistical analyses, confirm that the following items are present in the figure legend, table legend, main text, or Methods section.

n/a	Confirmed	
☐	☒	The exact sample size (n) for each experimental group/condition, given as a discrete number and unit of measurement
☐	☒	A statement on whether measurements were taken from distinct samples or whether the same sample was measured repeatedly
☐	☒	The statistical test(s) used AND whether they are one- or two-sided<br>*Only common tests should be described solely by name; describe more complex techniques in the Methods section.*
☐	☒	A description of all covariates tested
☐	☒	A description of any assumptions or corrections, such as tests of normality and adjustment for multiple comparisons
☐	☒	A full description of the statistical parameters including central tendency (e.g. means) or other basic estimates (e.g. regression coefficient) AND variation (e.g. standard deviation) or associated estimates of uncertainty (e.g. confidence intervals)
☐	☒	For null hypothesis testing, the test statistic (e.g. F, t, r) with confidence intervals, effect sizes, degrees of freedom and P value noted<br>*Give P values as exact values whenever suitable.*
☒	☐	For Bayesian analysis, information on the choice of priors and Markov chain Monte Carlo settings
☐	☒	For hierarchical and complex designs, identification of the appropriate level for tests and full reporting of outcomes
☐	☒	Estimates of effect sizes (e.g. Cohen's d, Pearson's r), indicating how they were calculated

Our web collection on statistics for biologists contains articles on many of the points above.

Software and code

Policy information about availability of computer code

Data collection	Web-based questionnaires; Capitainer AB quantitative DBS devices; Luminex FlexMap 3D (serology/AAb); Olink Explore 384 Inflammation + Olink Target 96 (proteomics); Olink NPX Explore Software v3.0.5; Illumina NGS readout; Tanita MC-780 (body composition)
Data analysis	R version 4.4.1 (R Core Team, 2023); STATA SE 19.5 ; Julia version 1.7.2 (for serology normalization mixed model) ; Plink 2.0 (GWAS) ; METAL (meta-analysis) ; GCTA-COJO (conditional/joint pQTL analysis) ; R packages: Tidyverse 2.0.0, ggplot2 3.5.1, patchwork 1.3.0, RColorBrewer 1.1-3, ggh4x 0.3.1, ggbeeswarm 0.7.2, ggrepel 0.9.5, ggsignif 0.6.4, GGally 2.4.0, ComplexHeatmap 2.18.0, viridis 0.6.5, Ghibli 0.3.4, ggalluvial 0.12.5, psych 2.4.6.26, lmerTest 3.1-3, rstatix, gplots (heatmap.2), pheatmap, gridExtra, rcartocolor, UpSetR, ProtPQN 1.0.1

For manuscripts utilizing custom algorithms or software that are central to the research but not yet described in published literature, software must be made available to editors and reviewers. We strongly encourage code deposition in a community repository (e.g. GitHub). See the Nature Portfolio guidelines for submitting code & software for further information.

Data

Policy information about availability of data

All manuscripts must include a data availability statement. This statement should provide the following information, where applicable:

- Accession codes, unique identifiers, or web links for publicly available datasets
- A description of any restrictions on data availability
- For clinical datasets or third party data, please ensure that the statement adheres to our policy

All data associated with this study will be made available upon publication, its supplementary information or in the Source data, or may be obtained upon request

from the corresponding author (Erik Melen). Due to consent and legal constraints, the individual-level data cannot be deposited openly. Access to individual-level data can be granted for both non-commercial validation or collaborative purposes and upon request to the corresponding author. A request should contain the following: the name of PI and host organization, contact details, the scientific purpose of the data access request, the commitment to inform when the data has been used in a publication, the commitment not to host or share the data outside the requesting organization, and a statement of non-commercial use of data. Data access requests will be responded to within four weeks unless ethical constraints require new ethical permits, and the data will be available for a mutually decided timeframe. Source data are provided with this paper.

Research involving human participants, their data, or biological material

Policy information about studies with human participants or human data. See also policy information about sex, gender (identity/presentation), and sexual orientation and race, ethnicity and racism.

Reporting on sex and gender	In the study cohort, sex was determined by birth data from the Swedish National Board of Health and Welfare ("Socialstyrelsen" in Swedish). Distribution of clinical variables are displayed stratified for sex in Table 1.
Reporting on race, ethnicity, or other socially relevant groupings	The study included Swedish participants, >95% of white descent. No variables related to social grouping was used in the analysis.
Population characteristics	Population characteristics are described in Table 1. 808 young adults from the Swedish BAMSE birth cohort (born 1994–1996); 64% females; aged 24–27 at sampling; cohort originally 4,089 children.
Recruitment	Participants were existing members of the BAMSE birth cohort invited to a COVID-19 follow-up (divided into four phases; three phases used here: Phase 1 Aug–Nov 2020; Phase 2 Oct 2020–Jun 2021; Phase 3 Oct 2021–May 2022). Self-selection/ attrition bias possible as an existing cohort of health-engaged participants; potential bias toward compliant, health-conscious individuals given the self-sampling design
Ethics oversight	Regional Ethical Review Board Stockholm (DNR 2016/1380-31/2); Swedish Ethical Review Authority (DNR 2020-02922; 2024-06052-02). Informed written consent obtained from all subjects.

Note that full information on the approval of the study protocol must also be provided in the manuscript.

Field-specific reporting

Please select the one below that is the best fit for your research. If you are not sure, read the appropriate sections before making your selection.

- [x] Life sciences
- [ ] Behavioural & social sciences
- [ ] Ecological, evolutionary & environmental sciences

For a reference copy of the document with all sections, see nature.com/documents/nr-reporting-summary-flat.pdf

Life sciences study design

All studies must disclose on these points even when the disclosure is negative.

Sample size	Determined by cohort availability (all consenting BAMSE COVID-19 follow-up participants with DBS).
Data exclusions	- 28 of 678 genotype-matched samples excluded during pQTL QC (final GWAS set n=650)<br>- Plasma Olink Target 96 samples deviating >0.3 NPX from the internal control median were excluded<br>- Basophil counts excluded from CBC downstream analyses due to pronounced skewness toward zero<br>- SNPs filtered at MAF > 0.01 and imputation quality > 0.3 per wave<br>- Samples from Serocluster 2/3 (previously infected) excluded from the vaccination-only control analysis to avoid confounding by infection
Replication	Cross-platform/cross-cohort concordance used as validation: cis-pQTL overlap with UKB-PPP/SCALLOP/OpenGWAS; DBS vs plasma protein correlation (51 shared proteins); DBS vs ELISA serology (Cohen's kappa). Two independent genotyping waves meta-analyzed.
Randomization	Observational cohort, hence not applicable for group allocation. Bootstrap random sampling (100 iterations) used to balance unequal group sizes; random disc transfer in sample prep; random positioning of samples in analyses.
Blinding	Data generation was conducted in a blinded manner.

Reporting for specific materials, systems and methods

We require information from authors about some types of materials, experimental systems and methods used in many studies. Here, indicate whether each material, system or method listed is relevant to your study. If you are not sure if a list item applies to your research, read the appropriate section before selecting a response.

Materials & experimental systems

n/a	Involved in the study	
[ ]	[x]	Antibodies
[x]	[ ]	Eukaryotic cell lines
[x]	[ ]	Palaeontology and archaeology
[x]	[ ]	Animals and other organisms
[ ]	[x]	Clinical data
[x]	[ ]	Dual use research of concern
[x]	[ ]	Plants

Methods

n/a	Involved in the study	
[x]	[ ]	ChIP-seq
[x]	[ ]	Flow cytometry
[x]	[ ]	MRI-based neuroimaging

Antibodies

Antibodies used	Proteomics assays provided by Olink, serology and autoantibody analysis based on previous Luminex assays
Validation	Validation conducted by Olink or as presented in previous study: https://doi.org/10.1016/j.isci.2025.114611

Clinical data

Policy information about clinical studies

All manuscripts should comply with the ICMJE guidelines for publication of clinical research and a completed CONSORT checklist must be included with all submissions.

Clinical trial registration	N/A
Study protocol	N/A
Data collection	N/A
Outcomes	N/A

Plants

Seed stocks	N/A
Novel plant genotypes	N/A
Authentication	N/A
