# Supplementary material for: Molecular profiling of repeated self-sampled blood reveals dynamic immune phenotypes in young adults
Source: Nat Commun. 2026 Nov 14;17:11680. doi: 10.1038/s41467-026-78987-8 (PMC13769765; doi:10.1038/s41467-026-78987-8)
Supplement: Supplementary file 3 [file 41467_2026_78987_MOESM5_ESM.pdf]

nature portfolio

Peer Review File

Molecular profiling of repeated self-sampled blood reveals dynamic immune phenotypes in young adults

Corresponding Author: Professor Jochen Schwenk

This file contains all reviewer reports in order by version, followed by all author rebuttals in order by version.

Version 0:

Reviewer comments:

Reviewer #1

(Remarks to the Author)
Summary
This manuscript presents a comprehensive longitudinal dried blood spot (DBS) self-sampling study in 808 young adults from the BAMSE birth cohort, conducted between 2020 and 2022. The authors integrate multianalyte serology, anti-interferon autoantibody (AAb) profiling, affinity proteomics (365 inflammation-related proteins), genome-wide pQTL analysis, and complete blood count (CBC) data to characterize dynamic molecular immune phenotypes in the context of SARS-CoV-2 infection and vaccination. Data-driven seroclustering is used to define four serological clusters reflecting combinations of infection and vaccination status; anti-IFN AAbs are linked to prolonged COVID-19-related symptoms; and numerous protein-trait, CBC-protein, and genetic associations are described. Finally, the authors identify transient, exposure-dependent changes in proteins such as LAP3 after infection and TIMP3 after vaccination, arguing that self-sampled DBS enable time-resolved molecular phenotyping relevant to precision medicine.
Overall, this is an innovative and ambitious study that uses repeated home sampling via DBS to analyze dynamic immune phenotypes in a relatively large, population-based cohort of young adults. The integration of serology, autoantibody profiling, targeted proteomics, genetic variation, and blood cell counts is a clear principal strength, and the focus on SARS-CoV-2 infection and vaccination is timely. However, given the complexity and breadth of the analyses, several aspects of the study design, outcome definitions, and statistical methodology require clarification and strengthening before this manuscript can be considered for publication in Nature Communications.

MAJOR CONCERNS
1. Clarity of primary objectives and hierarchy of analyses
The manuscript presents a very large number of analyses - seroclustering, DBS proteomics with trait associations (BMI, sex, MASLD, smoking), pQTL mapping, autoantibody profiling, CBC-protein correlations, and pre/post-exposure protein dynamics - and it therefore becomes difficult for the reader to understand what the main research questions are and which results should be considered central as opposed to exploratory. This fragmentation of focus also risks diluting the impact of the most novel findings.
The recommendation is to clearly state two or three main objectives upfront and to specify which analyses are primary versus exploratory. Aligning the structure of the Results and Discussion sections with this hierarchy would substantially improve readability and help calibrate the reader's expectations regarding the strength of the conclusions drawn.
2. Causal inference is overstated throughout
The most pervasive conceptual weakness of the manuscript is the tendency to frame observational associations as mechanistically informative. The study design, repeated cross-sectional sampling with exposure-unrelated (random) sampling events, fundamentally limits causal attribution. This is acknowledged briefly in the Discussion, but the framing in the Abstract and Results sections repeatedly implies directionality and biological causality that the data cannot support.
For example, the statement that "paired pre- and postexposure highlighted transient alterations in infection (e.g., LAP3) and vaccination (e.g., TIMP3)" is presented as if these proteins are mechanistically driven by those exposures, when in fact the temporal resolution of sampling (mean 29.5 days post-PCR for infection analyses) severely limits such conclusions. The authors should consistently and explicitly qualify all exposure-protein associations as correlational throughout the manuscript, not only in the Discussion.
3. Temporal resolution is inadequate for the claims made about dynamic phenotypes
The central claim of the paper (that self-sampled DBS can capture dynamic immune trajectories) is undermined by the sampling design. The three sampling phases span approximately 18 months, with the majority of subjects sampled at

intervals of many months. Only 55 subjects had matched pre- and post-infection samples for the infection analysis (n=110 samples total), and only 12 subjects had samples within 0–30 days post-PCR confirmation. This is explicitly acknowledged (lines 272–273), yet the authors continue to make broad claims about "transient alterations" and "short-term variability."
The reviewers strongly recommend that the authors either:
• Substantially temper the language throughout the manuscript to reflect the actual temporal granularity achievable with this design, or
• Provide a dedicated supplementary power analysis quantifying what temporal changes can reliably be detected given these sample sizes and sampling intervals.
The statement that "other immune markers with previous links to SARS-CoV-2 infections were missed due to a steeper postinfection decline" (lines 342–344) is speculative and, while plausible, is presented without supporting evidence.
4. Handling of multiple testing and statistical significance across analyses
The statistical analysis section is extensive and describes the use of FDR, Bonferroni thresholds, linear and mixed models, ICC, and bootstrapping. However, given the breadth of the analyses, it remains difficult to understand which specific tests were applied to which hypotheses, and whether all relevant corrections have been applied.
To improve transparency, the authors should include a concise summary paragraph (either in the main text or as a supplementary statistical overview) that lists each type of main analysis, specifies the number of tests performed and the correction method used, and states the significance threshold adopted. Specifically:
• The association between persistent anti-IFNGR2 and anti-IFNAR1 autoantibodies and prolonged COVID-19 symptoms is one of the manuscript's most clinically significant claims. However, the absolute numbers in the "always positive" groups are very small (e.g., IFNGR2: n=52; IFNAR1: n=46), and the analysis relies on Fisher's exact test without adjustment for multiple comparisons across the 21 interferons tested and the multiple symptom duration thresholds (≥2 and ≥3 months). A Bonferroni or FDR correction applied specifically to the AAb-symptom association tests is required.
• Confounders are not accounted for in the AAb-symptom analysis. Persistent anti-IFN AAb positivity could be associated with baseline immune dysregulation, pre-existing conditions, or other factors that independently predict prolonged symptoms. Even a basic multivariable logistic regression including sex, BMI, and serocluster membership is necessary before this association can be claimed.
It is also necessary to clearly specify which results are considered robust primary findings and which are exploratory.
5. Genetic confounding in the anti-IFNAR2 autoantibody analysis is not addressed
The authors report that anti-IFNAR2 autoantibodies increase significantly across phases (Tab. 3: 2.2% in Phase 1 to 4.5% in Phase 3) and include IFNAR2 within the panel of AAbs analysed in relation to COVID-19 outcomes. However, a critical source of genetic confounding is entirely unaddressed. A recent study by Malvestiti et al. (Int. J. Mol. Sci. 2026, 27(2), 992; https://doi.org/10.3390/ijms27020992) demonstrated that a low-frequency coding variant at the IFNAR2 locus (p.F8S) independently associates with severe COVID-19 (OR ~1.73, validated across over 2 million subjects from the COVID-19 Host Genetics Initiative) and is accompanied by reduced IFNAR2 mRNA expression and measurable alterations in adaptive immune cell activation.
This finding has direct implications for the present study. Subjects carrying the p.F8S variant may be simultaneously more susceptible to generating anti-IFNAR2 autoantibodies (via altered receptor expression and presentation) and more prone to severe or prolonged COVID-19 outcomes (for genetic reasons that are entirely independent of autoimmunity). The current analysis cannot distinguish between these two mechanisms.
Critically, the authors performed a pQTL analysis on the same BAMSE cohort using Phase 1 DBS proteomics and imputed genotype data (n=650). This resource should be directly leveraged to determine whether the IFNAR2 locus contributes a significant pQTL signal for the anti-IFNAR2 AAb levels, and whether adjustment for p.F8S carrier status modifies the observed AAb-symptom association. Failure to perform this cross-modal integration, when the genetic data are already available, represents a missed analytical opportunity and leaves the autoimmune interpretation of the IFNAR2 findings on uncertain ground.
6. The pQTL analysis lacks methodological justification for several key choices
The genome-wide pQTL analysis is presented as a major finding (664 pQTLs across 65 proteins), but several methodological choices are inadequately justified:
• Sample size and power: Only 650 subjects were included, split across two genotyping waves (Wave 1: n=95; Wave 2: n=555). The statistical power to detect trans-pQTLs, especially the 135 described as "novel", with n=95 in Wave 1 is extremely limited. A formal power calculation or sensitivity analysis restricted to Wave 2 alone is required.
• Novelty claims: The authors claim 135 novel trans-pQTLs, validated by eQTLGen in 54% of cases. A 46% non-replication rate is substantial. The authors do not adequately discuss whether these represent true DBS-specific positives (e.g., driven by cell-specific protein leakage during drying) or false positives amplified by the small Wave 1 sample.
• Phase 1 data only: The use of Phase 1 data alone for the pQTL analysis is reasonable in principle, but means the full cohort proteome data were not used. The authors should explicitly address whether this introduces selection bias, particularly given that Phase 1 seropositivity was already 13.9% (N+) and 15.1% (S+).
• Cis/trans definition: The ±1 Mb window for cis-pQTL classification is standard but should be explicitly justified in relation to secreted protein targets, which may not follow canonical cis-regulatory logic.
7. The DBS matrix effect on proteomics is insufficiently characterized
A fundamental analytical concern that pervades all proteomic findings is the impact of cellular contents on DBS eluates. The authors acknowledge (lines 305–312) that RBCs and WBCs leak proteins upon drying, and show that 27% of proteins are storage-sensitive and that intracellular proteins are enriched among storage-sensitive targets. However:
• The CBC-protein correlation analysis (Fig. 4c) reveals that leukocyte and neutrophil counts are positively correlated with a large number of proteins, including key analytes used in subsequent analyses (PLAUR, NCF2, OSM, LAT, CXCL3). The authors do not systematically adjust proteomic analyses for CBC parameters. For the infection-associated LAP3 findings in particular, it is essential to demonstrate that the LAP3 elevation is independent of leukocyte count, which is known to transiently increase during acute viral infection.
• The inverse correlation between hematocrit and several proteins (TFF2, FST, ANGPTL4) is noted but not adequately

explained. Given that females have lower hematocrit and the cohort is 64% female, this could introduce sex-confounding into protein level estimates.
• To what extent do the observed infection- and vaccination-associated proteomic changes reflect genuine systemic protein changes versus reactive leukocytosis or neutrophilia altering the DBS cellular composition? This question must be directly addressed.

MODERATE CONCERNS

8. Generalizability and precision medicine claims

The cohort consists of generally healthy young adults (aged approximately 24–27 years), with a limited age range, a relatively low prevalence of comorbidities and smoking (~12%), and high adherence to vaccination recommendations. The authors rightly acknowledge that this homogeneity reduces variability in certain traits and makes it difficult to construct clearly separated comparison groups. However, the vision of "precision medicine" framing in the Abstract and Introduction is not adequately reconciled with these limitations.

The findings, while internally valid within this cohort, should not be presented as immediately applicable to older, more diverse, or more clinically complex populations. The authors should rework the precision medicine framing to be explicitly scoped to the study context, or provide explicit discussion of what additional evidence would be needed before translation.

Furthermore, many of the protein associations with BMI and sex are statistically significant likely because of the large number of proteins tested and the cohort's homogeneity. Effect sizes (Cohen's d or equivalent) are rarely reported alongside p-values and FDR values. For a manuscript claiming clinical relevance, effect sizes are essential throughout.

9. Seroclustering methodology

The data-driven seroclustering approach (hierarchical clustering with Ward's D2 on six binder signals) is presented as a key analytical contribution, but the choice of four clusters appears data-driven without formal justification. The authors should report standard cluster validity indices (e.g., silhouette score, gap statistic) to justify k=4 as the optimal solution. Additionally, Serocluster 3 contains only 1 sample in Phase 1 and 3 samples in Phase 2 (Tab. 2), raising questions about the stability and interpretability of this cluster in early phases.

10. Sex as a biological variable is underanalyzed

With 64% female participants, sex is an important biological variable, and the volcano plot for sex-associated proteins (Fig. 4b) shows 198 significantly associated proteins. However, subsequent analyses (infection, vaccination, pQTL) only adjust for sex as a covariate without exploring sex-stratified effects. Given growing evidence for sex differences in COVID-19 immune responses, at minimum a sensitivity analysis or supplementary sex-stratified analysis of the key LAP3 and TIMP3 findings should be provided.

11. Bootstrapping procedures require clearer description

The bootstrapping strategy used to handle unequal group sizes is described in the Methods (lines 582–595), but the rationale for choosing n=100 iterations is not stated, and it is unclear whether results were stable across different numbers of iterations. The authors should report the variance in effect sizes across bootstrap iterations for the primary findings (LAP3, LGALS9).

12. ProtPQN normalization

The ProtPQN normalization method (reference 55 is a preprint) is used for all proteomic analyses, but its performance relative to standard Olink normalization (plate control normalization) is not benchmarked in the current dataset. A supplementary comparison of key findings under both normalization strategies would strengthen confidence in the results.

MINOR CONCERNS

Clarity and presentation

• The Abstract is dense and lists too many findings in a compacted manner, making it difficult to identify the primary contribution. The authors should clearly prioritize 2–3 main findings.
• Lines 46–53 contain near-verbatim repetition of the same sentence about DBS self-sampling ("To democratize health monitoring... an increasingly valuable alternative"). This should be edited.
• The term "dynamic immune phenotypes" in the title and throughout is used loosely. In immunology, "dynamic" implies time-resolved kinetics. The authors should define this term operationally at first use or replace it with more precise language.
• The manuscript uses the outdated term NAFLD (Non-Alcoholic Fatty Liver Disease) when referring to metabolic liver disease. In accordance with current international consensus, this should be replaced throughout with MASLD (Metabolic dysfunction-Associated Steatotic Liver Disease), the nomenclature adopted since 2023 by major hepatology societies (Delphi consensus, Hepatology 2023).
• Figure 5 is complex and the right-panel (vaccination) results for LAP3 show no obvious shift, which partly contradicts the claim that TIMP3 is vaccination-specific. A clearer visual differentiation of infection-specific versus vaccination-specific proteins would improve the figure's interpretability.

Statistical reporting

• Effect sizes (Cohen's d or equivalent) are rarely reported alongside p-values and FDR values. For a manuscript claiming clinical relevance and precision medicine applications, effect sizes are essential throughout.
• The distinction between statistical significance and biological or clinical meaningfulness is not consistently addressed. Many of the 133 BMI-associated proteins and 198 sex-associated proteins likely have small effect sizes that are statistically significant only due to the multiplicity of tests and the cohort's homogeneity.

Generalizability

• The cohort is 64% female, born in Stockholm between 1994–1996, with relatively low smoking rates (~12%) and mean BMI in the healthy range. The authors should explicitly state in both the Discussion and the Abstract that findings may not generalize to older, more diverse, or more clinically complex populations.
• The comparison to UK Biobank (UKB) data is used to validate protein-trait associations, but UKB is a plasma-based study in a much older and broader age range. While the authors acknowledge this, the comparison is used somewhat uncritically to claim validity where discrepancies are also observed (e.g., smoking-associated proteins absent in BAMSE).

Data availability

• The data availability statement notes that datasets are not publicly available due to legal and ethical regulations. The

authors should clarify what specifically can be shared (e.g., summary statistics from the pQTL analysis, aggregated protein-trait association results), as this would substantially increase the study's utility to the broader scientific community.

Reviewer #2

(Remarks to the Author)
I co-reviewed this manuscript with one of the reviewers who provided the listed reports. This is part of the Nature Communications initiative to facilitate training in peer review and to provide appropriate recognition for Early Career Researchers who co-review manuscripts.

Reviewer #3

(Remarks to the Author)
This is an interesting and well-executed longitudinal study using repeated self-sampled DBS from the BAMSE cohort to study SARS-CoV-2 serology, anti-interferon autoantibodies, inflammatory proteins, blood counts, and genetic variation. The repeated sampling design, registry-linked infection/vaccination data, and multimodal profiling are major strengths. Overall, the manuscript makes a useful contribution to remote immune/proteomic monitoring and demonstrates the potential of well-executed multiplexed serological and proteomic techniques to shed light on fundamental immunology in large cohort studies. The identification of transient infection- or vaccination-associated protein changes, such as LAP3, is particularly interesting.

The work is significant because it extends prior DBS serology/proteomics studies by integrating repeated sampling with registry and genetic data, and it should be useful for population-scale immune monitoring and molecular epidemiology. Overall, the methods are sound and the broad conclusions are supported, but some specific biological claims should be tempered or strengthened with additional analyses using the existing data. The points below are intended to help strengthen the manuscript and make it suitable for publication after revision.

Comments
1. Because repeated samples were collected from nearly all participants, both the SARS-CoV-2 antibody and AAb analyses could be strengthened by adding a within-subject change analysis. For example, the authors could assess fold-change (or another intra-subject normalization approach) from each individual’s first available sample. This would complement the population-threshold approach and make seroconversion trajectories easier to interpret.

2. The longitudinal AAb profiling is a strong part of the paper, but for the association with prolonged COVID-19 symptoms, please clarify the multiple-testing correction used.

3. Several results are currently presented mainly through p-values or FDR values. Reporting effect sizes with confidence intervals would make the biological relevance much clearer.

4. Potential storage effects on the detection of SARS-CoV-2 antibodies and AAb should be clearly acknowledged. If these effects cannot be directly addressed analytically, the limitation should be discussed.

5. The Results section should be more descriptive about the assays performed. In some places, the description of the techniques are a bit vague. For example, please state the proteomic method used in the Results rather than relying on citations only.

6. More information on LAP3 would be helpful, including additional discussion or speculation on possible mechanisms underlying its elevation after infection.

7. Line 58: The statement that anti-SARS-CoV-2 antibodies persist “up to a year,” is inaccurate as we now know they can persist for well over a year after infection or vaccination.

Version 1:

Reviewer comments:

Reviewer #1

(Remarks to the Author)
Comments to the Authors
Sex-stratified analyses (in response to R1#10).
The additional sex-stratified analyses presented in the rebuttal are informative and appropriately interpreted, particularly given the marked imbalance in the number of males and females within the infected cohort. LAP3 remains significantly associated with infection in females, whereas the corresponding analysis in males does not reach statistical significance after FDR correction. Importantly, however, only one male participant contributed a sample within 30 days of PCR-confirmed infection, which is also the time window in which the strongest LAP3 elevation was observed. Thus, the absence of statistical significance in males cannot readily be interpreted as evidence of a sex-specific effect. Given the relevance of this

analysis to the robustness and interpretation of the central LAP3 finding, please include the sex-stratified analyses in the Supplementary Materials, together with an explicit statement in the main text acknowledging the limited statistical power in the male subgroup.
rs2229207 variant and anti-IFNAR2 autoantibodies (in response to R1#5).
The additional analysis of rs2229207 carriage in relation to IFNAR2 autoantibody status and long-term COVID-19 symptoms is appropriate and reassuring. In particular, the authors report that inclusion of rs2229207 carriage in the relevant regression model does not materially alter the associations between IFNAR1/IFNGR2 autoantibodies and long-term symptoms. Since these analyses were performed specifically to address a potential source of genetic confounding, I recommend including the corresponding results as a supplementary table, with an appropriate reference in the main text. This would improve transparency and allow readers to assess the robustness of the findings without relying on the rebuttal letter.
Language regarding temporal resolution and "transient" alterations (in response to R1#3).
I appreciate the authors' clarification that the sampling design is intended to capture epidemiological trajectories rather than provide fine-grained immunological time resolution. Nevertheless, I recommend a final careful review of the manuscript to ensure that terms such as "transient alterations," "short-term variability," and similar temporal descriptions are consistently qualified throughout the text. This is particularly relevant because the most informative post-infection window contains a relatively limited number of observations and only a small number of participants were sampled very close to PCR-confirmed infection. The conclusions regarding temporal dynamics should therefore be framed in a way that is fully consistent with the temporal resolution actually supported by the data.
ProtPQN versus standard Olink normalization (in response to R1#12).
The authors have clarified that Olink intensity normalization and ProtPQN serve complementary purposes and that both normalization steps are incorporated into the analytical workflow. This addresses the methodological rationale raised in the first round. Nevertheless, if readily available and without requiring substantial additional analysis, it would be useful to report whether the principal findings, particularly LAP3 and TIMP3, remain qualitatively unchanged when considering standard Olink normalization alone. This could be provided briefly in the Supplementary Materials or, alternatively, mentioned in the text as a robustness assessment. I consider this a useful additional check rather than a major methodological concern.
Remaining points.
The other issues raised in the first round of review have, in my view, been adequately addressed through the additional analyses and/or proposed revisions. The corresponding changes and clarifications are satisfactory.

Reviewer #3

(Remarks to the Author)
I have reviewed the authors' responses and the revised manuscript, and I am satisfied that the concerns raised during review have been addressed. The revisions have strengthened the manuscript, and I consider it suitable for publication in its current form.

Open Access This Peer Review File is licensed under a Creative Commons Attribution 4.0 International License, which permits use, sharing, adaptation, distribution and reproduction in any medium or format, as long as you give appropriate credit to the original author(s) and the source, provide a link to the Creative Commons license, and indicate if changes were made.
In cases where reviewers are anonymous, credit should be given to 'Anonymous Referee' and the source.

The images or other third party material in this Peer Review File are included in the article's Creative Commons license, unless indicated otherwise in a credit line to the material. If material is not included in the article's Creative Commons license and your intended use is not permitted by statutory regulation or exceeds the permitted use, you will need to obtain permission directly from the copyright holder.
To view a copy of this license, visit https://creativecommons.org/licenses/by/4.0/

Response to Reviewers

Reviewer #1 (Remarks to the Author):

Summary
This manuscript presents a comprehensive longitudinal dried blood spot (DBS) self-sampling study in 808 young adults from the BAMSE birth cohort, conducted between 2020 and 2022. The authors integrate multianalyte serology, anti-interferon autoantibody (AAb) profiling, affinity proteomics (365 inflammation-related proteins), genome-wide pQTL analysis, and complete blood count (CBC) data to characterize dynamic molecular immune phenotypes in the context of SARS-CoV-2 infection and vaccination. Data-driven seroclustering is used to define four serological clusters reflecting combinations of infection and vaccination status; anti-IFN AAbs are linked to prolonged COVID-19-related symptoms; and numerous protein-trait, CBC-protein, and genetic associations are described. Finally, the authors identify transient, exposure-dependent changes in proteins such as LAP3 after infection and TIMP3 after vaccination, arguing that self-sampled DBS enable time-resolved molecular phenotyping relevant to precision medicine.

Overall, this is an innovative and ambitious study that uses repeated home sampling via DBS to analyze dynamic immune phenotypes in a relatively large, population-based cohort of young adults. The integration of serology, autoantibody profiling, targeted proteomics, genetic variation, and blood cell counts is a clear principal strength, and the focus on SARS-CoV-2 infection and vaccination is timely. However, given the complexity and breadth of the analyses, several aspects of the study design, outcome definitions, and statistical methodology require clarification and strengthening before this manuscript can be considered for publication in Nature Communications.

Answer: We thank the reviewer for the time and effort invested in this thorough review, which has greatly improved the manuscript. We have addressed all your comments point-by-point; please see the details below.

MAJOR CONCERNS

1. Clarity of primary objectives and hierarchy of analyses
The manuscript presents a very large number of analyses - seroclustering, DBS proteomics with trait associations (BMI, sex, MASLD, smoking), pQTL mapping, autoantibody profiling, CBC-protein correlations, and pre/post-exposure protein dynamics - and it therefore becomes difficult for the reader to understand what the main research questions are and which results should be considered central as opposed to exploratory. This fragmentation of focus also risks diluting the impact of the most novel findings.

The recommendation is to clearly state two or three main objectives upfront and to specify which analyses are primary versus exploratory. Aligning the structure of the Results and Discussion sections with this hierarchy would substantially improve readability and help calibrate the reader's expectations regarding the strength of the conclusions drawn.

Answer: We acknowledge that the manuscript covers multi-analyte investigations that can be challenging to condense to a few key aspects. As we present a wide variety of advanced molecular analyses using an emerging sample type (i.e. DBS), a major focus is on validating the utility of these investigations in a longitudinal manner based on a population cohort. There are several novel aspects (e.g. repeated population home sampling) that have previously remained underexplored, and similar clinically oriented outcomes from other studies are still lacking. We have tried to clarify the structure of the content regarding this comment.

See lines 74ff: *Our primary objectives were a) to demonstrate feasibility of DBS sample use for longitudinal multi-molecular profiling, b) to study trajectories of anti-SARS-CoV-2 antibodies, autoantibodies and associated protein biomarkers in a real-life, population-based setting, and c) to link immune and protein biomarkers with clinical outcomes, including long-term COVID-19 symptoms. By combining multi-analyte serology, autoimmune and proteomic data with genetic, clinical, registry and*

health history data, we revealed transient and persistent trajectories, highlighting molecular phenotypes before, during, and after SARS-CoV-2 exposure.

2. Causal inference is overstated throughout
The most pervasive conceptual weakness of the manuscript is the tendency to frame observational associations as mechanistically informative. The study design, repeated cross-sectional sampling with exposure-unrelated (random) sampling events, fundamentally limits causal attribution. This is acknowledged briefly in the Discussion, but the framing in the Abstract and Results sections repeatedly implies directionality and biological causality that the data cannot support. For example, the statement that "paired pre- and postexposure highlighted transient alterations in infection (e.g., LAP3) and vaccination (e.g., TIMP3)" is presented as if these proteins are mechanistically driven by those exposures, when in fact the temporal resolution of sampling (mean 29.5 days post-PCR for infection analyses) severely limits such conclusions. The authors should consistently and explicitly qualify all exposure-protein associations as correlational throughout the manuscript, not only in the Discussion.

Answer: We thank the reviewer for this suggestion. We have carefully gone through and adjusted the manuscript to avoid implying causal inference. As a note, temporal investigations within weeks rather than months or years are a challenge for population studies, so temporal resolution here has more of an epidemiological than an immunological connotation. Yet, we believe that real-life epidemiological studies with immunology and omics data (like ours) can inform about potential mechanisms, albeit not draw firm conclusions about causality.

3. Temporal resolution is inadequate for the claims made about dynamic phenotypes
The central claim of the paper (that self-sampled DBS can capture dynamic immune trajectories) is undermined by the sampling design. The three sampling phases span approximately 18 months, with the majority of subjects sampled at intervals of many months. Only 55 subjects had matched pre- and post-infection samples for the infection analysis (n=110 samples total), and only 12 subjects had samples within 0–30 days post-PCR confirmation. This is explicitly acknowledged (lines 272–273), yet the authors continue to make broad claims about "transient alterations" and "short-term variability."

The reviewers strongly recommend that the authors either:
• Substantially temper the language throughout the manuscript to reflect the actual temporal granularity achievable with this design, or

• Provide a dedicated supplementary power analysis quantifying what temporal changes can reliably be detected given these sample sizes and sampling intervals.

The statement that "other immune markers with previous links to SARS-CoV-2 infections were missed due to a steeper postinfection decline" (lines 342–344) is speculative and, while plausible, is presented without supporting evidence.

Answer: We appreciate the position of the reviewer, but as mentioned above, we argue that the temporal aspect should be seen from an epidemiological (weeks) angle rather than an immunological one (minutes/days).

We respectfully disagree with the reviewer's view that our study cannot catch temporal changes due to low population size and short time span. Our claims about "transient alterations" and "short-term variability" are valid within this population and timeframe. Also, the study set-up with our sampling intervals is in fact beneficial for capturing serological alterations, while we acknowledge that it may not be fully optimal to capture rapid protein alterations.

Population-based studies, including the current study, have their limitations, which we highlight in the manuscript (lines 455ff). We would like to further point out that our study is one of few longitudinal DBS studies conducted in a general, healthy and well-characterized population during an ongoing pandemic, and that the conclusions drawn are related to this population during this follow-up period, which captures

two phases before mass vaccination and one phase after. Large, real-life datasets with repeated measures remain scarce in the literature, especially those lacking links to registries.

The nature of samples and data collected were influenced by public guidelines on, for example isolation, vaccination and self-testing, of which the latter was discontinued half-way through the pandemic, as well as by what was feasibly possible to conduct during a pandemic. As we did not pre-enrich for disease phenotypes or provide lifestyle guidance on behavior, 55 random subjects is not a small number, given that there was no pre-selection of participants.

The statement in lines 342-344 (now 425ff) is based on data from a mouse study we conducted and references under ref 29. It clearly shows the rapid decline of postinfection inflammation within days. Again, we investigated a generally healthy population without severe cases that would present with spiked inflammation profiles. We have made this adjustment.

4. Handling of multiple testing and statistical significance across analyses

The statistical analysis section is extensive and describes the use of FDR, Bonferroni thresholds, linear and mixed models, ICC, and bootstrapping. However, given the breadth of the analyses, it remains difficult to understand which specific tests were applied to which hypotheses, and whether all relevant corrections have been applied.

To improve transparency, the authors should include a concise summary paragraph (either in the main text or as a supplementary statistical overview) that lists each type of main analysis, specifies the number of tests performed and the correction method used, and states the significance threshold adopted.

Specifically:

• The association between persistent anti-IFNGR2 and anti-IFNAR1 autoantibodies and prolonged COVID-19 symptoms is one of the manuscript's most clinically significant claims. However, the absolute numbers in the "always positive" groups are very small (e.g., IFNGR2: n=52; IFNAR1: n=46), and the analysis relies on Fisher's exact test without adjustment for multiple comparisons across the 21 interferons tested and the multiple symptom duration thresholds (≥2 and ≥3 months). A Bonferroni or FDR correction applied specifically to the AAb-symptom association tests is required.

• Confounders are not accounted for in the AAb-symptom analysis. Persistent anti-IFN AAb positivity could be associated with baseline immune dysregulation, pre-existing conditions, or other factors that independently predict prolonged symptoms. Even a basic multivariable logistic regression including sex, BMI, and serocluster membership is necessary before this association can be claimed.

It is also necessary to clearly specify which results are considered robust primary findings and which are exploratory.

Answer: We thank the reviewer for pointing this out. We have now added a new sheet to the extensive Supplementary Data file (Supplementary Data 23), where we summarize the objectives and related statistics (data type, data display, analysis and correction for multiple testing). We have also summarized the use of FDR correction on lines 625ff in Methods.

The following eight interferons were included in the downstream analysis due to their autoantibody-binding frequency: IFNGR2, IFNL3, IFNA2, IFNA8, IFNW1, IFNG, IFNAR1 and IFNL1 as stated on lines 158ff. We have now added a Supplementary Data 3 where we display sex, overweight/obesity, COVID-19 symptoms ≥2 months, COVID-19 symptoms ≥3 months, self-reported current or previous depression, vaccination in phase 3, confirmed infection defined as a SmiNet case in phase 3, and serocluster belonging in phase 3 in relation to the AAb groups (AAb+, AAb**–** and AAb+/**–**). In Supplementary Data 3 we display both the nominal and FDR-corrected p-values and highlight FDR-significant results in the Results-section.

Further, we have performed a multivariable logistic regression with IFNAR1 or IFNGR2 AAb-status as predictor and long covid symptoms (symptoms 2 months or 3 months) as outcome and added sex, BMI (normal weight vs overweight/obese), serocluster belonging phase 3, and self-reported current or previous depression in phase 1 in the regression model and used the AAb– group as reference. Of

note, we do not have access to data regarding baseline immune dysregulation or pre-existing conditions that have been implicated in auto-antibody production to interferons, besides self-reported current or previous depression from the phase 1 questionnaire. The associations between IFNAR1 or IFNGR2 AAb status with long-term symptoms remained convincingly stable after these additional adjustments, and these results are now described in the Results section lines 167ff and in Supplementary Data 4.

We would also like to acknowledge the common power challenge in population-based studies on autoimmune conditions, as these generally suffer from lower numbers of positive cases (which is also the case in our study despite rather large overall sample size). Hence, our observed frequencies are in line with previous reports.

Finally, we have now specified main objectives vs explorative analyses in the manuscript, which also relates to your Comment 1.

5. Genetic confounding in the anti-IFNAR2 autoantibody analysis is not addressed
The authors report that anti-IFNAR2 autoantibodies increase significantly across phases (Tab. 3: 2.2% in Phase 1 to 4.5% in Phase 3) and include IFNAR2 within the panel of AAbs analysed in relation to COVID-19 outcomes. However, a critical source of genetic confounding is entirely unaddressed. A recent study by Malvestiti et al. (Int. J. Mol. Sci. 2026, 27(2), 992; https://doi.org/10.3390/ijms27020992) demonstrated that a low-frequency coding variant at the IFNAR2 locus (p.F8S) independently associates with severe COVID-19 (OR ~1.73, validated across over 2 million subjects from the COVID-19 Host Genetics Initiative) and is accompanied by reduced IFNAR2 mRNA expression and measurable alterations in adaptive immune cell activation.

This finding has direct implications for the present study. Subjects carrying the p.F8S variant may be simultaneously more susceptible to generating anti-IFNAR2 autoantibodies (via altered receptor expression and presentation) and more prone to severe or prolonged COVID-19 outcomes (for genetic reasons that are entirely independent of autoimmunity). The current analysis cannot distinguish between these two mechanisms.

Critically, the authors performed a pQTL analysis on the same BAMSE cohort using Phase 1 DBS proteomics and imputed genotype data (n=650). This resource should be directly leveraged to determine whether the IFNAR2 locus contributes a significant pQTL signal for the anti-IFNAR2 AAb levels, and whether adjustment for p.F8S carrier status modifies the observed AAb-symptom association. Failure to perform this cross-modal integration, when the genetic data are already available, represents a missed analytical opportunity and leaves the autoimmune interpretation of the IFNAR2 findings on uncertain ground.

Answer: We thank the reviewer for sharing this interesting study. However, we did not include IFNAR2 in the downstream analysis related to clinical or COVID-19-related outcomes based on the low frequencies of subjects positive for autoantibodies towards this interferon (Table 3 in manuscript). The following interferons were included in the downstream analysis: IFNGR2, IFNL3, IFNA2, IFNA8, IFNW1, IFNG, IFNAR1 and IFNL1 (see also answer to comment 4), of which IFNGR2 and IFNAR1 AAbs show a solid association with long-term COVID-19 symptoms.

Since we did not investigate IFNAR2 AAbs further, we have not addressed this gene variant in our analysis of long-term symptoms. Still, and for transparency, we assessed the frequency of this gene variant (rs2229207) and found it to be 17.12%, of which 16.67% carried one copy and 0.45% carried two copies, in our total population of 808 subjects. Carriage was slightly more common in women as compared to men (19.2% vs 13.7%, $p=0.07$). We did not find any association between the gene variant and IFNAR2 AAb-status per sampling phase or across sampling phases, or in relation to long-term COVID-19 symptoms ($p > 0.05$ for all comparisons, see table below).

Table. rs2229207 carriage in relation to IFNAR2 AAb-status per sampling phase, AAb-status across sampling phases, and long-term COVID-19 symptoms. Stated are cases per total and (%).

	IFNAR2 AAb+ subjects per phase			IFNAR2 AAb-status across phases			Long-term C19 symptoms	
rs2229207	Phase 1	Phase 2	Phase 3	AAb+	AAb–	AAb+/–	≥2 months	≥3 months
carrier	4/111 (3.6)	2/112 (1.8)	5/113 (4.4)	1/111 (0.9)	103/111 (92.8)	7/111 (6.3)	14/129 (10.9)	13/129 (10.1)
non-carrier	12/543 (2.2)	18/537 (3.4)	26/544 (4.8)	7/532 (1.3)	494/532 (92.9)	31/532 (5.8)	83/617 (13.5)	73/617 (11.8)

We further tested to add carriage of this variant (0/1) into the multinominal logistic regression model now used to investigate the association between long-term covid symptoms and autoantibodies towards IFNAR1 and IFNGR2 AAbs (see also comment 4). This did not alter the associations between IFNAR1 or IFNGR2 AAbs and long-term symptoms (see table below). For space reasons, and given that the manuscript is already extensive, we suggest not including these new results in the manuscript.

Table. Multinominal logistic regression to investigate the association between IFNGR2 AAb-status and long-term COVID-19 symptoms, with rs2229207, sex, BMI, serocluster and current/previous depression added as co-variates.

	**IFNGR2**			**IFNAR1**		
	RRR	SE	Nominal p-value	RRR	SE	Nominal p-value
Symptoms ≥2m						
AAb+	6.9	3.7	<0.001[1]	4.5	2.6	0.008[1]
AAb+/– (converters)	2.4	1.5	0.148	1.3	0.9	0.682
Symptoms ≥3m						
AAb+	5.8	3.4	0.002[1]	4.3	2.6	0.017[1]
AAb+/– (converters)	2.2	1.5	0.235	1.0	0.8	0.986

[1]FDR-significant. AAb– subjects (seronegative in all phases) were used as the reference population.

6. The pQTL analysis lacks methodological justification for several key choices
The genome-wide pQTL analysis is presented as a major finding (664 pQTLs across 65 proteins), but several methodological choices are inadequately justified:

• Sample size and power: Only 650 subjects were included, split across two genotyping waves (Wave 1: n=95; Wave 2: n=555). The statistical power to detect trans-pQTLs, especially the 135 described as "novel", with n=95 in Wave 1 is extremely limited. A formal power calculation or sensitivity analysis restricted to Wave 2 alone is required.

Answer: We thank the reviewer for this comment. We acknowledge the relatively small size of Wave1 (n=95). To account for potential differences between the two genotyping waves, genome-wide pQTL association analyses for all QC-passed Olink proteins were performed using a meta-analysis framework, a standard approach for combining datasets while maximizing statistical power. Restricting the analysis to Wave 2 alone would reduce sample size and consequently decrease power, particularly for trans-pQTL discovery. Importantly, in Supplementary Data 14, column K (Direction), the two symbols represent one wave each and the directional concordance is 84.5%, 525 out of 626 variants that actually had complete directional data across the two waves (++ or --), 38 variants had data missing from one wave, while 101 had opposite direction. Of the novel trans-pQTL signals, 104 out of 135, or 77%, had directional concordance, indicating that these associations are not driven solely by the smaller Wave1 dataset. Together, the concordant effects across waves and the meta-analysis framework support the robustness of the reported novel trans-pQTL associations.

• Novelty claims: The authors claim 135 novel trans-pQTLs, validated by eQTLGen in 54% of cases. A 46% non-replication rate is substantial. The authors do not adequately discuss whether these represent true DBS-specific positives (e.g., driven by cell-specific protein leakage during drying) or false positives amplified by the small Wave 1 sample.

Answer: Thank you for your comment. The two waves of GWAS genotyping in the cohort merely reflect the fact that genotyping was done in two separate rounds, which is not uncommon in large studies. As per state-of-the-art protocols, we have analysed the two waves separately and then performed a meta-analysis to include all participants and increase power. This has been applied in numerous earlier publications with our cohort data (e.g. Demenais et al, Nat Genet 2018; Hernandez-Pacheco et al, ECliniMed 2024, Koprulu et al, Cell 2026) and we feel very confident that our approach is scientifically and statistically sound. Reassuringly, the effect directions are also consistent across the two waves as the meta-analysis is using the inverse-variance weighted methodology (please see Supplementary Data 14). Since the eQTLGen consortium measures mRNA expression in whole blood, whereas our study measures circulating protein abundance in DBS, we consider a 54% validation rate across completely different biological modalities (transcriptome to proteome) for trans-acting regulatory signals as high. Please see the following recent publication (Li et al, Nat Genet 2026. PMID: 41501544). Furthermore, trans-pQTLs are known to reflect indirect regulatory pathways, network effects, cellular composition differences and downstream biological processes. We acknowledge that the 46% non-replication may reflect several sources of heterogeneity beyond the DBS samples use, including smaller effect-sizes, differences in biological matrix, age structure, assay design and ancestry composition across cohorts.

Phase 1 data only: The use of Phase 1 data alone for the pQTL analysis is reasonable in principle, but means the full cohort proteome data were not used. The authors should explicitly address whether this introduces selection bias, particularly given that Phase 1 seropositivity was already 13.9% (N+) and 15.1% (S+).

Answer: Baseline pQTL discovery is conventionally performed on a single reference proteomic state to minimize temporal variability and better contextualise the germline genetic effects. The germline DNA will have small changes across phases, while proteomic measurements may vary more over time due to those temporal interactions. Accordingly, Phase 1 represents the baseline reference proteome in our young-adult population. The observed seropositivity rates mirror the real-world epidemiological snapshot of this age group at that specific time point and restricting the discovery set to a strictly unexposed subset would artificially under-represent the true population variance and diminish our statistical power. The manuscript text on page 25, line 2 has been updated accordingly to reflect this.

We acknowledge that longitudinal analyses of pQTL dynamics across the subsequent phases is an important future direction. However, such analyses address a distinct scientific question that warrants its own dedicated follow-up study, rather than diluting the primary focus of the present work, designed to characterise baseline proteomic architecture and its genetic determinants. This also makes sense considering the raised concerns regarding the complexity and breadth of analysis in this paper.

• Cis/trans definition: The ±1 Mb window for cis-pQTL classification is standard but should be explicitly justified in relation to secreted protein targets, which may not follow canonical cis-regulatory logic.

Answer: The cis/trans definitions in genetics refer to locus of encoding gene, not physical destination to protein product. The ±1 Mb window is a standard consortium rule, and we followed the standardized criteria used by the UKB-PPP, SCALLOP, and similar consortia protocols (see e.g. Fauman and Hyde, 2026 BMC Bioinformatics; PMID: 35527238). The manuscript text on page 25, line 32 has been updated accordingly to reflect this.

7. The DBS matrix effect on proteomics is insufficiently characterized
A fundamental analytical concern that pervades all proteomic findings is the impact of cellular contents on DBS eluates. The authors acknowledge (lines 305–312) that RBCs and WBCs leak proteins upon

drying, and show that 27% of proteins are storage-sensitive and that intracellular proteins are enriched among storage-sensitive targets.

However:
• The CBC-protein correlation analysis (Fig. 4c) reveals that leukocyte and neutrophil counts are positively correlated with a large number of proteins, including key analytes used in subsequent analyses (PLAUR, NCF2, OSM, LAT, CXCL3). The authors do not systematically adjust proteomic analyses for CBC parameters. For the infection-associated LAP3 findings in particular, it is essential to demonstrate that the LAP3 elevation is independent of leukocyte count, which is known to transiently increase during acute viral infection.

Answer: We thank the reviewers for highlighting the importance of ensuring that the observed LAP3 elevation is independent of increased cell counts during infection. To address this, we have looked at associations between LAP3 with total leukocyte counts in Phase 2 data (Supplementary Data 10-12), and LAP3 did not significantly associate with total leukocyte counts. The cell counts could not be factored into the categorical Fisher's exact test (Fig. 5d-g). Therefore, to directly test the independence of our findings, we have rerun our linear regression models (originally detailed in Methods, and Results) to additionally adjust for leukocyte, hematocrit, and platelet counts.

Adjusting for these CBC parameters did not alter the significance of LAP3 in any of the bootstrapping iterations (n = 100):

- Infected vs. random non-infected: The FDR remained highly significant at 2.56×10^{-5}, with a median β-coefficient of 0.491 (range: 0.39-0.60)
- Within-phase comparison of infected vs non-infected: The median FDR was 2.57×10^{-6}, with a median β--coefficient of 0.34 (range: 0.28-0.41).

These results indicate that the infection-associated elevation of LAP3 is independent of leukocyte and other blood cell counts. However, the CBC data were exclusively collected at the Phase 2 clinical examination, whereas proteomic data span all three phases, which is a limitation of the study design. For example, NFASC (Figure 5f) is associated with vaccination, which is mainly phase 3-related, when Omicron and vaccination were strong exposures. Consequently, while we cannot systematically adjust the entire three-phase proteomic dataset for CBC parameters, our Phase 2 sensitivity analysis indicates that cross-sectional cell-count fluctuations (Supplementary Data 10-12) did not drive our primary LAP3 findings. As for now, these results have not been incorporated into the manuscript due to the already extensive content.

Finally, regarding the other proteins noted by the reviewer (PLAUR, NCF2, OSM, LAT, CXCL3), these were highlighted during initial correlation screening but were not selected as downstream highlights or key conclusions in this study.

• The inverse correlation between hematocrit and several proteins (TFF2, FST, ANGPTL4) is noted but not adequately explained. Given that females have lower hematocrit and the cohort is 64% female, this could introduce sex-confounding into protein level estimates.

Answer: We have adjusted for sex in all analyses where plausible, except where we compare protein levels against clinical traits, which we later compare to the UK Biobank study. Sex is also adjusted for in the CBC-protein analysis, see Supplementary Data 11 and 12. Thus, residual sex-confounding effects on the protein associations are very unlikely given our adjustments.

• To what extent do the observed infection- and vaccination-associated proteomic changes reflect genuine systemic protein changes versus reactive leukocytosis or neutrophilia altering the DBS cellular composition? This question must be directly addressed.

Answer: The alterations in DBS protein levels can either depend on changes in cell counts (leakage) and/or in cell activation (secretion), or cell death due to for example tissue damage. We interpret your comment on "genuine systemic protein changes" to refer to protein changes unrelated to cell-related changes, but our understanding is that changes in protein levels in relation to infection are most likely

related to cellular changes (secretion or increased cell counts). However, by adding CBC-counts as a confounding factor in the analysis described above, we rule out cell count influence on our main findings, although we cannot rule out such influence in all our analyses.

By being among the first to include CBC-data into a large-scale proteomic study, our ambition was to corroborate possible biases and be transparent about factors that influence the circulating proteome. See also answers above regarding the CBC-counts and results in Figure 5d-g.

MODERATE CONCERNS

8. Generalizability and precision medicine claims
The cohort consists of generally healthy young adults (aged approximately 24–27 years), with a limited age range, a relatively low prevalence of comorbidities and smoking (~12%), and high adherence to vaccination recommendations. The authors rightly acknowledge that this homogeneity reduces variability in certain traits and makes it difficult to construct clearly separated comparison groups. However, the vision of "precision medicine" framing in the Abstract and Introduction is not adequately reconciled with these limitations.

The findings, while internally valid within this cohort, should not be presented as immediately applicable to older, more diverse, or more clinically complex populations. The authors should rework the precision medicine framing to be explicitly scoped to the study context, or provide explicit discussion of what additional evidence would be needed before translation.

Furthermore, many of the protein associations with BMI and sex are statistically significant likely because of the large number of proteins tested and the cohort's homogeneity. Effect sizes (Cohen's d or equivalent) are rarely reported alongside p-values and FDR values. For a manuscript claiming clinical relevance, effect sizes are essential throughout.

Answer: We thank the reviewer for this comment, and we do not claim that these findings are precision medicine ready for clinical use. Yet, precision medicine is now rapidly evolving from rare, severe diseases to also encompass common, complex conditions and the use of healthcare data (see e.g. Kämpe A et al, Nat Medicine 2025). Young adults remain an understudied population, as they are usually healthier than older adults, which has of course influenced our results. Thus, the revised version anchors on a molecular epidemiology framing.

Regarding the statistics and outcomes, we want to direct the reviewer to the Supplementary Data, which already display effect sizes such as β-coefficients/estimates and odds ratios. We have now added these to the Results section as well as making sure that we are referring to the corresponding supplementary data files in the text. We have also provided an overview of the different data analysis methods.

9. Seroclustering methodology
The data-driven seroclustering approach (hierarchical clustering with Ward's D2 on six binder signals) is presented as a key analytical contribution, but the choice of four clusters appears data-driven without formal justification. The authors should report standard cluster validity indices (e.g., silhouette score, gap statistic) to justify k=4 as the optimal solution. Additionally, Serocluster 3 contains only 1 sample in Phase 1 and 3 samples in Phase 2 (Tab. 2), raising questions about the stability and interpretability of this cluster in early phases.

Answer: This is a valid question we considered before the submission. Hierarchical clustering was performed using maximum distance and Ward's linkage and we used gap statistics to define the number of clusters. Although the gap statistics score continued to increase slightly with larger numbers of theoretical clusters, the gain in cluster separation diminished substantially beyond four clusters (Fig.1 below). Inspection of the dendrogram indicated that four clusters captured the major structure of the data, while solutions with larger numbers of clusters primarily subdivided existing groups and reduced interpretability. We also argue that the main exposures (infection and vaccination) create a combinatorial space of four clusters when treated as binary categories.

Therefore, a four-cluster solution was selected for downstream analyses. Cluster stability was assessed using bootstrap resampling, yielding mean Jaccard indices (JI) of 0.97, 0.80, 0.64, and 0.87 for the four clusters. Three clusters demonstrated good to excellent stability (JI > 0.75), while the remaining cluster showed moderate stability. Overall, the four-cluster solution provided a robust and biologically interpretable representation of the underlying data structure and was therefore selected for downstream analyses.

We have added a sentence about this to the Methods part of the manuscript lines 659ff.

Fig. 1. Gap statistics for the clustering. The dashed vertical line refers to the selected number of clusters.

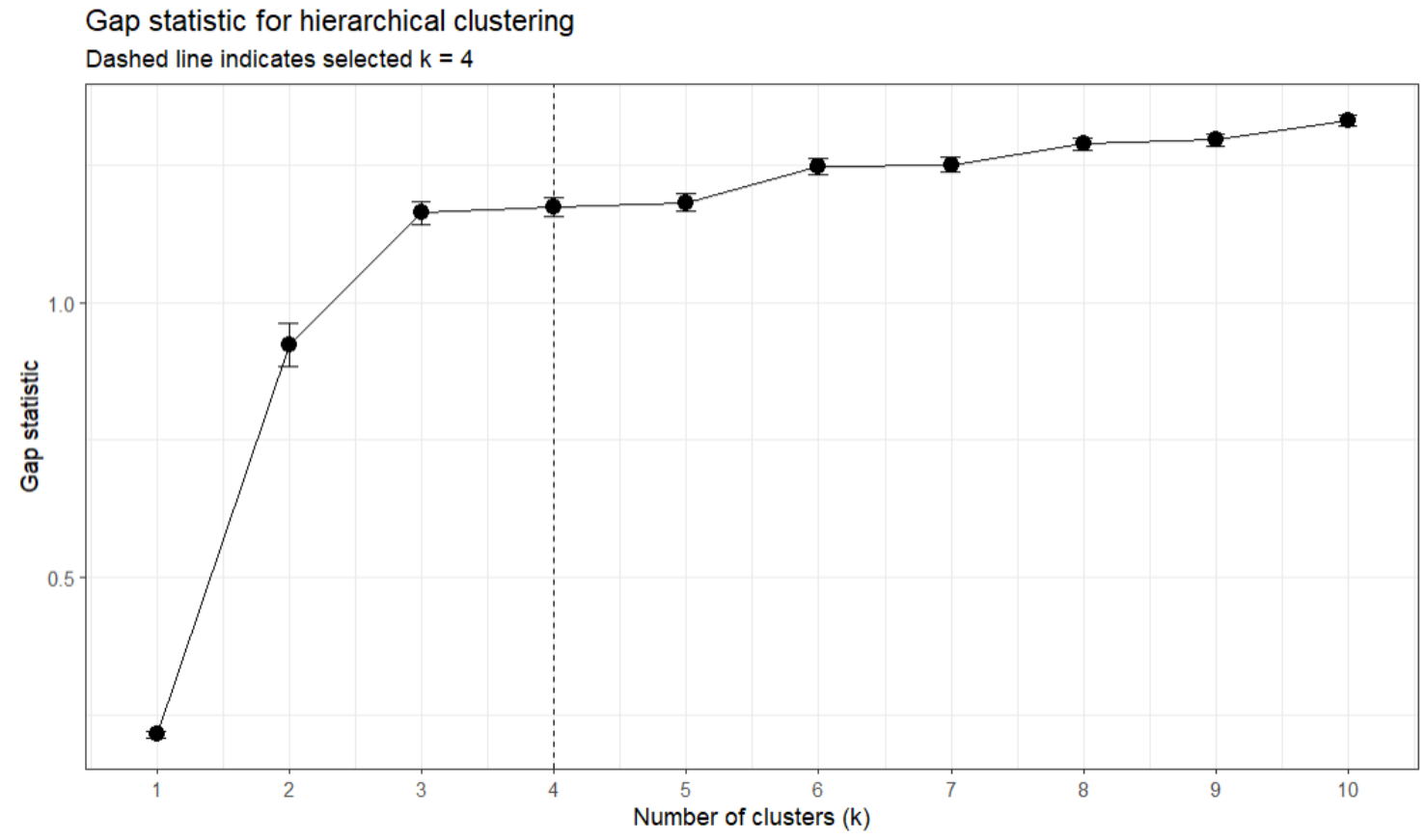

10. Sex as a biological variable is underanalyzed
With 64% female participants, sex is an important biological variable, and the volcano plot for sex-associated proteins (Fig. 4b) shows 198 significantly associated proteins. However, subsequent analyses (infection, vaccination, pQTL) only adjust for sex as a covariate without exploring sex-stratified effects. Given growing evidence for sex differences in COVID-19 immune responses, at minimum a sensitivity analysis or supplementary sex-stratified analysis of the key LAP3 and TIMP3 findings should be provided.

Answer: We thank the reviewer for this valuable suggestion. As mentioned in several comments above, sex is adjusted for in all analysis where plausible. We agree that exploring sex-specific effects is important given the documented sex-related differences in COVID-19 immune responses. LAP3 was indeed a protein with higher levels in females. Consequently, we have now performed sex-stratified analyses for the Fisher's exact test (comparing the number of samples outside the Z-scores ± 2 thresholds) in both the infected and vaccinated populations where LAP3 and TIMP3, among other proteins, were significant. For the infected group (n= 55 in total), LAP3 remains significantly elevated for the female group (n=39) with an adjusted odds ratio of 0.025 and FDR p-value=0.021. Notably, outlier levels were absent pre-infection (0/39) but appeared in 33.3% of females post-infection. This association was not statistically significant in males (n=16) after FDR correction but the trend towards higher LAP3 outlier frequencies post-infection remained with outliers occurring exclusively post-infection (3/16) giving an adjusted odds ratio = 0.12. However, we interpret this result with caution due to the limited sample size of the male subgroup. Crucially, only a single male participant provided a sample within 30 days of a PCR-confirmed infection, which was the time window where the highest elevation of LAP3 signals was observed. Therefore, we cannot definitively determine whether the LAP3 finding is sex-specific or a reflection of statistical power and timing constraints in the male population (Fig. 2 below). However, performing a matched pre- vs post-infection analysis mitigated some risks.

In sex-stratified analyses of the vaccinated group (n=475; 174 males and 301 females), TIMP3, LHPP, and SRPK2 remained significantly elevated in females post-vaccination (FDR adjusted p = 0.002, 0.020, and 0.038, respectively) but did not reach significance in males after FDR correction (FDR adjusted p = 0.17, 0.056, and 0.056, respectively). However, the adjusted odds ratios showed consistent directional trends across both sexes, remaining well below 1 for TIMP3, LHPP, and SRPK2 in both females (odds ratio = 0.03, 0.08, 0.09, respectively) and males (odds ratios = 0.05, 0.04, 0.10, respectively). Thus, there do not appear to be sex differences in these instances. NFASC was consistently and significantly decreased across both sexes, characterized by having more negative

outliers (Z < -2) post-vaccination (males: adjusted odds ratio = 0.02 and FDR p-value $3.8x10^{-4}$; females: adjusted odds ratio = 0.06 and FDR p-value $1.75x10^{-7}$).

Due to the already extensive content of the manuscript and the limited number of infected males in the LAP3 analysis, we have not included these sex-stratified analyses in the Results section.

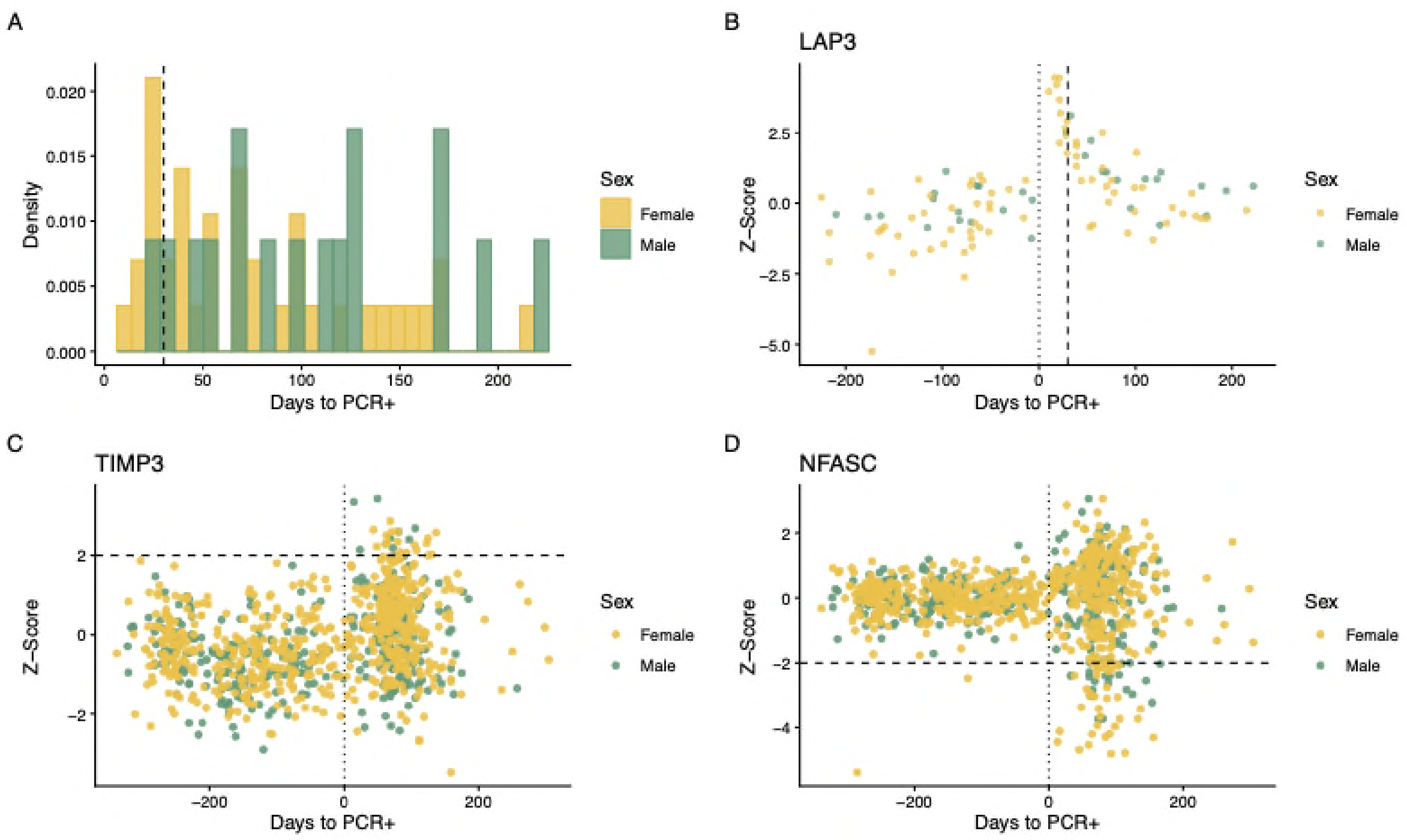

Fig. 2. Sex stratified analysis of protein levels following infection and vaccination. (A) Histogram showing postinfection samples from males (green) and females (yellow). (B-D) Protein z-scores plotted against days since PCR-confirmed infection for LAP3 and days since closest vaccination dose for TIMP3 and NFASC colored by sex.

11. Bootstrapping procedures require clearer description
The bootstrapping strategy used to handle unequal group sizes is described in the Methods (lines 582–595), but the rationale for choosing n=100 iterations is not stated, and it is unclear whether results were stable across different numbers of iterations. The authors should report the variance in effect sizes across bootstrap iterations for the primary findings (LAP3, LGALS9).

Answer: We thank the reviewer for the comment. We have clarified this matter in Supplementary Data 17 and 18 in the text. We reported mean and median estimates (betas), confidence intervals, the proportions of iterations where the estimate is negative/positive, and the proportions of iterations where the slope for the protein is significant (FDR < 0.05). Furthermore, we also calculated the variance of the estimates for the iterations, and the standard deviation, and added these to the text for LAP3 (variance = $7x10^{-4}$, SD = 0.03) and LGALS9 (variance = $2x10^{-4}$, SD = 0.01) for the within phase comparison between the groups. The small SDs indicated that the coefficient estimates are stable and robust to resampling. To further evaluate the robustness of the result, we used 500 iterations, and the difference in the median estimate between 100 and 500 iterations was 0.008 for LAP3 and 0.009 for LGALS9, verifying that the results remain essentially unchanged.

When comparing protein level changes between the I– and I+ subjects, LAP3 was significantly increased in the infected group for all 100 iterations with an iteration variance = $1.7x10^{-3}$, and iteration SD = 0.04. These numbers, together with the median estimate (beta), have been added to the text lines 313ff.

Table. Median and mean estimates for 100 iterations for comparing I+ and I- within sampling phases.

	sampling_phase	assay	median_median_diff	median_estimate	mean_estimate	lower_95	upper_95	prop_pos	prop_neg	prop_sig	n_non_na	fdr_median	fdr_mean
	<chr>	<chr>	<dbl>	<dbl>	<dbl>	<dbl>	<dbl>	<dbl>	<dbl>	<dbl>	<int>	<dbl>	<dbl>
1	Phase 2	LAP3	0.234	0.333	0.331	0.283	0.378	1	0	1	100	0.00000485	0.0000460
2	Phase 2	LGALS9	0.0823	0.0900	0.0895	0.0600	0.122	1	0	0.74	100	0.00611	0.0731

	assay	sampling_phase	n_boot	mean_coef	variance	sd
	<chr>	<chr>	<int>	<dbl>	<dbl>	<dbl>
1	LAP3	Phase 1	100	-0.150	0.000652	0.0255
2	LAP3	Phase 2	100	0.331	0.000664	0.0258
3	LGALS9	Phase 1	100	-0.0490	0.000260	0.0161
4	LGALS9	Phase 2	100	0.0895	0.000211	0.0145

Table. Median and mean estimates for 500 iterations for comparing I+ and I- within sampling phases.

	sampling_phase	assay	median_median_diff	median_estimate	mean_estimate	lower_95	upper_95	prop_pos	prop_neg	prop_sig	n_non_na	fdr_median	fdr_mean
	<chr>	<chr>	<dbl>	<dbl>	<dbl>	<dbl>	<dbl>	<dbl>	<dbl>	<dbl>	<int>	<dbl>	<dbl>
1	Phase 2	LAP3	0.231	0.325	0.326	0.278	0.378	1	0	1	500	0.00000904	0.0000740
2	Phase 2	LGALS9	0.0812	0.0884	0.0886	0.0614	0.117	1	0	0.734	500	0.00932	0.0621

	assay	sampling_phase	n_boot	mean_coef	variance	sd
	<chr>	<chr>	<int>	<dbl>	<dbl>	<dbl>
1	LAP3	Phase 1	500	-0.149	0.000708	0.0266
2	LAP3	Phase 2	500	0.326	0.000678	0.0260
3	LGALS9	Phase 1	500	-0.0480	0.000281	0.0168
4	LGALS9	Phase 2	500	0.0886	0.000200	0.0142

12. ProtPQN normalization

The ProtPQN normalization method (reference 55 is a preprint) is used for all proteomic analyses, but its performance relative to standard Olink normalization (plate control normalization) is not benchmarked in the current dataset. A supplementary comparison of key findings under both normalization strategies would strengthen confidence in the results.

Answer: We would like to respectfully correct the reviewer's perception, as ProtPQN has been introduced and discussed before (Fredolini et al., 2024 Comms Med; Yu et al., 2025 iScience; Dahl et al., 2026 iScience) and it is based on earlier work (Dodig-Crnkovic et al., 2020 EBioMed). ProtPQN is solely intended to control for the effects of layperson sampling. This is not addressed by the classical Olink approach.

As stated in the methods section (lines 797ff), the proteomics analyses use both the standard Olink normalization, here the intensity normalization as the plates were randomized, and ProtPQN. The Olink intensity normalization is meant to reduce variability between assay plates while ProtPQN reduces the sample-to-sample variability within plates akin to PQN but in a gentler manner by considering that different antibodies have different affinities. These normalization approaches fulfil different and complementary purposes and a benchmarking of one vs the other is not particularly meaningful for our key findings.

MINOR CONCERNS

Clarity and presentation

- The Abstract is dense and lists too many findings in a compacted manner, making it difficult to identify the primary contribution. The authors should clearly prioritize 2–3 main findings.

Answer: We have adjusted the abstract in line with this comment.

"Our primary objectives were a) to demonstrate feasibility of DBS sample use for longitudinal multi-molecular profiling, b) to study dynamic trajectories of anti-SARS-CoV-2 antibodies, autoantibodies and associated protein biomarkers in a real-life, population-based setting, and c) to link immune and protein biomarkers with clinical outcomes, including long-term COVID-19 symptoms."

- Lines 46–53 contain near-verbatim repetition of the same sentence about DBS self-sampling ("To democratize health monitoring... an increasingly valuable alternative"). This should be edited.

Answer: We have adjusted the sections in line with this comment as follows in lines 55ff: "*To enable large-scale monitoring in a more patient-centric manner, self-sampling of dried blood spots (DBS) has become increasingly attractive as a valuable alternative to venous blood draws. By simplifying access*

to samples, new types of molecular data can be generated without in-clinic visits, contributing to democratizing health care"

• The term "dynamic immune phenotypes" in the title and throughout is used loosely. In immunology, "dynamic" implies time-resolved kinetics. The authors should define this term operationally at first use or replace it with more precise language.

Answer: As mentioned earlier, in epidemiological studies, we believe that "dynamic" refers to change, movement, and adaptation within the immune system, reflecting constant adaptation to the environment and different exposures. We have defined our interpretation of "dynamic" on lines 75f: "*dynamic trajectories of anti-SARS-CoV-2 antibodies, autoantibodies and associated protein biomarkers in a real-life, population-based setting* "

• The manuscript uses the outdated term NAFLD (Non-Alcoholic Fatty Liver Disease) when referring to metabolic liver disease. In accordance with current international consensus, this should be replaced throughout with MASLD (Metabolic dysfunction-Associated Steatotic Liver Disease), the nomenclature adopted since 2023 by major hepatology societies (Delphi consensus, Hepatology 2023).

Answer: Thank you for pointing this out. We removed NAFLD since we did not investigate MASLD in the study.

• Figure 5 is complex and the right-panel (vaccination) results for LAP3 show no obvious shift, which partly contradicts the claim that TIMP3 is vaccination-specific. A clearer visual differentiation of infection-specific versus vaccination-specific proteins would improve the figure's interpretability.

Answer: We thank the reviewer for this comment. Our understanding is that the non-existing shift in LAP3 does not contradict a shift in TIMP3 due to vaccination since the two proteins may have different expression profiles following immune stimulation. We have shown that LAP3 is related to infection but not vaccination, and that TIMP3 is related to vaccination but not to infection.

We have now added p-values to the figure legend and added headings to the panels in Figure 5d-g, to better highlight which changes are infection-related versus vaccination-related, respectively. While these plots might be perceived as complex, they illustrate how changes over time occur, hence providing transparent access to these alterations. We prefer to keep these as they are.

Statistical reporting

• Effect sizes (Cohen's d or equivalent) are rarely reported alongside p-values and FDR values. For a manuscript claiming clinical relevance and precision medicine applications, effect sizes are essential throughout.

Answer: Effect sizes such as slopes' β values, odds ratios, and others have been added to the manuscript. All information was already present in the Supplementary Data. Please see also our answers to several previous related comments.

• The distinction between statistical significance and biological or clinical meaningfulness is not consistently addressed. Many of the 133 BMI-associated proteins and 198 sex-associated proteins likely have small effect sizes that are statistically significant only due to the multiplicity of tests and the cohort's homogeneity.

Answer: Effect sizes have been provided (see answers to previous comments), however, it is beyond the scope of this manuscript to determine which of the data that is clinically meaningful, and we have systematically reviewed the manuscript once more to tune down any immediate clinical implications.

Generalizability
• The cohort is 64% female, born in Stockholm between 1994–1996, with relatively low smoking rates (~12%) and mean BMI in the healthy range. The authors should explicitly state in both the Discussion and the Abstract that findings may not generalize to older, more diverse, or more clinically complex populations.

Answer: We fully agree and have now stated this in the abstract and discussion.

• The comparison to UK Biobank (UKB) data is used to validate protein-trait associations, but UKB is a plasma-based study in a much older and broader age range. While the authors acknowledge this, the comparison is used somewhat uncritically to claim validity where discrepancies are also observed (e.g., smoking-associated proteins absent in BAMSE).

Answer: We agree with the reviewer, but we wanted to use the most elaborate study to benchmark our data. The main purpose of conducting the comparison to the UKB was to show trait-associations in DBS and "gold standard" plasma. We are fully aware that UKB-data is based on plasma and that the population is very different from our population.

We agree with the reviewer that the discrepancy in particular for smoking prevents a useful comparison of this variable; however, it illustrates the differences between cohorts, which are important as a balance to those findings with congruent demographics and traits.

Of note, in Supplementary figure 9, we are comparing trait associations to another DBS study, but with a different population.

Data availability
• The data availability statement notes that datasets are not publicly available due to legal and ethical regulations. The authors should clarify what specifically can be shared (e.g., summary statistics from the pQTL analysis, aggregated protein-trait association results), as this would substantially increase the study's utility to the broader scientific community.

Answer: We agree to share our data according to scientific standards (and Swedish law) when possible. While primary data will remain under restricted access, the supplementary information already contains substantial amounts of summary statistics and details (e.g. GWAS summary statistics).

Upon acceptance of this manuscript, this summary data can also be deposited on Zenodo as requested by the journal editor. We will also deposit the code on GitHub (e.g. https://github.com/Schwenk-Lab), which will assist reproducing the figures. As a note, no new analysis codes were developed.

Reviewer #2 (Remarks to the Author):

I co-reviewed this manuscript with one of the reviewers who provided the listed reports. This is part of the Nature Communications initiative to facilitate training in peer review and to provide appropriate recognition for Early Career Researchers who co-review manuscripts.

Answer: We thank the co-reviewer for contributing to improving the manuscript.

Reviewer #3 (Remarks to the Author):

This is an interesting and well-executed longitudinal study using repeated self-sampled DBS from the BAMSE cohort to study SARS-CoV-2 serology, anti-interferon autoantibodies, inflammatory proteins, blood counts, and genetic variation. The repeated sampling design, registry-linked infection/vaccination data, and multimodal profiling are major strengths. Overall, the manuscript makes a useful contribution to remote immune/proteomic monitoring and demonstrates the potential of well-executed multiplexed serological and proteomic techniques to shed light on fundamental immunology in large cohort studies. The identification of transient infection- or vaccination-associated protein changes, such as LAP3, is particularly interesting.

The work is significant because it extends prior DBS serology/proteomics studies by integrating repeated sampling with registry and genetic data, and it should be useful for population-scale immune monitoring and molecular epidemiology. Overall, the methods are sound and the broad conclusions are supported, but some specific biological claims should be tempered or strengthened with additional analyses using the existing data. The points below are intended to help strengthen the manuscript and make it suitable for publication after revision.

Answer: We thank the reviewer for the thorough review and the very positive first assessment of our work. It is constructive comments like these that have helped us to improve the manuscript.

Comments

1. Because repeated samples were collected from nearly all participants, both the SARS-CoV-2 antibody and AAb analyses could be strengthened by adding a within-subject change analysis. For example, the authors could assess fold-change (or another intra-subject normalization approach) from each individual's first available sample. This would complement the population-threshold approach and make seroconversion trajectories easier to interpret.

Answer: We thank the reviewer for the comment and agree that centering the data on individual level baselines can be a meaningful addition to our analyses. We have indeed discussed this during the preparation of the work, and centering the data per individual is one option to normalize changes over time. However, the strong effect of infection and vaccination during phase 3 would strongly influence the outcome of this analysis.

For **SARS-CoV-2 serology**, infection and vaccination exposures had, as expected, a very strong impact on the antibody levels. Hence, baseline levels for these analytes were expected to be negative for some antigen variants (e.g. anti-omicron-RBD) while others were already elevated in some participants (e.g. anti-N or anti-wt-S), and the amplitude of change is highly dependent on the timing of sampling in relation to exposures.

As shown below, we computed the Z-scores per antigen to display the changes in a unified format across all antigens. Scores for anti-S and anti-RBD increase in relation to Omicron and vaccine roll-out. For anti-N, elevated baseline levels could relate to previous infections, and those occurring during Phase 2.

In addition, we used normalized data to determine the relative changes of Phases 2 and 3 versus the baseline sampling. As shown below, the major changes to z-scores were related to N-positivity at Phase 1. This can be explained by early infections, leading also to a relative decline in titers during the later sampling phases. As shown above, some individuals had increasing relative anti-N Abs levels in Phase 2 and 3. The plots above and below also show that some participants remained S-negative throughout all sampling phases.

Z-score figure: Light grey lines are population z-scores per individual, and the thick black line is the average trend via LOESS regression function across all individuals.

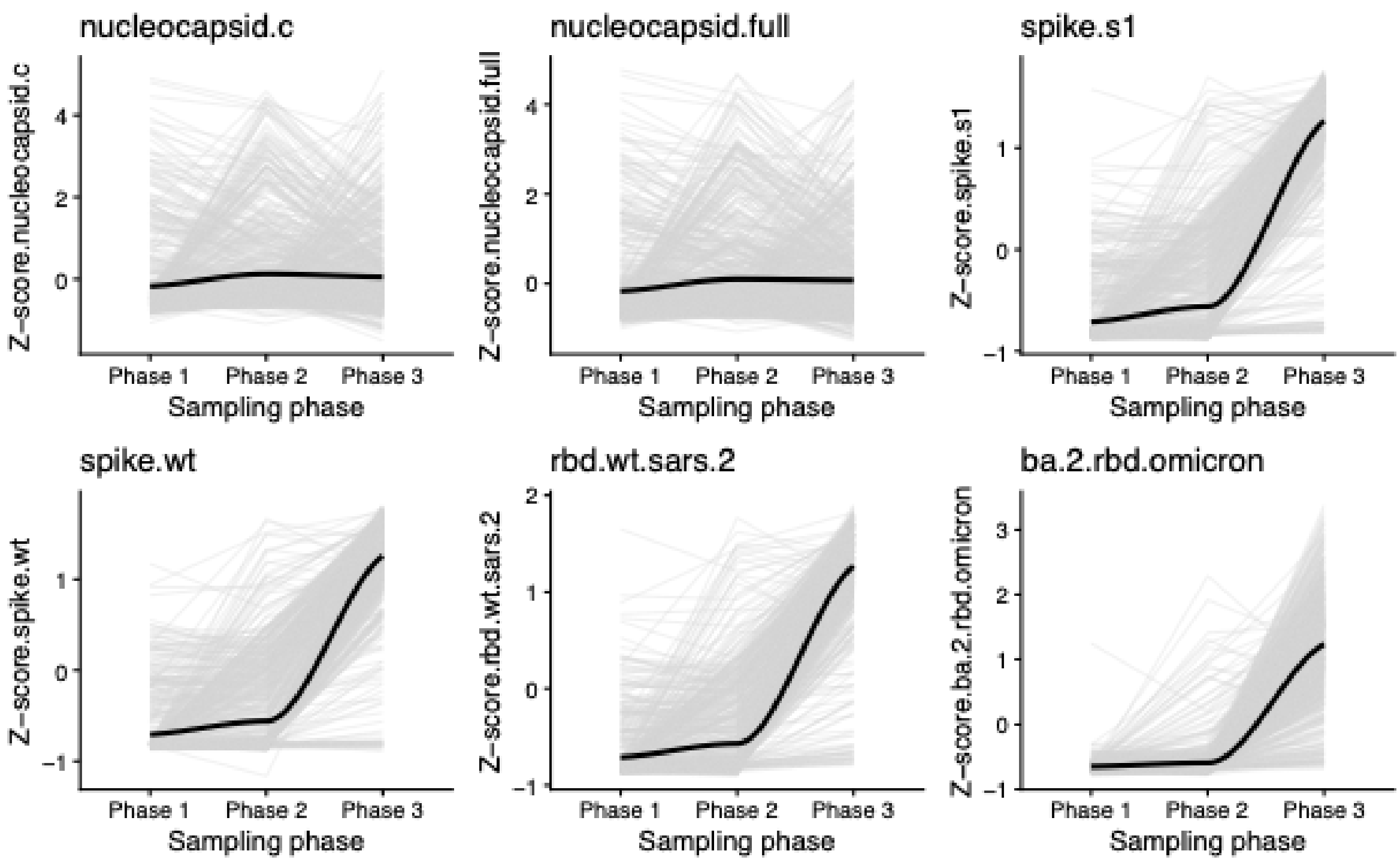

Relative change figure: Light grey lines are changes to baseline per individual, and the thick black line is the average trend via LOESS regression function across all individuals.

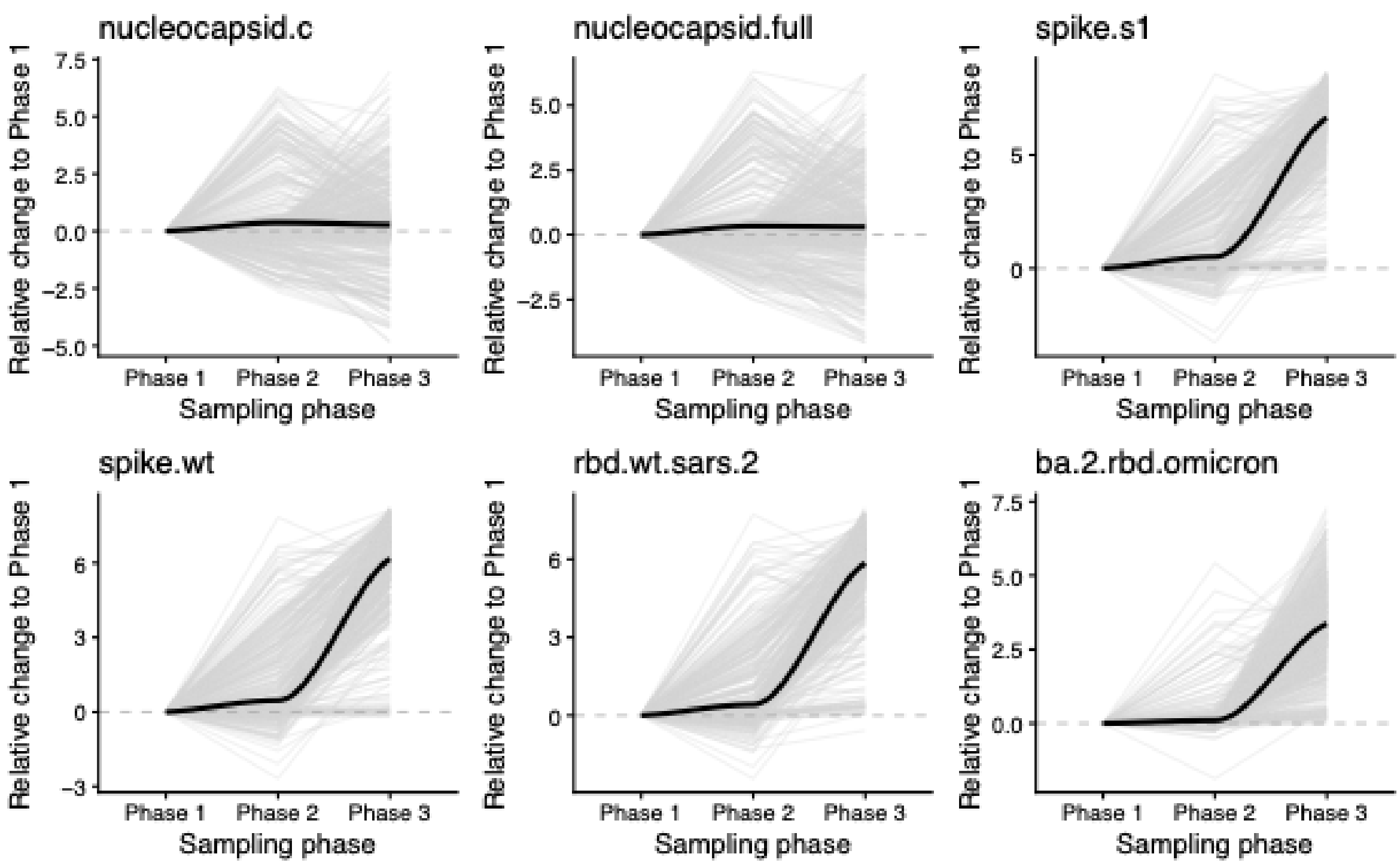

A primary challenge in serology is how to interpret the amplitude of the response, as several factors influence these measures (e.g. epitope, affinity, avidity, Ab concentration). Consequently, as it remains challenging to obtain control samples for DBS, we used the population to determine cut-offs, as these most closely resembled the molecular phenotypes.

For the **AAbs analysis**, we indeed focused on those showing consistent AAb levels over time to avoid enriching the outcomes for shorter-lived spikes in presumed AAb levels. Normalizing the data on each subject would highlight those with changes in AAb levels, hence bring forward the newly onset-AAbs. Below is an example of a plot, where the left panel shows robust z-scores based on the whole sample population, with subjects that are always above the threshold of 3.5 colored blue. The threshold is marked with a dotted line. Right plot shows the normalized log 2 MFI value, but colored based on the robust Z-scores to the right.

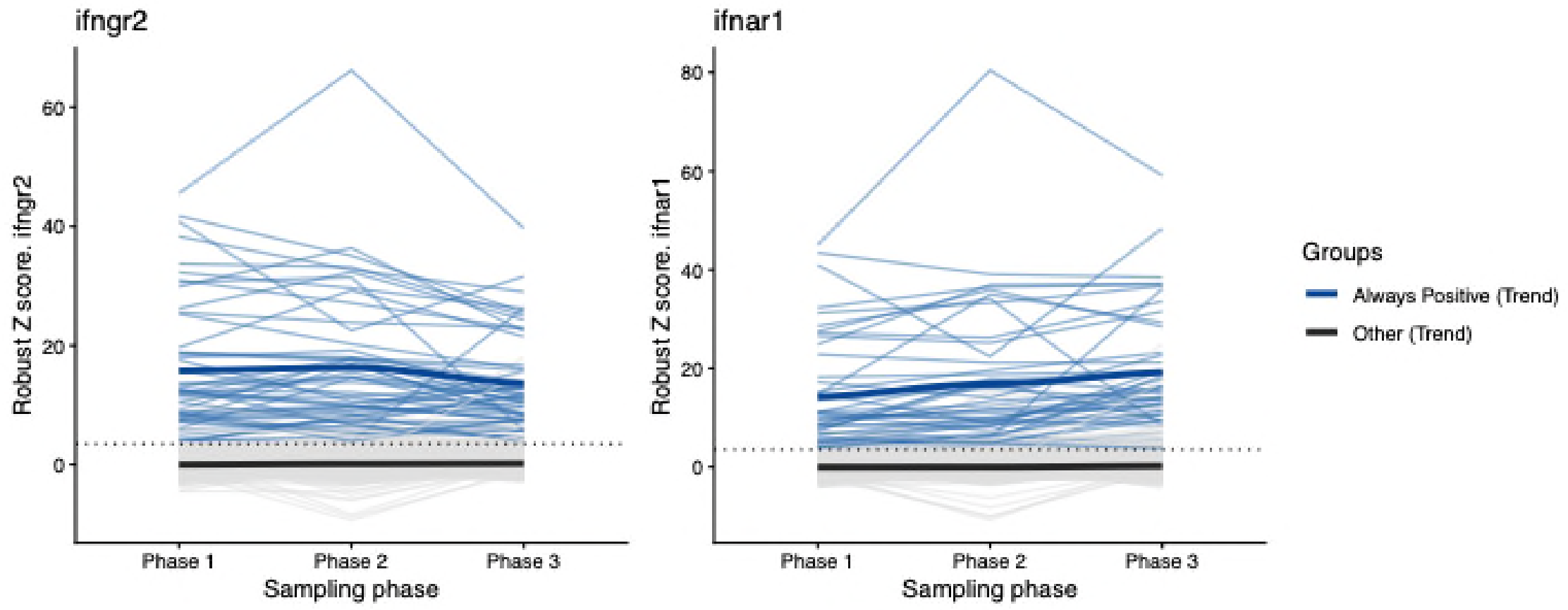

2. The longitudinal AAb profiling is a strong part of the paper, but for the association with prolonged COVID-19 symptoms, please clarify the multiple-testing correction used.

Answer: We agree with the reviewer and kindly point out our responses to comments 4 and 5 by Reviewer 1. To further strengthen these results, we have performed a multivariable logistic regression with IFNAR1 or IFNGR2 AAb-status as predictor and long covid symptoms (symptoms 2 months or 3 months) as outcome and added sex, BMI (normal weight vs overweigh/obese), serocluster belonging phase 3, and self-reported current or previous depression in phase 1 in the model, and used the AAb– group as reference. Of note, we do not have access to data regarding baseline immune dysregulation or pre-existing conditions that have been implicated in AAb production to interferons, besides self-reported current or previous depression from phase 1. The associations between IFNAR1 or IFNGR2 AAb status with long-term symptoms remained convincingly stable, and these results are now described in the Results section (lines 171ff) and in Supplementary Data 4.

3. Several results are currently presented mainly through p-values or FDR values. Reporting effect sizes with confidence intervals would make the biological relevance much clearer.

Answer: We agree with the reviewer, and even though effect sizes have previously been reported in the Supplementary Data files, we have now added estimates to the manuscript, also in line with comments from Reviewer 1.

4. Potential storage effects on the detection of SARS-CoV-2 antibodies and AAb should be clearly acknowledged. If these effects cannot be directly addressed analytically, the limitation should be discussed.

Answer: We thank the reviewer for this suggestion. As antibodies are among the most stable proteins in human blood, we have not seen any strong influence in storage time in our other studies compared to the exposure-related immune response.

As the response to exposure was substantial for SARS-CoV-2 and may vary heavily between individuals, this could be difficult to decompose, also considering some diminishing antibody-responses with time. Further, vaccination during phase 3 increased background in this phase, making this even

more difficult. We have therefore chosen to expand our discussion on this subject and added it as a clear limitation to the study (lines 461ff): *“The coinciding vaccination campaign, the start of the third (and final) sampling phase, and the emergence of the fast-spreading Omicron variant presented challenges for disentangling the longitudinal differences from storage effects across the three collection phases.“*

With regards to the AAb levels, we focused on those that were pre-existing and consistent over time. While discovering AAbs that appeared and disappeared, we did not have sufficient data to resolve unrelated and incidental increases in AAb levels. Since we are not using exact titers but z-scores relating to the tested population, any general decrease in AAb levels in the 2400 samples is already accounted for.

5. The Results section should be more descriptive about the assays performed. In some places, the description of the techniques are a bit vague. For example, please state the proteomic method used in the Results rather than relying on citations only.

Answer: We thank the reviewer and have taken this opportunity to revisit the Results section to clarify the used methods.

6. More information on LAP3 would be helpful, including additional discussion or speculation on possible mechanisms underlying its elevation after infection.

Answer: We agree with the reviewer and have expanded the discussion on LAP3 (lines 428 ff). As a note, LAP3 is an independently reproduced, IFN-induced, core-set COVID-19 blood biomarker that rises after infection and tracks tissue-level changes (Nat *Commun* 2023, doi:10.1038/s41467-023-41159-z).

7. Line 58: The statement that anti-SARS-CoV-2 antibodies persist “up to a year,” is inaccurate as we now know they can persist for well over a year after infection or vaccination.

Answer: We thank the reviewer for pointing this out; we have reformulated this statement and updated the reference.

Response to Reviewer #1 (Remarks to the Author):

REVIEWERS' COMMENTS

Reviewer #1 (Remarks to the Author): The remaining points are primarily related to transparency and presentation rather than substantive methodological concerns.

Comment to the Editor

The authors have addressed most of the substantive concerns raised in the first round of review with appropriate additional analyses and clarifications. In particular, they have provided further analyses addressing potential genetic confounding related to the IFNAR2 variant, the potential contribution of CBC parameters to the LAP3-infection association, multiple testing in the Aab-symptom analyses, and the methodological rationale and stability of the clustering approach. The additional analyses are generally reassuring. The overall methodological approach is strong.

I therefore consider the manuscript substantially improved. However, a few points would benefit from further clarification and/or incorporation into the manuscript or Supplementary Materials to ensure adequate transparency for readers, as detailed below.

>> We thank the reviewer for the positive assessment of our work.

Comments to the Authors

1) Sex-stratified analyses (in response to R1#10).

The additional sex-stratified analyses presented in the rebuttal are informative and appropriately interpreted, particularly given the marked imbalance in the number of males and females within the infected cohort. LAP3 remains significantly associated with infection in females, whereas the corresponding analysis in males does not reach statistical significance after FDR correction. Importantly, however, only one male participant contributed a sample within 30 days of PCR-confirmed infection, which is also the time window in which the strongest LAP3 elevation was observed. Thus, the absence of statistical significance in males cannot readily be interpreted as evidence of a sex-specific effect. Given the relevance of this analysis to the robustness and interpretation of the central LAP3 finding, please include the sex-stratified analyses in the Supplementary Materials, together with an explicit statement in the main text acknowledging the limited statistical power in the male subgroup.

>> We thank the reviewer for the comment and have added relevant information into the text lines 323ff.

2) rs2229207 variant and anti-IFNAR2 autoantibodies (in response to R1#5).

The additional analysis of rs2229207 carriage in relation to IFNAR2 autoantibody status and long-term COVID-19 symptoms is appropriate and reassuring. In particular, the authors report that inclusion of rs2229207 carriage in the relevant regression model does not materially alter the associations between IFNAR1/IFNGR2 autoantibodies and long-term symptoms. Since these analyses were performed specifically to address a potential source of genetic confounding, I recommend including the corresponding results as a supplementary table, with an appropriate reference in the main text. This would improve transparency and allow readers to assess the robustness of the findings without relying on the rebuttal letter.

>> We thank the reviewer for the and have added relevant information into the text lines 171ff, 687ff and to Supplementary Data 4 (as Model 3). We have also added the suggested reference (ref #18).

3) Language regarding temporal resolution and "transient" alterations (in response to R1#3).

I appreciate the authors' clarification that the sampling design is intended to capture epidemiological trajectories rather than provide fine-grained immunological time resolution. Nevertheless, I recommend a final careful review of the manuscript to ensure that terms such as "transient

alterations," "short-term variability," and similar temporal descriptions are consistently qualified throughout the text. This is particularly relevant because the most informative post-infection window contains a relatively limited number of observations and only a small number of participants were sampled very close to PCR-confirmed infection. The conclusions regarding temporal dynamics should therefore be framed in a way that is fully consistent with the temporal resolution actually supported by the data.

>> We thank the reviewer for the comment. We have looked through the text with this aspect in mind and believe the current description is clear in describing the terms dynamic and temporal in a transparent and reasonable manner. We though softened short-term to shorter-term.

4) ProtPQN versus standard Olink normalization (in response to R1#12).

The authors have clarified that Olink intensity normalization and ProtPQN serve complementary purposes and that both normalization steps are incorporated into the analytical workflow. This addresses the methodological rationale raised in the first round. Nevertheless, if readily available and without requiring substantial additional analysis, it would be useful to report whether the principal findings, particularly LAP3 and TIMP3, remain qualitatively unchanged when considering standard Olink normalization alone. This could be provided briefly in the Supplementary Materials or, alternatively, mentioned in the text as a robustness assessment. I consider this a useful additional check rather than a major methodological concern.

>> We thank the reviewer for the comment. We have introduced, evelauted and discussed ProtPQN in our previous works, including references # 18: Fredolini et al. (2024) Proteome profiling of home-sampled dried blood spots reveals proteins of SARS-CoV-2 infections. Commun Med 4, 55. The method was then called AbsPQN (now called ProtPQN to make it more generalizable) and demonstrated its capability to reduce variance introduced by layperson sampling and reduced random protein correlation drifts due to protein panels. Users can access the R package code on Zenodo (reference #54) to evaluate its effects on their respective data. For transparency, below we show the consistency between raw and processed data for the two proteins.

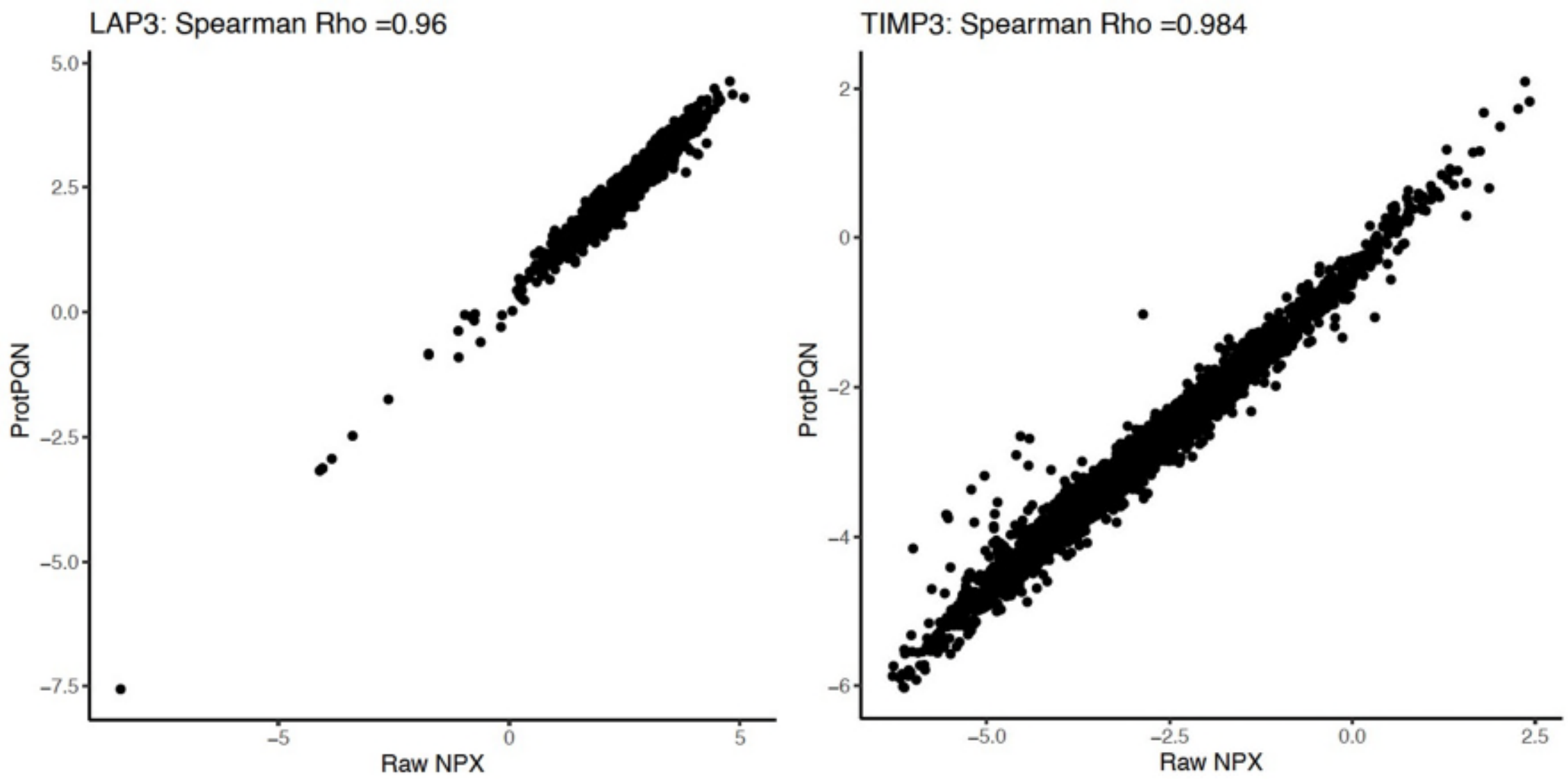

5) Remaining points.

The other issues raised in the first round of review have, in my view, been adequately addressed through the additional analyses and/or proposed revisions. The corresponding changes and clarifications are satisfactory.

>> We thank the reviewer for the constructive comments and support for improving our manuscript.
