# Supplementary material for: Molecular profiling of repeated self-sampled blood reveals dynamic immune phenotypes in young adults
Source: Nat Commun. 2026 Nov 14;17:11680. doi: 10.1038/s41467-026-78987-8 (PMC13769765; doi:10.1038/s41467-026-78987-8)
Supplement: Supplementary file 4 [file 41467_2026_78987_MOESM1_ESM.docx]

# SUPPLEMENTARY INFORMATION

**Molecular profiling of repeated self-sampled blood reveals dynamic immune phenotypes in young adults.**

Annika Bendes^1#^, Sophia Björkander^2#^‚ Maura M. Kere^2^, Simon Kebede Merid^2^, Ashish Kumar^2^, Leo Dahl^1^, Tess Pottinger^2^, Zhebin Yu^3^, Anna Gardell^1^, Amelie Vogt^1^, Changil Kim^4^, Qiang Pan-Hammarström^5^, Anna Bergström^3,6^, Inger Kull^2,7^, Anne-Sophie Merritt^3,6^,

Sandra Ekström^2,3,6^, Alexandra Lövquist^6^, Ben Murrell^4^, Niclas Roxhed^8,9^,

Erik Melén^2,7$^*, and Jochen M. Schwenk^1,7$^*

1. SciLifeLab, Department of Protein Science, KTH Royal Institute of Technology, Solna, Sweden; 2. Department of Clinical Science and Education, Södersjukhuset, Karolinska Institutet, Stockholm, Sweden; 3. Institute of Environmental Medicine, Karolinska Institute, Stockholm, Sweden; 4. Department of Microbiology, Tumor and Cell Biology, Karolinska Institutet, Stockholm, Sweden; 5. Division of Immunology, Department of Medical Biochemistry and Biophysics, Karolinska Institutet, Stockholm, Sweden; 6. Centre for Occupational and Environmental Medicine, Region Stockholm, Stockholm, Sweden; 7. Sachs' Children and Youth Hospital, Södersjukhuset, Stockholm, Sweden; 8. Department of Intelligent Systems, KTH Royal Institute of Technology, Stockholm, Sweden; 9. MedTechLabs, BioClinicum, Karolinska University Hospital, Solna, Sweden.

^#^These authors contributed equally

^$^These authors jointly supervised the work

* Corresponding authors: [erik.melen@ki.se](mailto:erik.melen@ki.se) and [jochen.schwenk@scilifelab.se](mailto:jochen.schwenk@scilifelab.se)

# SUPPLEMENTARY TEXT

## Multi-analyte serology

To enhance the certainty of our observations, we included multiple proteins for spike (S) and nucleocapsid (N), along with several receptor-binding domain (RBD) variants of S. Overall, the different variants showed a good agreement (Supplementary Fig. 2) with the mean Spearman’s rho being 0.83 (min = 0.78, max = 0.89) for N, 0.90 (min = 0.86, max = 0.92) for S, and 0.91 (min = 0.81, max = 0.96) for RBD. Classification of serostatus was determined using an analyte-specific, population-density-based cutoff calculated based on the two first sampling phases, assuming that most samples were collected before infection and/or vaccination.

The S protein, and especially the RBD, are mutation-prone, and we could therefore explore antibody recognition of the different RBD variants. The version that had overall lowest agreement with the others was the omicron variants. For RBD, we found that only ~75% of the subjects having antibodies against wildtype (wt) were also positive for the omicron variants (Supplementary Fig. 3), with omicron variant BA.1 having lower agreement with WT than Omicron BA.2. This is consistent with BA.1 exhibiting more escape from ancestral antibodies than BA.2 in neutralizing assays in plasma. The lower recognition of omicron variants compared to the earlier ones indicates the importance of continuing vaccine development for new virus variants.

The RBD variants with the highest positive proportion in Phase 1 were delta, alpha, WT, and kappa (mean = 15.0%, min = 14.7%, max = 15.3%), and both omicron variants BA.1 and BA.2 had a positive proportion below 2.7%. Delta, alpha, wt and kappa were also the variants with the highest proportion of seropositivity in Phase 2 (mean = 28.0%, min = 27.3%, max = 28.8%), and the two omicron variants having an average seropositivity of 6.7%. In Phase 3, all RBD variants have a seropositivity above 96.2%, with kappa having the highest percentage of seropositive subjects, and BA.1 having the lowest.

It is well-established that SARS-CoV-2 infection induces both anti-S and anti-N antibodies (S+N+), while vaccination triggers only anti-S antibodies. This explains the higher proportion of S+ samples compared to N+ in our cohort. By Phase 3, 93.4% of participants had been vaccinated, aligning with the high S+ and RBD+ prevalence. In contrast, only 18% had a PCR-confirmed infection, while we measured N+ seropositivity at 26.0%. This discrepancy may be due to asymptomatic infections or changes in national testing recommendations in Sweden in beginning of 2022. Among 226 samples collected after PCR-confirmed infection, 80.7% were N+.

Comparing the seropositivity against S variants for Phase 2 with the estimated seropositivity in plasma samples collected at the same time using ELISA showed a good agreement (Supplementary Fig. 4) with an average Cohen’s kappa value of 0.90, with 99.2% of the ELISA-positive subjects being classified as seropositive in the presented study against the wild-type Spike proteins. Spike omicron had slightly lower agreement with 82.4% of the ELISA-positive subjects being classified as positive using the presented approach, which is not surprising since Phase 2 was prior to the emergence of the omicron variant.

## Effect of time since an event

We investigated the trends in antibody levels in relation to time of event (Supplementary Figs. S5). For individuals with a PCR-confirmed infection, antibody levels increased within 30 days post infection (Fig. S5a). When investigating antibody levels since time since vaccination, antibody levels against S and RBD started to increase and reached the highest titers after 1-2 weeks post vaccination (Figs. S5b). Anti-N IgG levels remained below positivity threshold after vaccination (Fig. S5c), while repeated vaccination led to high antibody levels (Fig. S5d). In unvaccinated subjects with PCR-confirmed infection, the anti-S IgG levels remained above positive threshold for the whole 12-month period, while the anti-N IgG levels decreased gradually (Fig. S5e).

# Supplementary Figures

**Fig. S1**: PCR-confirmed infection across sampling phases. (a) Sampling date distribution for the different sampling phases (upper) and distribution of dates for PCR-confirmed SARS-CoV-2 infection (lower). Green color indicates samples belonging to sampling Phase 1, orange sampling Phase 2, and blue sampling Phase 3. (b) Percentage of samples with PCR-confirmed infection per sampling phase. Asterisks present FDR adjusted p-values below 0.05 when comparing proportions using Fisher’s exact test.

**Fig. S2**: **Comparing protein variants**. Spearman’s rho correlation values and signal distribution of IgG levels against different (a) nucleocapsid proteins, (b) spike variants, and (c) receptor-binding domain (RBD). Each dot is a sample.

**Fig. S3: Agreement across RBD variants**. The percentage of positive samples for RBD wt variant (rbd.wt.sars.2) that were also positive for the other RBD variants. Omicron variant and RBD from SARS-CoV-1 have the lowest agreement to RBD SARS-CoV-2 wt. Dashed line indicates 75%.

**Fig. S4: Agreement between Luminex and ELISA classification**. The number of subjects from Phase 2 that were classified as seropositive or negative in the serology data from the Luminex assays using a population-based density cutoff, or in the commercial SARS-CoV-2 Spike protein ELISA.

**Fig. S5**: **Antibody response to infection and vaccination**. Antibody-binding to N, RBD wild-type (wt) and S-wt **(a)** pre-, during and post-PCR confirmed infection and **(b)** pre-and post-vaccination. Samples from subjects who got vaccinated have been excluded from A, and samples from N+ positive subjects have been excluded from B. Lines connect samples taken from the same subject. Dotted vertical lines indicate days 0 and 30 post PCR+ or vaccination. Grey color indicates that samples were taken pre infection or vaccination. Red indicates that the samples were taken **(a)** within 30 days of infection **(b)** or within 5 days of vaccination, dark orange indicates that the samples were taken between 6 to 10 days post vaccination, light orange indicates that the samples were taken between 11 to 30 days post vaccination, and yellow indicates that the samples were taken after **(a)** 30 days post infection or **(b)** vaccination. Effect of time since first vaccination event on the levels of **(c)** anti-N and **(d)** anti-S antibodies. Data trends are visualized in yellow using local regression (LOESS). The shaded area represents the 95% confidence interval around the smoothed mean. **(e)** Effect of time since any PCR-confirmed infection on the levels of anti-N antibodies excluding samples taken post vaccination. Orange and blue curves show the non-linear trends from LOESS regression for spike and nucleocapsid, respectively. Horizontal lines show antigen-specific cutoff-values for seropositivity.

**Fig. S6**: Correlation heatmap showing pairwise correlations of anti-IFN prevalences within sampling **(a)** Phases 1, **(b)** Phase 2, and **(c)** Phase 3. Red represents positive correlation values, whereas blue represents negative Spearman correlation values.

**Fig. S7: Global analysis of proteomics data.** **(a)** PCA plot of the DBS proteomics data is labeled by sampling phase with density distributions shown to the right and above. Sampling phases are shown in teal for phase 1, orange for phase 2 and steel blue for phase 3. **(b)** Histogram of the distribution for the Spearman rho correlations between proteins and Principal Component 1 (PC1). The dotted lines indicate rho = |0.5| in yellow and rho = 0 in grey. **(c)** Heatmap of Spearman rho correlations between the Principal Components 1-2 (PC1-3) and clinical variables.

**Fig. S8**: **Comparison of protein-trait associations in BAMSE and UK biobank (UKB).** Slopes (β coefficients) from BAMSE are shown on the y-axis, and corresponding slopes from UKB on the x-axis for **(a)** age, **(b)** BMI, **(c)** sex and **(d)** smoking. Each point represents a protein-trait association, and colors indicate significance: orange for associations significant only in BAMSE, steel blue those significant only in UKB, green for associations significant in both, and grey for non-significant in either dataset. Only proteins measured in both studies are included. Dashed horizontal and vertical lines represent a slope of zero.

**Fig. S9:** **Comparison of protein-trait associations in BAMSE and population survey.** Slopes (β coefficients) from BAMSE are shown on the y-axis, and corresponding slopes from another DBS population study^16^ on the x-axis. Each point represents a protein-trait association for **(a)** age and **(b)** sex. The colors indicate significance: orange for associations significant only in BAMSE, steel blue those significant only in the other DBS paper, green for associations significant in both, and grey for non-significant in either dataset. Only proteins measured in both studies are included. Dashed horizontal and vertical lines represent a slope of zero.

**Fig. S10:** **Changes of protein levels in relation to storage time.** **(a)** Slopes from linear mixed effects models for seronegative subjects in all three phases (n=25) plotted against Bonferroni-adjusted p-value. Horizontal dashed line marks Bonferroni p=0.01. **(b)** Distribution of the slopes form the model. Colors indicate if the protein is significantly up or down regulated using Bonferroni p<0.01 as significance threshold. **(c)** Distribution of storage time-related protein alterations across protein classes. Bars represent the total number of proteins analyzed per class from the Human Protein Atlas , with the blue fraction highlighting the proportion of significantly altered proteins. Significance brackets indicate FDR-adjusted p-values from pairwise Fisher’s exact test (* p< 0.05, ***p< 0.001). From top to bottom, the exact p-values were p=0.0097, p=0.037, p=0.014, and p=0.000028.

**Fig. S11. Distribution of complete blood counts (CBC).** Vertical red dashed lines indicate the non-sex-specific reference limits (upper and, when applicable, lower) by Karolinska University laboratory. When applicable, pink and blue lines mark female- and male-specific reference values, respectively. EVF = erythrocyte volume fraction.

**Fig. S12.** The number of correlations that remained significant after multiple testing correction (FDR<0.05) for each CBC parameter and the overlap of proteins between parameters.

**Fig. S13:** **Strongest positive and negative protein correlations for each complete blood count (CBC)**. Y-axis: protein levels after protein probabilistic quotient normalization (ProtPQN), X-axis: CBC parameter. Only correlations surviving multiple testing correction (FDR<0.05) are shown, with Spearman’s rho, FDR-adjusted p-value, and R^2^ from a fitted linear model. Note that the axis scales vary across plots. EVF= erythrocyte volume fraction.

**Fig. S14:** **Most significant protein associations for each complete blood count (CBC)**. Y-axis: protein levels after ProtPQN normalization; X-axis: CBC parameter. Only associations surviving multiple testing correction (FDR <0.05) are shown, with slope and FDR-adjusted p-value from a crude linear regression (protein~CBC parameter) and R^2^ from a fitted model. Note that the axis scales vary across plots. EVF= erythrocyte volume fraction.

**Fig. S15: Comparison of plasma and DBS levels.** The heatmap shows the correlations of 51 proteins measured both previously in 690 plasma samples at the BAMSE 24-year follow-up (x-axis; “_B24”) and in 782 DBS samples from Phase 2 (y-axis; “_C19”). Cell color represents correlation strength and direction (Spearman rho); asterisks mark significant correlations (FDR<0.05).

**Fig. S16:** Correlations between complete blood cell count with differential (CBC) parameters and proteins at the BAMSE 24-year follow-up and Phase 2 of the COVID-19 follow-up, showing only correlations that remained significant after multiple testing correction (FDR<0.05). Analyses include the overlapping 690 subjects at 24 years and 782 subjects at Phase 2 with complete CBC and protein data.

**Fig. S17: Spearman and intraclass correlation across repeated samplings**. Density plots of **(a)** Spearman’s rank correlation (Rho) and **(b)** intraclass correlation coefficient (ICC3) for all Olink proteins across different sampling phases. The green curve represents Phase 1 vs Phase 2 (green), Phase 1 ~ Phase 3 (orange), and Phase 2 ~ Phase 3 (blue). **(c-j)** Protein levels across the three sampling phases of n=807 donors (= 2394 samples) for selected proteins with high (blue) and low (red) ICCs. Each dot represents a sample, and lines connect samples from the same individual. **(k)** Distribution of Spearman correlation coefficients between paired samples from the same subject (olive) and randomly paired samples from different subjects (purple). **(l)** Distribution of Spearman correlation coefficients between sampling phases for two groups: individuals who changed serocluster across time points (top panel) and those who remained in Serocluster 1 throughout (bottom panel). Colors indicate phase comparisons as in panels **(a)** and **(b)**.

**Fig. S18: Protein levels in seronegative versus infected subjects.** **(a)** Top proteins with significant difference over time between infected and seronegative subjects from bootstrapping the seronegative population over 100 iterations. Color indicates the direction of the protein change. **(b)** Differences in protein levels between seronegative subjects who remained seronegative and seronegative subjects who became infected in sampling Phase 2, analyzed within each sampling phase. Color indicates the proportion of iteration in which the protein was significant. The horizontal line denotes FDR=0.05 threshold. **(c)** LAP3 levels with time since PCR-confirmed infections (n=137 donors, circles), excluding samples collected from post-vaccination subjects. The asterisks indicate significant differences (FDR<0.05), and as appropriate, by either Wilcoxon’s rank-sum (unpaired) or signed-rank (paired) tests. Comparing pre~0-30 resulted in p=0.035, while 0-30~30 resulted in p=5.97x10^-5^. The boxplots mark the median and interquartile range (boxes from 25% to 75%). Whiskers extend to at most 1.5 x IQR from the boxes. **(d)** LAP3 levels with time since PCR-confirmed infections including samples from vaccinated subjects (n=144 donors, triangle). Comparing pre~0-30 resulted in p=3.96x10^-9^, while 0-30~30 resulted in p=4.95x10^-8^. **(e)** LAP3 levels against days since the closest vaccination dose (n=1141 samples).

**Fig. S19: Proteins in relation to infection and vaccination**. **(a-d)** Within-phase comparisons between subject groups defined by infection and vaccination status. Groups are labeled by the principle serocluster (SC) in Phase1 serocluster in Phase 2” **(a)** Subjects infected prior to Phase 1 (labeled SC2~SC2) versus subjects who remained uninfected and unvaccinated through Phases 1 and 2 (labeled 11), bootstrapped over 100 iterations. Colors indicate the proportion of iterations in which a protein was significantly different between groups. The dashed horizontal line indicates an FDR threshold of 0.05. **(b)** Subjects infected prior to Phase 1 (labeled SC2~SC2) versus subjects uninfected in Phase 1 who became infected before Phase 2 (labeled SC1~SC2). **(c)** Previously infected subjects who became vaccinated prior to Phase 2 (labeled SC2~SC3) versus seronegative subjects who became both infected and vaccinated prior to Phase 2 (labeled SC1~SC3). **(d)** Previously infected subjects with waning anti-N antibodies who became vaccinated prior to Phase 3 (labeled SC2~SC4) versus seronegative subjects who were vaccinated only prior to Phase 3 (labeled SC1~SC4), bootstrapped over 100 iterations. **(e-h).** Protein levels across sampling phases. **(e-g)** Levels of TRAF2 **(e)**, LGALS9 **(f)**, and LAP3 **(g)** in Phase 1 and 2 for subjects (n=173) infected prior to Phase 1 (labeled SC2~SC2) versus subjects uninfected in Phase 1 who became infected before Phase 2 (labeled SC1~SC2). **(h)** LAP3 levels in Phase 2 and 3 for previously infected subjects (n=190) who became vaccinated prior to Phase 3 (labeled SC2~SC3), versus seronegative subjects who became both infected and vaccinated prior to Phase 2 (labeled SC1~SC3). The boxplots mark the median and interquartile range (box from 25% to 75%). Whiskers extend to at most 1.5 x IQR from the boxes.
